# Multiple novel promoter-architectures revealed by decoding the hidden heterogeneity within the genome

**DOI:** 10.1093/nar/gku924

**Published:** 2014-10-17

**Authors:** Leelavati Narlikar

**Affiliations:** Chemical Engineering Division, National Chemical Laboratory, Dr. Homi Bhabha Road, Pune 411008, India

## Abstract

An important question in biology is how different promoter-architectures contribute to the diversity in regulation of transcription initiation. A step forward has been the production of genome-wide maps of transcription start sites (TSSs) using high-throughput sequencing. However, the subsequent step of characterizing promoters and their functions is still largely done on the basis of previously established promoter-elements like the TATA-box in eukaryotes or the -10 box in bacteria. Unfortunately, a majority of promoters and their activities cannot be explained by these few elements. Traditional motif discovery methods that identify novel elements also fail here, because TSS neighborhoods are often highly heterogeneous containing no overrepresented motif. We present a new, organism-independent method that explicitly models this heterogeneity while unraveling different promoter-architectures. For example, in five bacteria, we detect the presence of a pyrimidine preceding the TSS under very specific circumstances. In tuberculosis, we show for the first time that the spacing between the bacterial 10-motif and TSS is utilized by the pathogen for dynamic gene-regulation. In eukaryotes, we identify several new elements that are important for development. Identified promoter-architectures show differential patterns of evolution, chromatin structure and TSS spread, suggesting distinct regulatory functions. This work highlights the importance of characterizing heterogeneity within high-throughput genomic data rather than analyzing average patterns of nucleotide composition.

## INTRODUCTION

The last decade has seen remarkable advances in high-throughput sequencing technologies, making them both fast and cost-effective. As a consequence, apart from simply sequencing (or re-sequencing) genomes, these technologies have been successfully applied to probe various biochemical activities of the genome at a single nucleotide resolution. One such activity is transcription initiation by the RNA polymerase. In a given cell-type of interest, methods like cap analysis of gene expression (CAGE) (1), oligo-capping (2), cap-trapping (3) and Rapid Amplification of cDNA Ends (5′-RACE) (4) coupled with high-throughput sequencing identify transcription start sites (TSSs) associated with the transcriptome. These methods differ in the manner in which they distinguish a true site of initiation from a 5′ end generated by RNA cleavage or degradation (5), but they typically produce robust genome-wide maps of TSSs (6).

Genome-wide maps of TSSs, by themselves, provide a wealth of information such as cell-type specific usage and spatial distribution of TSSs for every transcribed gene (7). Further downstream analyses typically focus on the genomic information at the TSSs to understand the mechanism of transcription initiation. In eukaryotes, TSS neighborhoods are assessed for GC-richness and prevalence of known promoter elements such as the TATA-box, INR element, etc. (8–10). The presence or absence of these features is then tested for association with the expression level and the spatial distribution of TSSs. For example, promoters with TATA-boxes have been shown to have more narrow peaks, with transcription initiation more likely to happen specifically at one position. Similarly, promoters in CpG islands are believed to have a more broad expression pattern characteristic of housekeeping genes (11). However, this analysis is highly restrictive because of two reasons. First, only “known” features are tested. A previously uncharacterized element can never get identified with this approach. Second, it is not reasonable to assume that features function separately, independent of other features. For example, Feature A may behave in a coordinate fashion with Feature B, but not with Feature C. Identification of such “modules” has been attempted before (12,13), but their success, again, depends on which features were considered while building modules in the first place.

To get around this issue, de novo motif discovery is conducted using methods like MEME (14) or Chipmunk (15). This technique has been successful in identifying novel core promoter elements in the fly (13). However, in this approach, motifs that are present only in a small fraction of sequences are likely to be missed. This is primarily because these de novo motif discovery programs have been developed to tackle a different problem. The original goal in these programs was to find motifs that are statistically overrepresented in the entire input set, and therefore are more suited for finding common patterns in coregulated promoters or chromatin immunoprecipitation data. However, promoters can have diverse mechanisms of regulation and all promoters are not likely to be controlled by the same set of factors. This heterogeneity can result in no motif being statistically overrepresented in the whole set of promoters. It is important to note that the aforementioned high-throughput experiments do not probe any specific component of the regulatory system, but target the 5′ ends of all transcripts. The neighborhood around an identified TSS can be different from that of another TSS. Furthermore, most of these methods do not make explicit use of the TSS location during the learning and a motif is considered significant as long as it appears somewhere in the input sequence. However, when identifying promoter elements which determine location of transcription initiation, this information is not only highly pertinent, but is increasingly more available with the high resolution of the high-throughput experiments.

We present a novel approach for identifying heterogeneous promoter-architectures from high-throughput TSS data. This method is not specific to any experimental protocol: it only uses the identified TSS location and the genomic sequence around it. It makes no assumptions about the number of architectures, the number of promoters with a certain architecture or the prevalence of any motif. Instead, we treat this as an unsupervised machine learning problem, where promoter sequences are clustered into groups having similar architectures, while simultaneously identifying positions along promoters that define each architecture. Since it does not use any prior information regarding motifs or promoter elements, it is inherently organism-independent. We demonstrate its utility in identifying novel promoter-architectures in three different species of bacteria: *M. tuberculosis*, *E. coli* and *K. pneumoniae*, as well as in two eukaryotes: fly and human. By combining information from other biological sources we show that these architectures are likely to have distinct regulatory roles.

## MATERIALS AND METHODS

### Model description

We consider the problem of partitioning *n* DNA sequences $\bf {X}_1,\ldots ,\bf {X}_n$, each of length *l*, into *k* different architectures *a*_1_, *a*_2_, …, *a*_*k*_. $X_i^j$ represents the *j*th nucleotide in the *i*th sequence $\bf {X}_i$ where 1 ≤ *j* ≤ *l*. We assume that each architecture *a*_*u*_, where 1 ≤ *u* ≤ *k*, has a few key important positions denoted by the set $I_{a_u} \subset \lbrace 1,2,\ldots ,l\rbrace$.

We learn a probabilistic model *M* and its parameters Θ for finding the optimal partition. The structure of the model *M* is characterized with:

1. the number of architectures *k* and
2. the number of important positions in each architecture *a*_*u*_, i.e. $|I_{a_u}|$.

Once the structure is fixed, the parameters of the model can be defined as follows:

1. *y*_*i*_ represents the architecture to which sequence *X*_*i*_ belongs and is modeled using a categorical distribution γ over {1, 2, …, *k*}.
2. Each position is modeled using a categorical distribution over the four nucleotides. For architecture *a*_*u*_, we have $|I_{a_u}|$ categorical distributions specific to that architecture, which are parameterized by $\phi _{a_u^{j}} = [\phi _{a_u^{j}}({\mathtt {A}}), \phi _{a_u^{j}}({\mathtt {C}}),\phi _{a_u^{j}}({\mathtt {G}}),\phi _{a_u^{j}}({\mathtt {T}})]$, $\forall j \in I_{a_u}$. $\phi _{a_u^{j}}({\mathtt {A}})$ is the probability of finding the nucleotide A at position *j* in architecture *a*_*u*_ and similarly for C, G and T. All other positions within architecture *a*_*u*_ are parameterized by a background categorical distribution $\phi _0^{j}$ where $j \not\in I_{a_u}$. Note that this background distribution is independent of *a*_*u*_ and therefore is applicable to all architectures for which *j* is not an important position.

We can compute the likelihood of the sequence *X*_*i*_ as a simple product of categorical probabilities:

(1) \begin{eqnarray*} P({\bf X_i}\mid M,\Theta ) &=& P({\bf X_i}\mid M, y_i=u, \bf {I}, \bf {\phi } ) \nonumber \\ &=& \left(\prod \limits _{j\in I_{a{_u}}} \phi _{a_u}^{j}(X_i^j)\right) \left(\prod \limits _{j\not\in I_{a{_u}}} \phi _{0}^{j}(X_i^j)\right) \end{eqnarray*}

and the likelihood of the full data as:

(2) \begin{eqnarray*} P({\bf X}\mid M,\Theta ) & = & \prod \limits _{i=1}^{n} P({\bf X_i}\mid M,\Theta ). \end{eqnarray*}

### Model learning

Assuming that the structure of the model is fixed, the goal is to compute the value of Θ that optimizes the posterior distribution:

(3) \begin{eqnarray*} P(\Theta \mid {\bf X}, M) & \propto & P({\bf X}\mid M,\Theta ) \times P(\Theta \mid M). \end{eqnarray*}

We use the conjugate Dirichlet prior over all categorical distributions, i.e., γ and $\phi _{0\ldots k}^{1\ldots l}$. The prior is symmetric with all pseudocounts set to 1.

We use collapsed Gibbs sampling (16) to draw samples iteratively from the posterior distribution in Equation (3). In every iteration, we hold out a sequence *X*_*i*_ and conduct two sampling steps: (1) sample the architecture identity *y*_*i*_ and (2) sample the important positions characterizing the recently sampled architecture. Both steps are carried out by analytically integrating out γ and $\phi _{0\ldots k}^{1\ldots l}$. After each iteration, the highest scoring set of parameters is retained. The whole process is conducted several times and the over-all highest scoring model is reported.

### Model selection

Since we do not know *a priori* the structure of the model, we determine it by varying the total number of architectures *k* and the number of important positions for each architecture. As both increase, the total likelihood in Equation (2) will theoretically increase, potentially resulting in overfitting. To avoid this, we select the model that achieves the highest likelihood in an unseen test set: we conduct a standard 5-fold cross-validation process, where the model is learned on four-fifths of the data and the likelihood is computed on the held out one-fifth set. This process is repeated five times so that each sequence is tested once and used for training four times. The model with the highest cross-validation likelihood is selected as the final model.

### Models used as classifiers

For classifying a genomic sequence *s* as better represented by model *M*_1_ with parameters Θ_1_ or model *M*_2_ with parameters Θ_2_, we compute the log odds score as:

(4) \begin{eqnarray*} \text{log odds score}(s) & = & \log \frac{P(s\mid M_1,\Theta_1)}{P(s\mid M_2,\Theta_2)}, \end{eqnarray*}

where both likelihoods are computed using Equation (1). A higher score implies a better fit with *M*_1_, while a low score implies a better fit with *M*_2_.

### Simulated datasets

Three sets were simulated. Each had a 1000 sequences with a length of 100 bases. The first set had five different architectures, three of which were governed by 10 important positions and two of which were governed five important positions. The positions for each architecture were randomly sampled from the 100 possible positions, independent of the other architectures. The parameters for the categorical distributions at the important positions were sampled from a Dirichlet distribution with all αs set to 0.1. This ensured distributions that preferred one nucleotide over the other three. All other positions for each architecture had uniform probabilities of {A,C,G,T}.

The second set had only one architecture, where all positions had a uniform distribution of nucleotides. This is similar to not having any motif. The third set also had only one architecture, but the distribution over nucleotides was non-uniform: the parameters for the categorical distribution for each position were sampled from a Dirichlet distribution, the αs for which were first sampled uniformly from 0.001 to 1. This implied different, low-entropy distributions at different positions, but no variation across sequences.

### Biological datasets

#### Bacteria

Mtb TSS data in exponential growth and starvation condition were taken from Cortes *et al.* (17). The sequences corresponding to −45 bp upstream and +5 bp downstream from the TSS were extracted from reference H37Rv sequence. The data for *E. coli* and *K. pneumoniae* was taken from Kim *et al.* (18) where a total of 3746 and 3143 TSSs two bacteria are reported, respectively. Of these, we identified a total of 2654 and 2339 as primary TSSs: this included those TSSs that were associated to some gene by the authors. In case of more than one TSS for a gene, the TSS with maximum tags was chosen.

#### Fly

We used two fly datasets. The first was from Ni *et al.* (8), which was reported using PEAT. They have categorized all TSS clusters within non-coding regions as NP, BP and WP based on the span of the cluster. The second dataset was from modENCODE where Hoskins *et al.* (9) report an integrated map of TSSs after combining data from high-throughput CAGE, RACE and EST data, which we refer to as the C-R-E dataset, to distinguish from the PEAT dataset. They report a probabilistic distribution of TSSs across each neighborhood. From this distribution, they compute an entropy-based shape index to characterize the TSS spread. In both datasets, the −45 to +45 region around the reported modal position was extracted using the dm3 build (19). One sequence each in the NP and WP set had unsequenced Ns; these were removed from the analysis.

#### Human

The latest processed FANTOM5 data (10) was used for the human genome. For each identified TSS cluster, this included the number of tags in the 100 bp neighborhood of the modal position and the entropy computed from tags across 517 cell-types. The entropy lies in the range of 0 (most cell-type specific) to log_2_517 (broad expression). The number of tags in the 100 bp neighborhood were used to compute the interquantile range (IQR) for each promoter. We used the 15 745 TSS clusters that had at least 100 tags as has been done before (20). For every reported TSS cluster, the −45 to +45 region from the modal position was extracted using the hg19 build; this resulted in non-repetitive 91 bp regions for 12 475 promoters.

#### Settings for JAPL and downstream analysis

JAPL was applied to learn all models with *k* varied from 1 (single architecture) to 13. In the case of bacteria the number of important positions were taken from the set {10, 20, 51}. In the case of the eukaryotes, since we had longer sequences, the number of positions were taken from the set {10, 20, 50, 91}.

PhastCons (19) scores across 15 insects relative to *Drosophila* and across 46 vertebrates relative to human were used for the conservation analysis. H2A.Z nucleosome positioning data from drosophila embryos was taken from Mavrich *et al.* (21). They report 146 bp nucleosome locations along with the number of reads associated with each nucleosome. The position corresponding to the middle of the nucleosome was assigned a score equal to the number of reads, while the 73 bases on each side were given a linearly interpolated score between 0 and the number of reads. In the case of human, we used the nucleosome signal for two ENCODE tier-one cell-types GM12878 and K562 directly (22).

For fly and human TSSs associated with RefSeq genes, we used DAVID (23) to identify enriched GO-terms: GOTERM_BP_FAT for biological processes, GOTERM_CC_FAT for cellular components and GOTERM_MF_FAT for molecular functions. To account for multiple hypothesis testing the Bonferroni corrected *P*-values are reported. The full data is available in Supplementary Tables S1 and S2. For Mtb, the TB Database (24) was used for enrichment analysis. When comparing significance of features specifically associated with different architectures like TSS peak height, number of tags, entropy, GC content etc., which are measured using real numbers, the non-parametric Wilcoxon rank sum test is used. All downstream analysis was done in R, while JAPL is written in C.

## RESULTS

### JAPL: joint architecture and position learning

We define the problem of identifying hidden promoter-architectures as one of finding an optimal partitioning of promoter sequences, where each partition is characterized by a different distribution over the alphabet {A,C,G,T}. We assume that each promoter sequence possesses one of *k* distinct architectures. Each architecture has a set of important positions with probability distributions distinguishing it from other architectures. For example, an architecture may be characterized by a 6 bp TATA-box at −25 position and a specific 10 bp downstream element at +12 position. Only these 16 positions are considered important in the model, others are “unimportant” for characterizing that architecture. Each unimportant position follows a background probability distribution, which is common across *all* architectures for which the same position is also unimportant. We do not assume any prior knowledge about the identity or distribution of any of the important/unimportant positions. If the structure of the model is fixed, i.e. *k* is known and the number important positions per architecture is known, identifying the architectures amounts to parameter estimation: (1) the important positions for each architecture along with their distribution parameters and (2) the background distribution for all positions. We use Gibbs sampling for this purpose. Since we do not know *a priori* the value of *k* or the number of important positions, we learn a set of models by varying both quantities and determine the optimal model through cross-validation.

**Figure 1.**
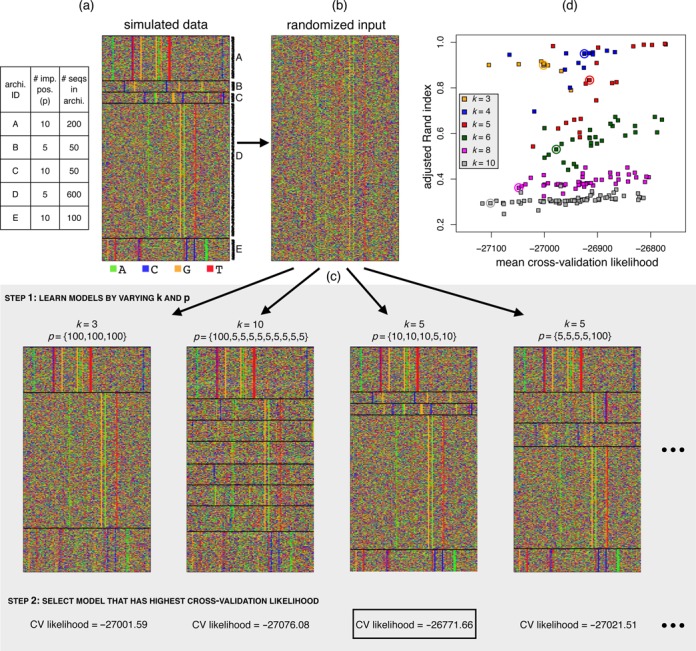
Illustration of JAPL applied to a simulated dataset. (a) A total of 1000 sequences of length 100 were simulated from five different architectures A–E, details of which are described in the table. (b) The sequences are reordered randomly. (c) JAPL is run on the permuted set. The number of architectures *k* is varied across {3, 4, 5, 6, 8, 10}, while the number of important positions are varied across {5, 10, 100} (step 1). Final model is selected based on the highest cross-validation accuracy (boxed likelihood value in step 2). A few representative learned models are displayed. When *k* is smaller than 5 (first model) only the two biggest architectures are correctly identified. When *k* is larger (second model), the architectures are again not identified properly. When *k* = 5 and the number of important positions in each architecture is close to truth, all architectures are identified (third model). Overestimating the number of important features can cause spurious signals to be picked up (fourth model). (d) Average ARI over all cross-validation folds is plotted versus the corresponding average likelihood score. For the true value of *k* (red), the best likelihood corresponds to an ARI close to 1. The circled points show models that consider all 100 positions to be important. These models are unable to distinguish between signal and noise, achieving both, low ARI values and low likelihoods.

As an example, we simulated a set of 1000 sequences with five different architectures (Figure 1a) containing varying numbers of artificial promoters. Each architecture is governed by 5 or 10 important positions, randomly sampled during the simulation. All unimportant positions were sampled from a uniform distribution over {A,C,G,T}, while all important positions were sampled from a categorical distribution, different from uniform (Materials and Methods). The sequences were randomly ordered after which the largest architecture, containing 60% of the sequences, dominates (Figure 1b). We believe this emulates the scenario in real situations where a majority of promoter sequences can weakly be explained by one or two rules. The goal here is to identify all the smaller architectures as well.

JAPL is run with different values of *k* (drawn from {3, 4, 5, 6, 8, 10}) and combinations of number of positions *p* (drawn from {5, 10, 100}) resulting in many different models (step 1 in Figure 1c). After learning all models, the models are assessed for their fit using 5-fold cross-validation and the model achieving the highest average log likelihood is selected as the final model (step 2 in Figure 1c, Materials and Methods).

The Rand index is commonly used for comparing two groupings of elements. The adjusted Rand index (ARI) is the corrected-for-chance, more robust measure of the same (25). A perfect grouping—one that matches with the simulated partition—achieves an ARI of 1 while a random grouping gets an ARI of 0. We note that the cross-validation likelihood value correlates with the ARI (Figure 1d). The highest likelihood is achieved for the true underlying value of *k* = 5, where the ARI is also close to 1. When all positions are considered important, which is inherently the case in methods like *k*-means or hierarchical clustering, the models achieve neither high ARIs nor high likelihoods. This suggests that when the underlying important features are only few, using all features for identifying heterogeneity can result in incorrect partitions.

To test the robustness of JAPL, we explored two likely scenarios. First, we assess the effect of not having learned and tested a model with the true underlying structure. We have shown that position selection is important, but testing all possible models is not feasible. We only test a few reasonable combinations of *p*. The original simulated set had architectures with 5 or 10 important positions. But we restricted the method to learn models with 20 important features. The ARI is still close to 1 for *k* = 5. As we increase the number of useful positions further to 30, 40 and 50, both the likelihood and the ARI decrease, as expected (Supplementary Figure S1a). Second, we tested whether the method was able to correctly detect the situation when there is a single underlying architecture with no heterogeneity. We simulated two datasets for this. The first one had all sequences drawn from a uniform distribution over {A,C,G,T}, emulating the scenario when there is no motif in the set, while in the second set, each position had a different distribution, but with no variation across sequences (Materials and Methods). We applied JAPL to both datasets with different values of *k* and *p*, but for each, the best likelihood was achieved at *k* = 1, indicating that the method can decode the correct number of hidden architectures (Supplementary Figure S1b).

Based on these observations, in the rest of the sections, when we apply JAPL to real data, we report the model selected according to the best cross-validation likelihood. Note that it is computationally infeasible to search through the space of all possible structures of models. Supplementary Figure S1a suggests that the accuracy of the learned model is more sensitive to the number of architectures *k* than to the precise number of important positions. We therefore limit the number of models to be learned by exploring all reasonable values of *k*, but with *p* drawn from approximately {all, half, …, 10} of the total positions.

### Bacteria

Cortes *et al.* (17) recently published a genome-wide map of TSSs in *Mycobacterium tuberculosis* (Mtb) under exponential growth as well as in a starvation model of growth arrest using dRNA-seq (26). They identify a total of 4164 and 4133 TSSs in the two conditions, respectively. Of these, 1778 TSSs in exponential growth and 1707 TSSs under starvation have been classified as “primary”: these are TSSs that can be assigned to an annotated gene. In addition to the positions of the TSSs, they also quantify the expression level of each TSS.

**Figure 2.**
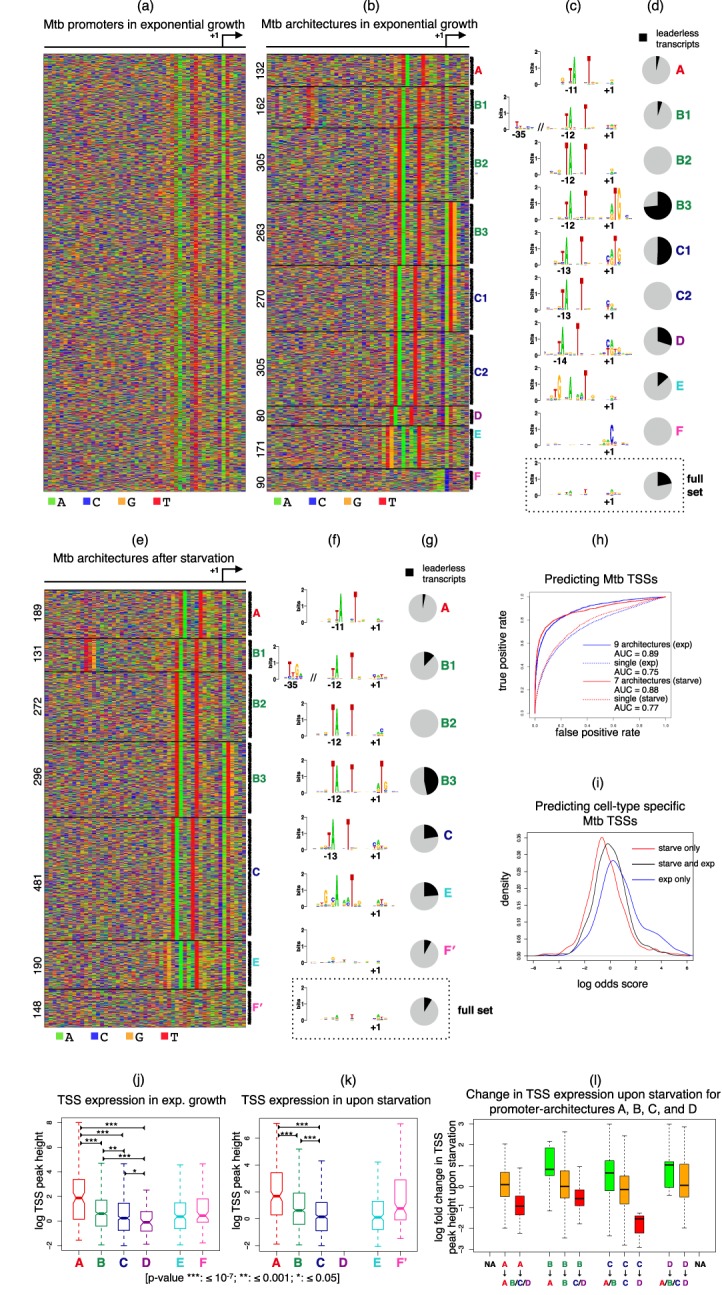
JAPL applied to Mtb. (a) −45 bases upstream and 5 bases downstream of all identified TSSs are aligned and ordered according to their position on the genome. Some signal can be identified visually within the 15 bp region near the TSS. (b) JAPL identifies nine different architectures, labeled A–F, ordered according to the spacing λ between the TANNNT motif and the TSS. The number of sequences in each architecture is shown on the left. (c) Logos and (d) fraction of leaderless transcripts are illustrated for each architecture; the bottom dotted picture is computed from all promoters. Note the presence of a pyrimidine at the −1 location in C1, C2 and D, where λ > 12. (e) JAPL identifies seven architectures in Mtb after starvation. (f) Logos and (g) fraction of leaderless transcripts are illustrated for each Mtb starvation architecture; the dotted picture is computed from all promoters. (h) Both models have predictive power in identifying TSSs that are not part of the training data. This is illustrated by using ROC curves (blue for exponential and red for starvation conditions) and the area under the curves (AUC). Simple, single architecture models perform worse than the models learned by JAPL. (i) Although the two models are similar, some TSSs are expressed in only one of the two conditions. The density of the log odds scores computed from nine and seven architecture models shows that TSSs expressed only during starvation have a low score (red), TSSs expressed only during exponential growth have a high score (blue) and TSSs expressed in both conditions have scores in between. Boxplots of the expression level of TSSs in each architecture show the importance of λ in (j) exponential growth as well as (k) after starvation. (l) Boxplots show the fold change in TSS expression after starvation when λ decreases (green), remains the same (orange), or increases (red) for genes with architectures characterized by a λ. The number on top of each boxplot denotes the number of TSSs in that category.

In their analysis, Cortes *et al*. use MEME and identify two dominant classes of primary promoters based on the presence (73%) or absence (23%) of the −10 motif TANNNT. Since the extended -10 motif has been shown to occur in some mycobacteria (27), they further split the first class based on variations within three bases preceding the motif. Next, by computing frequencies across all promoters they concluded that the +1 position has a bias for A or G (together constituting over 80% of the nucleotides) and the +2 position has a bias for T (almost 50%). These frequencies and the −10 motif can be observed in the sequences by eye (Figure 2a) as well as in a logo format (28) (Figure 2c, dotted box). We now show that these nucleotide frequencies are not a general feature of Mtb promoters, but are a consequence of not viewing them as a mixture of diverse promoter-architectures, each with a different distribution of nucleotides and possibly different regulatory function.

#### Mtb promoter-architectures have distinct transcriptional activities

Promoter sequences corresponding to 45 bp upstream and 5 bp downstream of all 1778 promoters were given as input to JAPL. Not surprisingly, JAPL identifies TANNNT as a key feature of the promoters, but it detects a total of nine distinct architectures (Figure 2b and c). The primary distinguishing factor is the position of the motif, which can appear at positions −11 (architecture A), −12 (architecture B, identified as three separate architectures that we name B1, B2 and B3), −13 (architecture C, also separated into C1 and C2) or −14 (architecture D) relative to the TSS. We refer to the distance of the first T of this motif from the TSS as λ.

Cortes *et al*. show a modest correlation between the presence of the TANNNT motif and the expression level of the TSS (Supplementary Figure S2, reproduced from Cortes *et al.*). However, from the architectures identified automatically by JAPL, we note that it is not the presence of the motif, but the spacing between the motif and the TSS that better explains the expression level of the TSS. The TSS expression is significantly higher in architecture A and monotonically decreases as λ increases (Figure 2j).

Although TANNNT is present at position −12 in architectures B1, B2 and B3, they are different in composition: B1 contains an additional weak −35 motif, which was missed in the original MEME analysis. This is not surprising since this architecture contains a total of 162 sequences, less than 10% of the full set. However, its presence is significantly associated with higher TSS expression when compared to TSSs in B2 and B3 (*P*-value <6 × 10^−4^; Supplementary Figure S3), suggesting that the −35 motif may not be vital, but is functional.

Architectures B3 and C1 contain a prominent purine (A/G) at the +1 position followed by TG. A primary result from the Cortes *et al.* CAGE experiments is that a large fraction of Mtb transcripts are leaderless. Since the start codon in Mtb is typically ATG or GTG, these two architectures explain the nucleotide frequencies observed in the original paper at the +1 and +2 locations. Not surprisingly, the fraction of leaderless transcripts is the highest in these two architectures (Figure 2d). Interestingly, the other seven architectures display a close to background level of preference of T at position +2 (27%). The preference of A/G at position +1 in the other architectures also is lower, albeit slightly (77% compared to 84% in B3 and C1). This shows the importance of characterizing the heterogeneity of the promoters explicitly: the overall nucleotide preferences as identified from the full set can be misleading and better explained by decoding the promoter-architectures.

Architectures E and F do not appear to contain TANNNT. But E contains a motif TGNNANNNT, also identified by Cortes *et al.* in their analysis; but these results suggest that there is primarily one preferred location for this motif. F does not contain any obvious motif, but has a prominent C at the TSS, preceded by a weak A/G region.

We note another intriguing pattern that has not been identified before. Architectures C and D, which have a higher value of λ, 13 and 14 respectively, have a strong preference for a pyrimidine (56% C and 35% T) at the position just preceding the TSS. This is not the case in other architectures (37% C and 25% T).

#### Mtb promoter-architecture can change under starvation

We next focused on the 1707 primary TSSs identified in Mtb under starvation conditions. Applying JAPL to this set results in seven architectures (Figure 2e–g), largely similar to those identified in exponential growth, with a few minor differences. Architectures C1 and C2 in exponential growth appear as one C architecture here: JAPL does not separate the leaderless transcripts, while architecture D is missing entirely. This could be a result of a smaller number of promoters reported under starvation as well as a different distribution of architectures across the two environmental conditions. The only other discernible difference is in the architecture that does not have an informative motif at the −10 position. Unlike F in exponential growth, this architecture F’ does not contain a prominent C at the TSS. We believe that F’ may in fact be a mixture of more distinct architectures, but are not identified by JAPL due to a small sample size.

The relationship between the position of the −10 motif and expression pattern of the TSS is nevertheless retained: the TSS expression in architecture A is more than that in B, which in turn is higher than that in C (Figure 2k, Supplementary Figure S4). We asked whether a change of position is also correlated with the change in TSS expression under starvation. Of the 1778 genes expressed in exponential growth conditions, 1512 were also expressed under starvation. Of these genes that were expressed in both conditions, 1314 exponential growth TSSs belonged to an architecture characterized by a λ: A, B, C, or D. Upon starvation, the TSS of 133 of these genes changed by one, two, or three bases such that they could be categorized as having transitioned into an architecture with a different λ. For example, if a TSS of a gene originally in B moved by 1 (or 2) bp downstream under upon starvation, the promoter-architecture is categorized as having changed to C (or D). For each category of transition as well as for TSSs which retained their architecture, the log fold change in TSS expression is plotted in Figure 2l. For genes where the TSS does not change, the TSS expression also does not change significantly (the fold change is close to zero; orange box plots). In contrast, in cases where the architecture changes to one with a larger λ, the TSS expression goes down (red box plots), while in cases where the architecture changes to one with a smaller λ, the TSS expression goes up (green box plots). A gene enrichment analysis (24) identifies only the GO-term ‘growth’ to be significantly enriched (*P*-value ≤ 0.005) in the 133 promoters that underwent a change in architecture. In contrast, no term is enriched in promoters that maintained their promoter-architecture. This suggests that the control of gene-expression may be facilitated by transcribing the gene from a TSS that is either more or less optimal, with a major role played by the spacing between the −10 motif and the TSS.

#### JAPL can be used to predict TSS expression at a new genomic location

We explored whether the two models learned by JAPL had any predictive power in identifying the functionality of a previously unseen genomic position as a potential TSS. Since JAPL is a likelihood based model, any new DNA sequence can be scored using the trained model. We did a genome-wide prediction of TSSs, by computing the likelihood score over each 51 bp window in Mtb using the exponential growth model with nine architectures. Of the 4164 original predictions made by Cortes *et al.*, we removed the 1778 primary TSSs which were used to train the model, to get an unbiased test set of 2386 TSSs. We constructed the standard receiver-operating characteristic (ROC) curve and assessed the predictive power of the model by the area under the ROC curve. For comparison, we also made predictions using a single-architecture model, essentially considering overall distributions at each position from the full 1778 set. The nine architecture model clearly outperforms this simple model (Figure 2h, blue curves). Similarly, in the starvation condition, our test-set consisted of 2426 unseen TSSs. Here too the seven architecture model outperforms a single architecture model (Figure 2h, red curves), illustrating the utility of the models in identifying novel TSSs.

We next explored the extent of the difference between the two trained models themselves. After combining all the identified TSSs across the two conditions and removing primary TSSs which were used for training, we found that 985 TSSs were detected only in exponential growth, 1027 TSSs only during starvation and 1340 TSSs were detected in both conditions. The two trained models were used to compute the log odds score for each TSS, where a higher score suggests expression in exponential growth while a lower score suggests expression during starvation. The log odds scores are significantly different across the three groups (Figure 2i; *P*-value < 10^−12^): TSSs expressed only in exponential growth have a higher score than TSSs expressed in both conditions, which in turn have a higher score than TSSs expressed only during starvation. This is surprising since well over two-thirds of the TSSs are common across the two training sets and the promoter-architectures within the two models are also similar to the eye (Figure 2b and e). This suggests that although the architectures themselves do not vary much, the change in the overall distribution of the architectures has power to discriminate between the two conditions.

#### Promoter-architectures in *E. coli* and *K. pneumoniae*

The relationship between the −10 motif spacing and the expression of the TSS in Mtb is striking and new. To investigate whether this is an Mtb-specific phenomenon, we looked at other bacteria for which genome-wide TSS data are available, namely *E. coli* and *K. pneumoniae* (18). Both species were profiled in mid-exponential phase through modified 5′-RACE followed by sequencing. We used a pipeline similar to Cortes *et al.* to identify 2654 primary TSSs in *E. coli* and 2339 in *K. pneumoniae* from their dataset.

**Figure 3.**
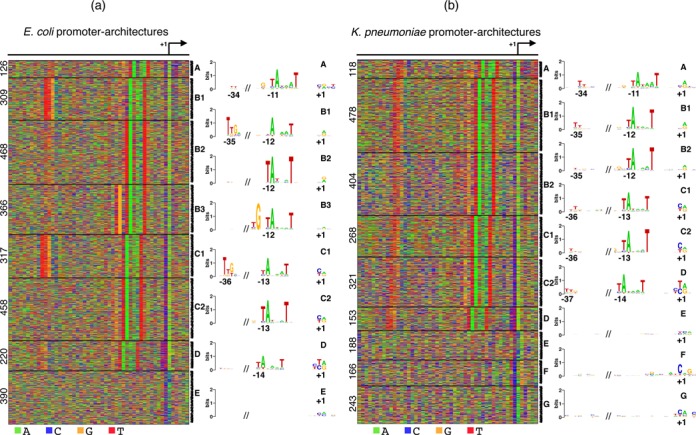
Promoter-architectures in two other bacteria. (a) Eight architectures are identified in *E. coli*, only one of which does not contain the −10 motif. (b) Nine architectures are identified in *K. pneumoniae*; three have no clear −10 motif. Note the presence of a pyrimidine at the −1 position in all architectures where the −10 box is more than 12 bp upstream of the TSS in both bacteria.

Eight architectures are detected in *E. coli* (Figure 3a). Seven of them have variations of the −10 motif TANNNT and as in the case of Mtb, it occurs at positions −11 (A), −12 (B1/B2/B3), −13 (C1/C2), or −14 (D) relative to the TSS. The −35 motif is more prevalent in *E. coli*, as expected (29). However, there appears to be a compensatory effect in the information content of the −35 and −10 motifs: architectures B1 and C1 have a strong −35 motif, but the overall information content of the −10 motif is low. In contrast, at places where the −35 motif is absent (B2 and C2), the −10 motif is stronger. A similar effect is present in cases with the extended −10 motif TGNTANNNT. The presence of the G in the extension compensates for a weaker −10 motif. Both these observations have been noted before in bioinformatic studies targeted towards understanding dependencies within −35, −10 and extended −10 motifs (30). Here we arrive at these conclusions without using any prior knowledge about bacterial binding sites.

Architecture E contains neither of the two motifs and has different nucleotide frequencies at the TSS. The fraction of sequences containing a C at the TSS in all other architectures is low (between 0.02 and 0.1), but in the case of E, it goes up to 0.4. The initiator nucleotide changes from A/G to A/C. This could imply that in the absence of a −10 and −35 motif, *E. coli* uses a different mechanism for transcription initiation.

In the case of *K. pneumoniae*, JAPL identifies nine optimal clusters, six of which contain the −10 motif at positions −11 to −14 (Figure 3b). Interestingly, architectures B and C get split into two each, not based on the presence or absence of the −35 motif (a weak −35 motif appears throughout), but because of the overall background frequencies. In the case of Mtb and *E. coli*, the optimal models had only a few important positions in each architecture. In contrast, in *K. pneumoniae* all nine architectures have different nucleotide frequencies at each position. B2 and C2 have GC-rich promoters (over 50% GC) while B1 and C1 contain more AT-rich promoters (39% GC). Three architectures contain no −10 or −35 motif: these are primarily different at the initiation region and could again imply diverse regulatory mechanisms.

Notably, in both these bacteria, the presence of a pyrimidine is conspicuous at the −1 position in all architectures where the −10 motif starts at −13 or −14. This is similar to our results in Mtb. Also similar to Mtb, all architectures with the −10 motif have a TSS expression significantly higher than those without the motif in both, *E. coli* and *K. pneumoniae* (*P*-value < 10^−16^, Supplementary Figure S5). However, there is no obvious relationship with the TSS expression and the position of the −10 motif. This suggests that although the pyrimidine at the −1 position seems species invariant, the λ-TSS expression association is Mtb-specific.

### Fly

**Figure 4.**
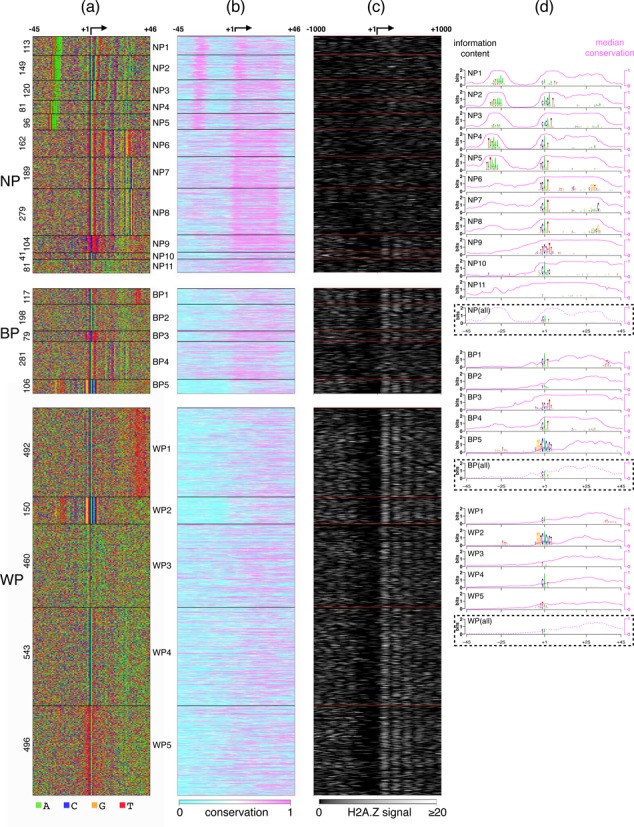
Architectures in three fly promoter classes from PEAT data. (a) A total of 11 architectures are detected in the NP set, five in the BP set and another five in the WP set. (b) Conservation at each position corresponding to sequences in each architecture is plotted. (c) The corresponding H2A.Z nucleosome signal in the 2000 bp window around the TSS in each architecture is shown. (d) The logos (left scale) and the average conservation score (pink, right scale) for each architecture are plotted. The dotted boxes show the average profiles for both quantities for the full NP, BP and WP sets, respectively. While the central three positions −2, −1 and +1 have similar nucleotide distributions across the sets, the component architectures have strikingly different distributions.

Genome-wide experimental identification of TSSs in the fly has been conducted by various groups (8,9,31). We first focused on the high-resolution data reported by (8) using paired-end analysis of TSSs (PEAT) in *Drosophila* embryos. This dataset contains 4339 TSS clusters, classified as narrow peaks, broad peaks and weak peaks based on the mapped read distributions. A narrow peak (NP) is defined as one where the TSS cluster spanned less than 25 nucleotides and the mode of the cluster had more than half the reads mapped to the cluster. A broad peak (BP) is one where the mode still contained over half the mapped reads, but spanned more than 25 nucleotides. All other promoters are classified as weak peaks (WPs). In their analysis, Ni *et al.* aligned all TSS clusters according to the respective modes and evaluated the enrichment of eight known *Drosophila* promoter motifs—Motif1, DRE, TATA, INR, DPE, Motif6, Motif7 and MTE—at various locations along the promoters in the three PEAT sets separately. They found different motifs to be enriched in each set. Furthermore, they showed that the NP set has a stronger preference for a TCA at the −2 position than the BP set, which in turn, had a stronger preference for the 3-mer than the WP set (Figure 4d, dotted boxes). In this section we show that these preferences are not generic to all sequences in the sets, but in fact, each set contains several more intricate patterns, apparent by decoding the hidden architectures.

#### Distinct architectures in PEAT TSSs

We applied JAPL to the NP set containing 1415 TSSs, the BP set containing 781 promoters and the WP set containing 2141 promoters separately. In the NP set, the optimal model contains 11 architectures (Figure 4a). Architectures NP1–NP5 contain the TATA-box, but these are at varying distances from the TSS and more intriguingly are accompanied by different motifs at the TSS (Figure 4d). The INR motif, typically found at the TSS, and in NP4 and NP5, has a consensus of TCAGT (8), but the motifs in NP1, NP2 and NP3 are different. At TSSs in NP1, information content is lower and the consensus is more spread out, while NP2 and NP3 have a CATCAGT and a CACAGT at the TSS, respectively. These motifs look like variants of INR where the first T at −2 is not significant and one (in NP3), two (in NP2) or three (in NP1) nucleotides are inserted between C and AGT of the INR. These variants have not been identified before. A GO-term analysis with DAVID (23) revealed functional terms associated with cuticle formation to be significantly enriched in NP2 (*P*-value < 1.2 × 10^−19^; Supplementary Table S1).

Architectures NP6 to NP8 contain no obvious TATA-box, but contain stronger downstream signals. NP6 contains a match to DPE, but NP7 and NP8 contain novel signals. NP9 contains a TCT motif in place of INR. This motif has been known to be present at the TSS of ribosomal protein genes in many eukaryotes including *Drosophila* (32). Indeed, GO-term analysis reveals several ribosome related terms to be enriched in this architecture (*P*-value < 3 × 10^−90^). NP10 has the smallest number of sequences, which contain a weak variant of the INR motif at the TSS. NP11 does not contain any informative positions.

For the other two sets, BP and WP, JAPL identifies five architectures each. BP1 and WP1, both contain a weak match to INR along with a T-rich region 35-40 bases downstream of the TSS. GO-term analysis reveals that several lumen-related genes are enriched in *both* these sets, suggesting that these architectures may be specific to lumen development. BP5 and WP2 are also similar to each other: they contain the fly promoter element Motif1 (8) at the TSS along with a weak upstream match to Motif6 (8). Both these motifs have been shown to co-occur in previous promoter analyses (13), but here we show that the distance between the two motifs is maintained and the architecture is specific to TSSs with broader distributions.

BP3 is characterized by the TCT motif and like NP2, this group too is enriched for ribosome related genes. At this point we are unable to explain why some of these architectures are enriched for genes with specific functions (Supplementary Table S1). For example, WP5 contains a unique motif at the TSS, not resembling the INR, and this architecture is prevalent among genes that are important for mitochondria.

**Figure 5.**
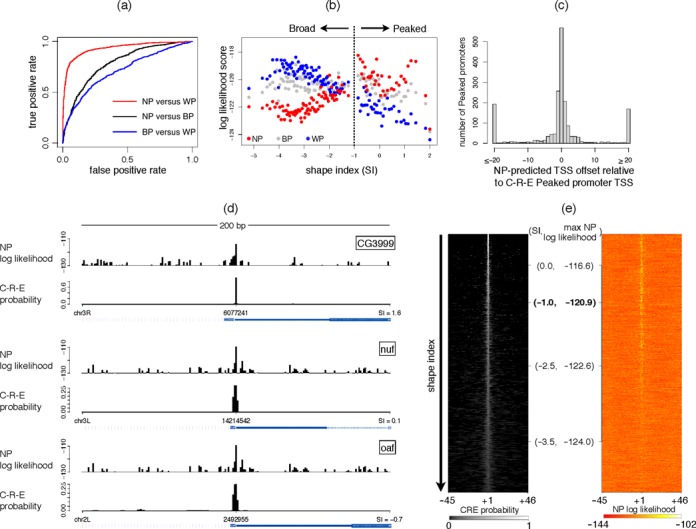
Predictive power of PEAT models. (a) Models from the cross-validation training sets were used to compute log odds scores on respective test sets and ROC curves were used to evaluate three classifiers: NP versus WP (red), NP versus BP (black) and BP versus WP (blue). NP and WP sets are the most separable based on JAPL models. (b) All positions within a C-R-E promoter are tested for being a TSS and the highest likelihood is returned for each of the three PEAT models. The promoters are ordered according to increasing SI on the X-axis and each point corresponds to the average log likelihood score of a bin of 50 promoters according to NP (red), BP (grey) and WP (blue). NP log likelihoods are positively correlated with the SI, while WP log likelihoods are negatively correlated with the SI (*P*-value < 10^−16^ for both). The BP log likelihoods have a weak negative correlation with the SI (*P*-value < 10^−4^). (c) A histogram of the relative offset of a prediction of the TSS in the Peaked C-R-E promoters by the PEAT NP model with respect to the reported mode of the distribution is shown. (d) The C-R-E TSS probability distribution and the NP model predictions are plotted for the three Peaked promoters used by Hoskins *et al.* for illustration purposes in their paper. (e) The C-R-E TSS probability distributions over all promoters not in the PEAT dataset are ordered according to their SI on the left. The NP model log likelihoods of TSSs over the corresponding regions are shown on the right. The position of the demarcator SI of −1 for Peaked promoters is shown in bold.

To measure the predictive power of the models to identify novel TSSs in the same class, we used the log odds scores to distinguish between NP and WP classes, NP and BP classes, and BP and WP classes (Figure 5a: red, black and blue curves, respectively). The testing was done using 5-fold cross-validation ensuring that no promoter that was used for training was also used for validation of the same model. The ROC curves show that NP and WP promoters are more different from each other (AUC = 0.93) than are NP and BP (AUC = 0.79) or BP and WP (AUC = 0.71). This is also apparent from the similarities in some of the architectures across the three models.

#### Architectures have distinctive conservation and chromatin features

We used the phastCons scores (19) computed for the fly genome using 14 related insects to study evolutionary profiles of the architectures (Figure 4b). The score at each position lies between 0 (least conserved) and 1 (most conserved). In most architectures we note that high information content relates to higher conservation (Figure 4d). This relationship fails in the case of BP5 and WP2. Intriguingly, sequence conservation at Motif1 at the TSS and at the upstream Motif6 is distinctly lower than analogous positions in other architectures. This architecture appears to be *Drosophila*-specific.

The dotted boxes in Figure 4d show the average information content and the conservation across each set, without separating the architectures. Three regions in the NP set are conserved: −35 to −20, −5 to 10 and 25 to 35. But when the architectures are decoded, a compensatory effect between the upstream and the downstream components becomes apparent, both, in terms of information content and sequence conservation.

Rach *et al.* (20) showed that nucleosomes, specifically the H2A.Z variant, displayed varying patterns across the three sets. We therefore analyzed embryonic H2A.Z nucleosome occupancy data from (21) in the 2000 bp window around each promoter. Broadly consistent with results of Rach *et al.*, the WP set has more well-positioned H2A.Z nucleosomes downstream of the TSS than the BP set, which in turn has more well-positioned H2A.Z nucleosomes than the NP set (Figure 4c). However, this is not true for all architectures within the three sets. NP10 and the TCT-containing NP9 possess well-positioned nucleosomes, while BP4 clearly lacks them. Promoters with the TCT motif have been shown earlier to display these characteristics (20).

Looking at the similarities across promoters within the NP & BP and BP & WP sets, we asked whether applying JAPL to the combined set can be better at separating the three sets. JAPL identifies 12 architectures fly1 to fly12 in the full set of 4337 promoters (Supplementary Figure S6). All promoter elements that were identified in the separate sets, such as the Motif 1, variations of INR, T-rich regions, etc. are detected in the full set as well. Architectures fly1 to fly7 are dominated by NP promoters, while fly8 to fly12 mostly contain WP promoters. Not surprisingly, architectures that are similar across the sets in Figure 4a, get identified as one here. These results, combined with the sequence conservation and nucleosome occupancy, suggest existence of two distinct sets rather than three. Interestingly, over a fifth of the promoters contributing to the four TATA-box-containing architectures (fly1 to fly4) are from the WP set. This shows that having a TATA-box does not always imply a narrow peak, at least as defined by Ni *et al.* However, neither did the TATA-box come up as overrepresented in the WP set in their analysis (8), nor is any TATA-box containing architecture detected by JAPL in WP (Figure 4). This is because these TATA-box containing promoters make up less than a tenth of the complete WP set and sequences in each of the four architectures separately make up even less than that. Taken together, this further supports our rationale of explicitly identifying architectures based on sequence features instead of doing average analyses across all promoters, or those divided into classes based on a single regulatory feature.

#### TSS distribution can be predicted accurately at unseen genomic locations

As part of the modENCODE project, fly TSSs have also been mapped separately using CAGE and 5′ RLM-RACE (9). The authors have further produced an integrated map of TSS distribution from these two methods combined with RIKEN embryo EST data (31). This map, which we will henceforth refer to as the C-R-E data, contains 12 454 promoters across 8037 genes. Over each C-R-E promoter, Hoskins *et al.* (9) computed a probabilistic distribution of each nucleotide being a TSS, based on which they defined a shape index (SI) per promoter. A higher SI implies focused transcription, with most of the transcription occurring from one or two positions, while a lower SI implies dispersed transcription with a broad TSS distribution. Hoskins *et al.* used a cut off of −1 to demarcate the boundary between “Peaked” (2382) and “Broad” (6603) promoters. The remaining 3469 promoters were not classified as either, due to low tag count or class-instability.

We used the three models learned from the PEAT dataset on the C-R-E Peaked and Broad promoters. In order to assess the models’ sensitivity in locating new promoters, we removed the 3204 regions that intersected with any promoter in the PEAT set. This left us with 1886 Peaked and 3895 Broad C-R-E promoters. We used the NP, BP and WP models separately on all these regions by evaluating the likelihood of every position within the region for being a TSS. The highest log likelihood value over a promoter was assumed to be its score. The three scores—NP, BP and WP—correlate differently with the shape index (Figure 5b). The WP definition of the PEAT data is more similar to the the Broad peaks definition in the C-R-E set, while the NP is more similar to the Peaked promoters. This is interesting for two reasons. First, this set contains no promoter on which any of the NP/BP/WP models were trained. These results are therefore on an entirely unseen dataset, illustrating the predictive power of the models. Second, the definition of a peaked promoter in the PEAT set differs from the shape index associated with the C-R-E set.

For all C-R-E promoters with SI >−1, we computed the difference in the predicted TSS based on the NP model and the mode of the C-R-E probability distribution (Figure 5c). For over 60% of the promoters this difference is within two nucleotides. Interestingly, the Hoskins *et al.* report that TSSs predicted by the three methods in the data—CAGE, RLM-RACE and EST—are also within 2 nucleotides of one another. Figure 5d shows the C-R-E probability distribution over a 200 bp neighborhood of three Peaked promoters used for illustration purposes by Hoskins *et al*. The log likelihoods from the NP model across the regions are unambiguously the highest at the C-R-E TSS. This trend persists in high SI promoters and diminishes as the SI reduces (Figure 5e).

### Human

The FANTOM5 database (33) contains CAGE tags from 517 human samples. After pooling these tags, Frith *et al.* (10) identified 17 039 promoters which can be attributed to a RefSeq gene (34) within 50 nucleotides. From tag distributions along a 100 bp window the authors computed the interquantile range (IQR), which is a measure of the TSS spread. From the tags arising from individual cell-types, they computed the entropy, which is a measure of cell-type specificity for each promoter. From the sequence of the promoters, they computed CpG islands and the TATA-boxes in each promoter as well. They concluded that many of these features are correlated. We used −45 bp upstream and +45 bp downstream of all identified TSSs with at least 100 tags. Upon removing regions having repetitive elements (19), we were left with 12 475 TSSs associated with some RefSeq gene. JAPL was applied to the full set.

#### Human promoters have far more overall heterogeneity

**Figure 6.**
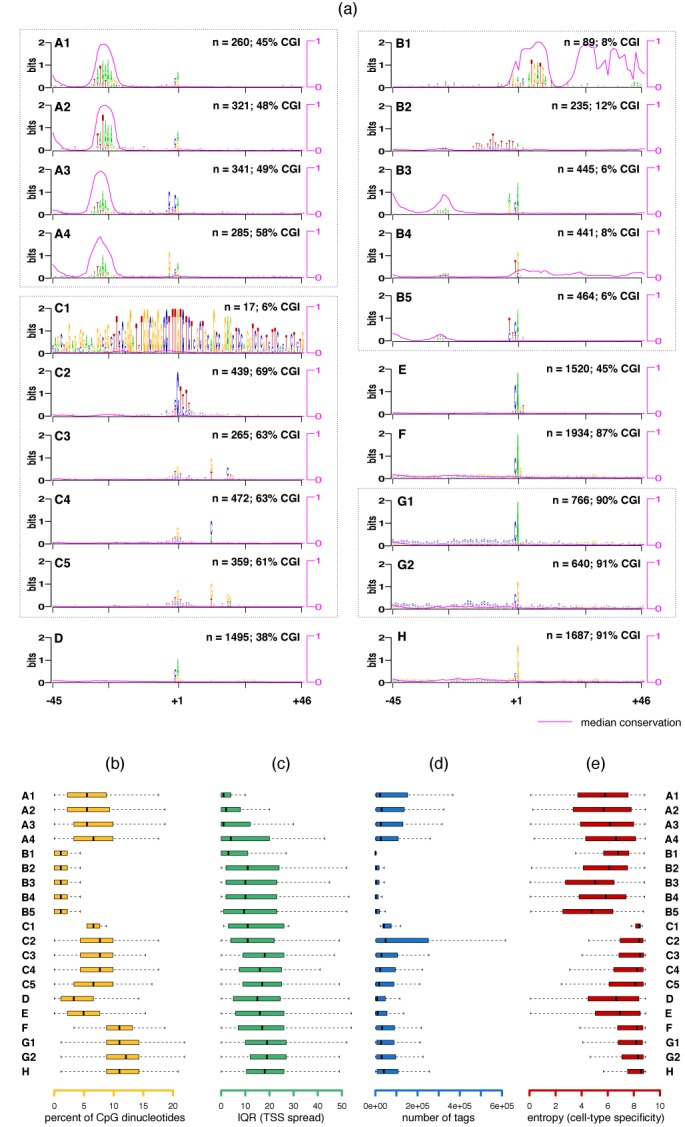
Twenty promoter-architectures in the human genome and their characteristics. JAPL was run on the eight original architectures detected by JAPL (Supplementary Figure S7). (a) Architecture A was further split into four architectures based on the position of the TATA-box and sequence variations at the TSS. Architecture B was split into five architectures based primarily on variations near the TSS. Architecture C was also split into five architectures, based on the TCT motif at the TSS and downstream nucleotide variations. Architectures D, E, F and H were not split further. Architecture G was split into two architectures based on dependencies within nucleotides at the −1 and +1 position. The median conservation score for each architecture is plotted on top of the logos in pink. The two quantities on each logo indicate the number (*n*) of TSSs contributing to the architecture and the percentage of TSS neighborhoods falling in an annotated CpG island. (b–d) Boxplots of four different features normally associated with regulatory functions of promoters are displayed for each architecture: overall CpG dinucleotide content, IQR (high IQR → large spread), total tags mapping to the TSS neighborhoods across experiments and entropy (high entropy → low cell-type specificity).

JAPL identified eight architectures, while selecting all 91 positions as relevant (Supplementary Figure S7). Architectures A and B have a TATA-box, C has a pyrimidine-rich region at the TSS, while all other architectures D–H have variants of pyrimidine followed by a purine at the TSS. Now, JAPL, when identifying important positions, exploits that notion that the frequency distribution of each non-important position is invariable across *all* architectures for which the same position is also non-important. However, the eight architectures have very different frequency distributions over all, evident from the fact that all positions are selected as important. We therefore investigated the possibility of each of these architectures being further composed of multiple distinct architectures, with a few key differing informative positions. So JAPL was applied separately to each of the eight architectures. For four of them (D, E, F and H), the selection method did not detect more than one architecture, implying lack of heterogeneity within them. But the remaining four architectures, A, B, C and G, were further separated into four, five, five and two architectures, respectively (Figure 6a, Supplementary Figure S8).

#### Identified architectures are associated with distinct functional features

Architectures A1–A4 have similar CpG content (Figure 6a and b) and are characterized by a TATA-box ∼30 bases upstream of the TSS. In all four architectures, the TATA-box location is conserved across vertebrates (Figure 6a, pink curves). The differences across the architectures are subtle, but significant. The TATA-box in A2 is shifted by one base upstream relative to A1. A3 and A4 have a weak TATA-box and variations near the TSS. The −3, −2 and −1 positions in A3 have a preference for a pyrimidine: 99.6%, 84% and 94%, respectively, which reduces dramatically in A4 to 0%, 44.9% and 81.2%, respectively. While this may be the only difference between the promoters in terms of sequence, the TSS spread across these promoters is also different. The IQR values, where a low value implies more focused transcription, are significantly different (*P*-value < 5 × 10^−4^): the string of pyrimidines may play a role in ensuring a smaller spread of transcription (Figure 6c). Overall, though, all four A architectures have a lower TSS spread with respect to other architectures.

Architectures B1–B5 also have an AT-rich region 30 bp upstream of the TSS, which resemble weak TATA-boxes or TATA-boxes at variable distances from the TSS. B1 is interesting due to its highly informative and conserved downstream region. Over 60% of the genes in this set are in fact small nucleolar RNAs, of which the ATGATG is a strong structural component (35). B2 contains a 15 bp stretch of pyrimidines just upstream of the TSS. A TATA-box followed by a region of pyrimidines with a dominating T, like in B2, has been identified and shown to play a regulatory role in a few genes in yeast (36,37). But as per our knowledge, this feature has not been detected in the human genome at this scale. (38) showed that TATA-binding proteins can bind poly-T tracts and that a small fraction of human core promoters contain a TATA-box followed by a T-rich region upstream of the TSS.

Architectures B3 and B5 have the same variations at the TSS as those in A3 and A4. The three nucleotide stretch of pyrimidines just upstream of the TSS in conjunction with the TATA-box is prevalent in A3 and B5. The preference for a pyrimidine, purine at −1, +1 at human TSSs is known (39). However, from the preferences at these locations in B3–B5, we note that a TG or CA is more prevalent: whenever a C appears at −1, A appears 85% of the time at +1 and whenever a G appears at +1, it is preceded by a T almost 70% of the time.

The strength of the TATA-box is not the only difference between architectures B2–B5 and A1–A4. First, TSS neighborhoods in B2–B5 have far fewer total tags than any other architectures (Figure 6d). Second, these architectures have a higher TSS spread (Figure 6c). This may be attributed to a weaker TATA-binding signal. Third, they have more cell-type specific expression patterns (Figure 6e). Finally and most strikingly, these sequences are significantly depleted in CpG dinucleotides (Figure 6a) and in C and G in general. This implies that the TATA-box alone should not be considered when exploring functionalities of the promoter, instead its existence needs to be treated in conjunction with other features. Results of architectures A and B together suggest that a strong TATA-box within a CpG-rich promoter functions differently in terms of spread and cell-type specificity than a weak TATA-box within an AT-rich promoter.

Architectures C1–C5 have more CpG dinucleotides than A and B, and lack the TATA-box. C1 has only 17 promoters, with high information content: they all belong to the zinc finger family of proteins. 13 of these proteins occur on chromosome 19 and are possibly a result of gene-duplication (40).

Architecture C2 contains the TCT motif and as in the case of *Drosophila*, a GO-term analysis reveals enrichment of ribosome related functions (*P*-value < 10^−40^). It also contains genes with the most number of tags (Figure 6d), possibly since these genes are required in large numbers. Architectures C3, C4 and C5 contain a variant of the TCT motif with the C at the +1 position replaced by a C/G. But more strikingly, the downstream sequence characteristics in these architectures are different. C3 and C5 contain a G/T at position +13, while C4 contains a C/A. This is not a spurious characterization, since the +19 region also differs across the three architectures. In C3, there is a preference for a CTG, which is known to be part of the DCE motif in eukaryotes (41). We are not aware of the function of the downstream motif in C5.

Architecture D contains fewer CpG dinucleotides and a weak stretch of purines between the +23 to +30 region. Architectures E, F and G1 vary in CpG content, but contain a strong A at the TSS, preceded by a slightly weaker C. Architectures G2 and H, on the other hand, contain a G at the TSS, preceded by a much weaker C. This difference in the probability of C at the −1 position could result from the CpG methylation present at −1 and the subsequent mutation of a C to a T. If the C on the positive strand methylates and mutates, this will result in a G at +1 preceded by a T. In contrast, if the C on the negative strand gets mutated to a T, this will result in an A at the TSS. The strong C preceding a A at TSS and a weak C preceding the G at TSS provides support to this conjecture. Finally, although architectures G and H have similar CpG frequencies (Figure 6b), they differ in C and G proportions: G is more C-rich (42% C, 31% G) while H is more G-rich (29% C, 42% G). Although they have fairly similar IQR values, number of tags and entropy values, genes in H are enriched for being part of the lumen (*P*-value < 10^−8^), suggesting that G-rich promoter elements may be important for that function. The GO-term analysis for all architectures are available in Supplementary Table S2.

As in the case of fly promoters, we analyzed the nucleosome structure around human promoters. We used the nucleosome signal data from ENCODE for the GM12878 cells (22). Generally consistent with results from (20), the promoter-architectures associated with a large TSS spread: C, F, G1, G2 and H have well-positioned nucleosomes downstream of the TSS (Supplementary Figure S8). However, architectures D and E, which have similar IQR values (Figure 6c), do not display the strong downstream nucleosome organization. The lower CpG-content in these architectures may play a role in this apparent lack of chromatin structure. Furthermore, the expression patterns of the promoters within these two architectures show somewhat higher cell-type-specificity (Figure 6e), suggesting the presence of architectures with multiple distinguishing features: no TATA-box, low CpG-content, fewer well-positioned downstream nucleosomes, low expression breadth across cell-types, but TSS spread that is similar to other TATA-less promoters.

Interestingly, although both A and B have a TATA-box upstream of the TSS, the nucleosome positioning as with the other characteristics discussed earlier is different across the two sets. Architecture A has a nucleosome free region just upstream of the TSS, which is lacking in architecture B.

The TSS data were pooled from several different cell-types, so no nucleosome data from a single cell-type are technically appropriate here. But assuming that human DNA encodes high levels of nucleosome occupancy (42), we use these data as an approximate indicator to chromatin structure at most promoters. Moreover, the overall results do not change when we use another cell-type (Supplementary Figure S9).

## DISCUSSION

We have introduced a new organism-independent method for identifying promoter-architectures. This method learns important promoter elements automatically from the data without getting biased to what is known in the literature. We detected novel architectures in all organisms on which JAPL was applied. For example, although bacterial promoters have been studied for decades, the relationship between the pyrimidine at the −1 position and the position of the −10 motif has not been noted before. It is possible that the presence of the pyrimidine ensures that transcription does not occur from the −1 or −2 position, since most bacteria favor a purine (A/G) at the TSS (27). This would explain why the pyrimidine occurs significantly only in those architectures where the spacing between the −10 motif and the TSS is larger than the canonical distance (43), i.e. first T in the TANNNT is at position −12 with respect to the TSS. The same correlation is apparent in TSS data from pathogens Salmonella (44) and Helicobacter (26) (not discussed in detail, but shown in Supplementary Figure S10) and we therefore suspect that this is a widespread bacterial phenomenon. Interestingly, only in Mtb, the spacing between the −10 motif and the TSS is correlated with the expression of the downstream gene. The larger the spacing, the lower is the transcriptional activity. Since transcription in Mtb can begin from any nucleotide (27), we hypothesize that when subjected to environmental stress, this spacing may be key towards modulating gene expression.

In *Drosophila*, the method identified several new variants of the INR element, which are correlated with the position of the upstream TATA-box. We found a new element that may be associated with cuticle development. We showed that although chromatin characteristics correlate with the TSS spread, some architectures such as those containing the TCT-motif can dilute the overall correlation and need to be studied separately. Similarly, although promoters with a narrow peak have higher sequence conservation, the upstream and downstream conservation is compensatory in nature. Only after unraveling the individual architectures can these properties of promoters be understood. In human, we find 20 different promoter-architectures accounting for over 12 000 genes. From the full set of TSSs the method was able to identify small clusters of zinc finger proteins and small nucleolar RNAs automatically. We also show the importance of using all the data without any prior partitioning based on features like the TSS spread. Since the measures for the TSS spread can change based on the person defining it: from the use a Gaussian distribution (8) to the interquantile range (10) to the entropy of the TSS distribution (9), we suggest identifying architectures based on sequence features first, and then focus on understanding how those architectures might affect regulation. Our analysis shows that human promoters have a lot more heterogeneity in terms of sequence, cell-type specificity, TSS spread and chromatin structure. It is not sufficient to divide them solely on the basis of the TATA-box, CpG islands, expression breadth or TSS distribution to understand their regulatory mechanisms. While many of these characteristics are correlated, as evident from this and earlier analysis (10), the correlations are far more complex, with no single characteristic sufficient to dictate the behavior of the transcription initiation.

Most importantly, we present a new way of analyzing TSS data. Methods developed so far can be categorized into three groups. The first kind assess enrichment of known promoter elements such as TATA-box, INR, DPE, GC-richness etc. in the TSS neighborhood (45,46) and estimate various classes of promoters (47,48). Their results depend directly on elements considered and are therefore organism-specific. The second kind use a two-step approach: apply de novo motif discovery first and then build a model on top of enriched motifs or words (13,49). Their efficacy depends on the accuracy of both steps. Since the first step identifies motifs that are overrepresented across the whole set, motifs that are present specifically only in a few promoters cannot be detected. For example, we were unable to find any study that identifies the TCT motif pertaining to ribosomal genes from large-scale studies, without prior knowledge, since it is present in a small set of TSSs. The third kind use sequence characteristics of the promoter set and a control set to learn features specific to the former. The primary aim of these methods is to distinguish between promoters and non-promoters. A wide range of modeling techniques have been used for this purpose: position specific Markov models (50), linear regression (51), decision trees (52), artificial neural networks (53) and support vector machines (54).

JAPL does not fall into any of these categories. The goal of this work is not to produce a black-box classifier that can predict whether a region along a genome has potential for being a promoter or a TSS. Indeed, we believe that newer, inexpensive and fast high-throughput experiments do exactly that. Instead, our aim is to understand the mechanism behind each TSS getting reported in these experiments. Our approach explicitly unravels each possible promoter-architecture. Furthermore, it adopts a format akin to the widely popular position weight matrices (55) used to represent protein-DNA binding sites. The resulting architectures therefore directly provide insights into regulatory mechanisms behind the expression of each TSS. That said, we have demonstrated that the likelihood-based nature of the models make them amenable to be used as classifiers to distinguish between promoters and non-promoters (Figure 2h), or promoters in two different cell-types (Figure 2i), or promoters with two different characteristics like broad peaks and narrow peaks (Figure 5a). Finally, the generative model formulation of the promoter-architectures has implications in synthetic promoter biology. The positions identified as important can potentially be used to create promoters having regulatory characteristics pertaining to that architecture.

JAPL by itself can be considered as an unsupervised learning/clustering method with feature selection. Machine learning methods that perform feature selection can be broadly categorized as filter, wrapper and embedded methods. Filter methods preprocess the data to identify key features before learning the clusters; wrapper methods search through the space of features and after learning the clusters on various subsets, select the best set of features; while embedded methods lie in between, with feature selection being part of the objective function (56). Wrapper methods, the category in which JAPL belongs, are typically more accurate that the other two, but are slower in speed. We have built on the model developed by Vaithyanathan and Dom (57) to cluster documents, where they use a probabilistic representation of the problem and learn a global subset of useful features across all clusters. We relax this condition and instead, allow each architecture to have its own distinct set of useful positions. Although this increases the complexity of the model, such a model is more suited to our problem where certain positions may be important only for a few specific architectures.

The model can be further improved in three ways. First, the cross-validation approach towards learning the best model involves learning several models by varying the number of architectures *k* and the number of important positions. Indeed, the number of combinations grows exponentially with *k* (Supplementary Figure S11). The current algorithm can deal with models of at most 15 architectures and few possibilities of important positions. However, for larger datasets and more complex models, a more efficient optimization technique will be beneficial. Second, JAPL currently assumes a common background across all architectures for an unimportant position. This works for lower organisms like bacteria and fly, but not for the human genome: eight architectures with different distributions at *all* positions are identified and then, within four of those, JAPL identifies further heterogeneity. This is due to the large local sequence heterogeneity within mammalian promoters such as GC-rich promoters, promoters within CpG islands, AT-rich promoters, and of course, promoters that do not fit in any of these categories. Relaxing the common background assumption might help: instead, the background can be common across a set of architectures, which will be another parameter to be learned. Third, here we report only the top scoring model. However, there is usually a small set of models that score higher than others in the datasets (Supplementary Figure S12). These top-scoring models are typically similar in nature, but differ slightly in the number of architectures: a few architectures get merged or one gets split. Harvesting information from the ensemble of such high-scoring models instead of relying on the sole optimal solution has been shown to perform better in de novo motif discovery (58) and in RNA structure prediction (59). Since JAPL anyway learns all the models first, this route is worth exploring.

Until recently it was not possible to unravel heterogeneity at this level. Now, however, we have a lot more genomic data than ever before. This is true not only for TSS identification, but for experiments that probe other biochemical processes as well, such as protein-DNA interactions, chromatin organization, RNA levels, splicing events, etc. Regions reported from any such experiment can be a result of various different biological mechanisms. For example, a splicing event may be the result of a splicesosome, or due to self splicing. Similarly, a region may be nucleosome free because it is an enhancer, or a silencer, or near an actively transcribed gene, or a poised gene, or even because it was recently replicated. Indeed, there is inherent mechanistic heterogeneity in such data and relatively few attempts to characterize it in an unbiased manner. We have recently shown how a protein can bind various parts of the genome in strikingly different ways (directly, indirectly, or as a complex) in the same cell-type and how this can be detected from a single high-throughput chromatin immunoprecipitation experiment targeting that protein (60). Kundaje *et al.* (61) have shown the existence of heterogeneity within chromatin patterns around protein-DNA binding sites. They highlight the need to go beyond analyzing aggregate or average chromatin signals around specific genomic features. We believe this is the first general method that can identify heterogeneity in terms of nucleotide composition. Since the method is not promoter-specific, in principle, it can be applied to large-scale data arising from any specific genomic events reported at high resolution.

## SUPPLEMENTARY DATA

Supplementary Data are available at NAR Online.

SUPPLEMENTARY DATA
