# Supplementary material for: Multiple novel promoter-architectures revealed by decoding the hidden heterogeneity within the genome
Source: Nucleic Acids Res. 2014 Oct 17;42(20):12388–403. doi: 10.1093/nar/gku924 (PMC4227772; doi:10.1093/nar/gku924)
Supplement: SUPPLEMENTARY DATA [file supp_42_20_12388__index.html]

Multiple novel promoter-architectures revealed by decoding the hidden heterogeneity within the genome — SUPPLEMENTARY DATA 

# Multiple novel promoter-architectures revealed by decoding the hidden heterogeneity within the genome

## SUPPLEMENTARY DATA

**Files in this Data Supplement:**

- SUPPLEMENTARY DATA
- SUPPLEMENTARY DATA
