# Supplementary material for: Multiple novel promoter-architectures revealed by decoding the hidden heterogeneity within the genome
Source: Nucleic Acids Res. 2014 Oct 17;42(20):12388–403. doi: 10.1093/nar/gku924 (PMC4227772; doi:10.1093/nar/gku924)
Supplement: SUPPLEMENTARY DATA [file supp_gku924_nar-02085-n-2014-File007.pdf]

SUPPLEMENTARY INFORMATION

Figure S1. Results of JAPL on two simulated datasets. (a) The plot shows the average ARI per cross-validation fold versus the corresponding mean likelihood score on the test set for the simulated dataset with five architectures. Different values of k are indicated by different colors and the numbers of important features are indicated by different shapes. To assess importance of the correct number of important positions, each architecture is forced to have the same number of important positions varying from 5 to 100 although the data was simulated with at most 10 important positions. The highest ARI and likelihood is achieved for $k = 5$ and 10 important positions. The model tolerates over-estimation of the number of important positions to a certain degree, but wrongly estimating the number of clusters is more costly in terms of the ARI. (b) The plot shows the mean likelihood score across each cross-validation test fold for the two simulated datasets with a single architecture. As k increases, the likelihood decreases, illustrating that the model selection method correctly identifies lack of heterogeneity. Since there is only one architecture, the ARI is undefined and the number of important features is trivially all, by design of the model.

Figure S2. TSS expression across Mtb architectures compared to ?). (a) Figure **??**e from the main text has been reproduced to compare with (b) Figure 1C from **?**). TSS peak heights are more separable across architectures detected by JAPL than those characterized purely on the basis of presence or absence of motifs. Note the difference in the scales: no boxplot in the **?**) figure has a median over 2. In contrast, TSSs in architecture A have a much taller peak, with a median close to 4.

Figure S3. TSS expression across all individual architectures in exponentially growing Mtb.

Figure S4. TSS expression across all individual architectures in Mtb after starvation.

Figure S5. TSS expression levels of identified architectures in *E. coli* and *K. pneumoniae*. Boxplots of the number of reads associated with TSSs in each architecture identified in (a) *E. coli* and (b) *K. pneumoniae* show no relationship between λ and the TSS expression.

Figure S6. Results of JAPL on all PEAT sequences together. (a) JAPL identifies 12 architectures on the combined dataset of NP, BP, and WP. The architectures are similar to those identified separately. The corresponding (b) sequence conservation and (c) nucleosome occupancy for the architectures are consistent with those identified in the separate sets. (d) The fraction of promoters in each architecture originally identified as NP (yellow), BP (white), and WP (red) are shown. While no architecture contains only one type of promoter as defined by **?**), most are largely dominated by NP or WP.

Figure S7. Eight primarily architectures learned on human data. Model selection identified the model that used eight architectures and considered all 91 positions as important. Indeed, the

differences across architectures are evident at all positions.

Figure S8. Nucleosome occupancy across human promoter-architectures. The bulk nucleosome signal in GM12878 cells in the 2000 bp window around the TSS in each architecture is shown.

Figure S9. Nucleosome occupancy across human promoter-architectures. The bulk nucleosome signal in K562 cells in the 2000 bp window around the TSS in each architecture is shown.

Figure S10. Pyrimidine at -1 position in human pathogens. Architectures identified in (a) Salmonella and (b) Helicobacter indicate the enrichment of a pyrimidine before the transcription initiation site in cases where the -10 motif is further upstream.

Figure S11. Time taken to learn models on the simulated dataset. The total time taken to learn all models with number of important positions drawn from $\{5, 10, 100\}$ with respect to the number of architectures in the model is shown in blue. The number of models increase exponentially with k. The average time for a model is shown in red. All times are for a serially running code on a single core of an AMD processor. In practice, all models are run on a computational cluster simultaneously as separate jobs.

Figure S12. Scores of top 50 models learned on real datasets. The set of numbers at the top left corner of each plot denotes the number of important positions in each respective architecture for the top scoring model. This is the model that is used in all analyses done in the main text. The total number of positions, that is, the size of promoters are denoted in the bottom right corner. In most plots there is a set of models that score higher than others.

Table S1. GO-term analysis on all identified fly architectures.

Table S2. GO-term analysis on all identified human architectures.

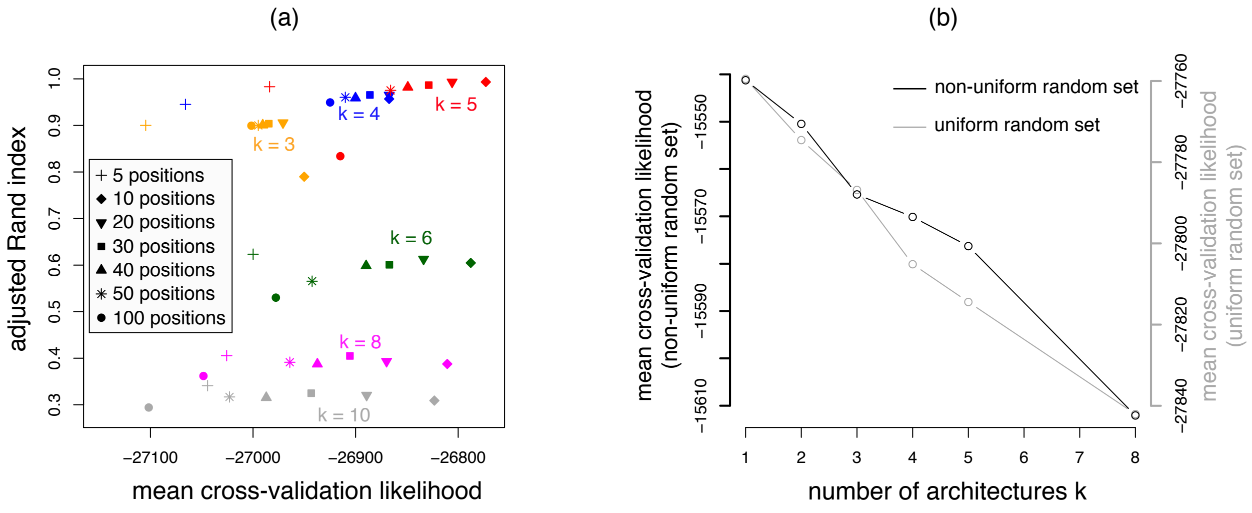
(a)
(b)
adjusted Rand index
mean cross-validation likelihood
5 positions
10 positions
20 positions
30 positions
40 positions
50 positions
100 positions
k = 3
k = 4
k = 5
k = 6
k = 8
k = 10
mean cross-validation likelihood (non-uniform random set)
mean cross-validation likelihood (uniform random set)
non-uniform random set
uniform random set
number of architectures k

Figure S1

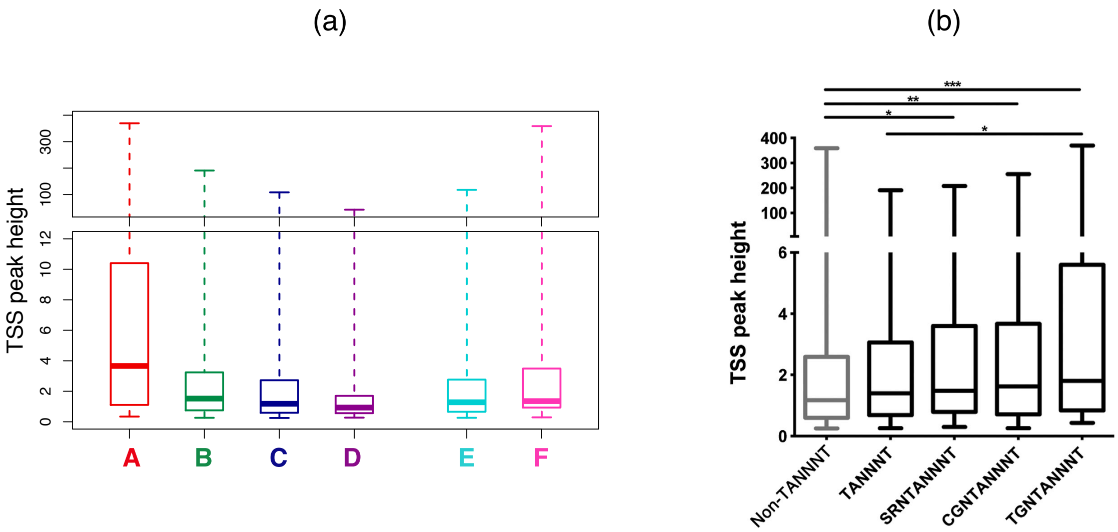
(a)
(b)
TSS peak height
300
100
12
10
8
6
4
2
0
A
B
C
D
E
F
***
**
*
*
400
300
200
100
6
4
2
0
Non-TANNNT
TANNNT
SRNTANNNT
CGNTANNNT
TGNTANNNT

Figure S2

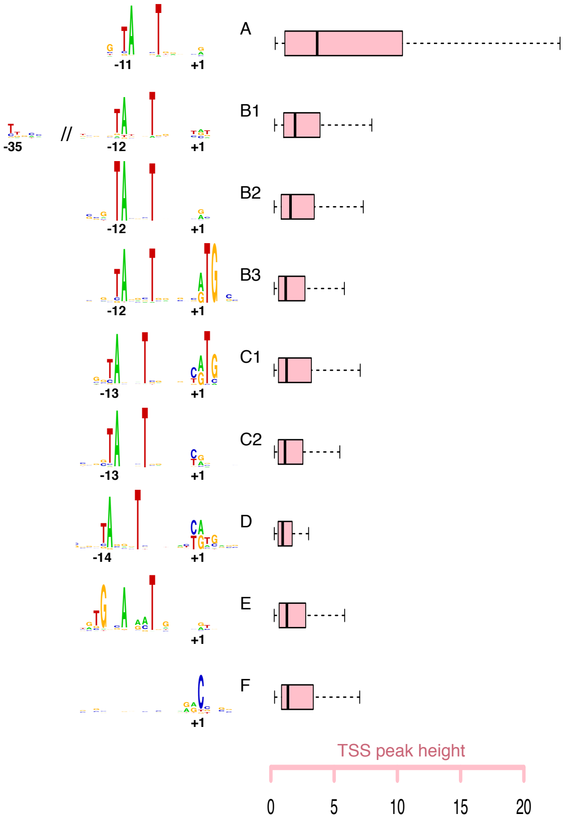

-11
+1
A
-35
//
-12
+1
B1
-12
+1
B2
-12
+1
B3
-13
+1
C1
-13
+1
C2
-14
+1
D
+1
E
+1
F
TSS peak height
0
5
10
15
20

Figure S3

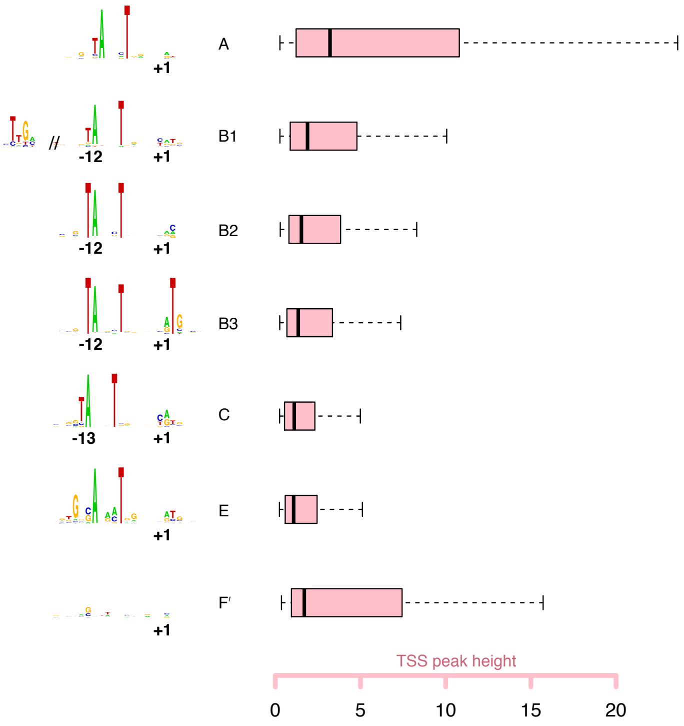

Figure S4

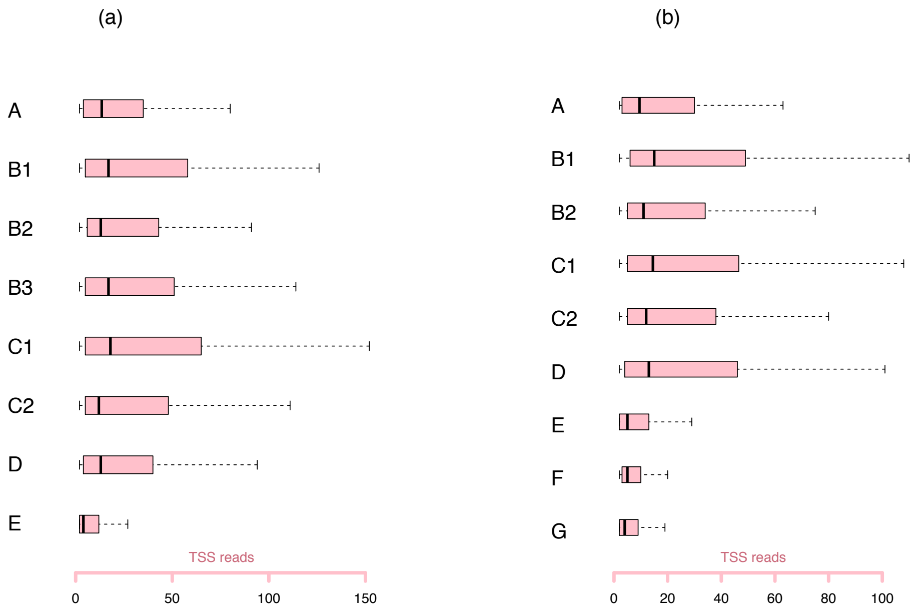

(a)
A
B1
B2
B3
C1
C2
D
E
TSS reads
0
50
100
150
(b)
A
B1
B2
C1
C2
D
E
F
G
TSS reads
0
20
40
60
80
100

Figure S5

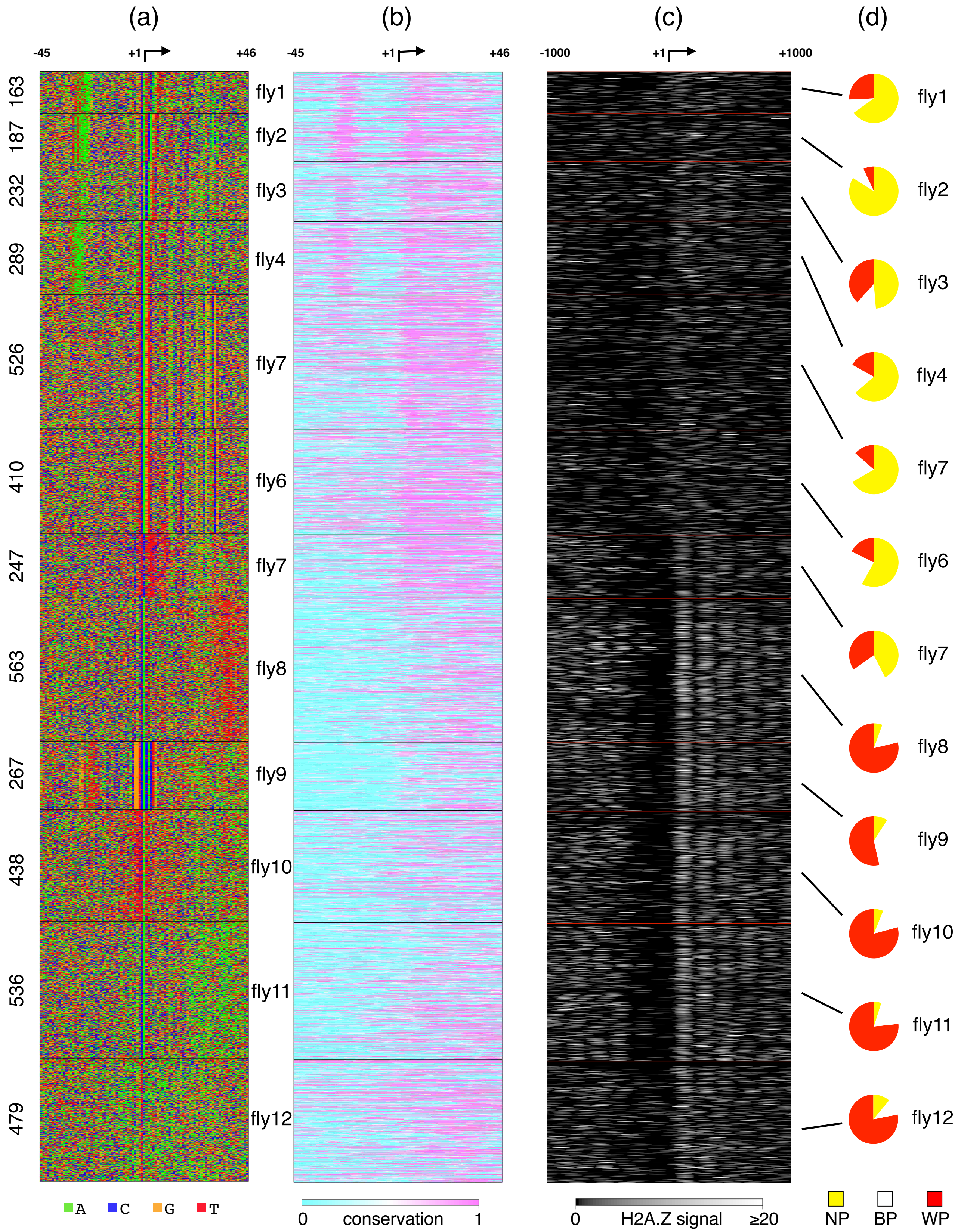
(a)
(b)
(c)
(d)
-45
+1
+46
-45
+1
+46
-1000
+1
+1000
163
187
232
289
526
410
247
563
267
438
536
479
fly1
fly2
fly3
fly4
fly7
fly6
fly7
fly8
fly9
fly10
fly11
fly12
fly1
fly2
fly3
fly4
fly7
fly6
fly7
fly8
fly9
fly10
fly11
fly12
A
C
G
T
0
conservation
1
0
H2A.Z signal
≥20
NP
BP
WP

Figure S6

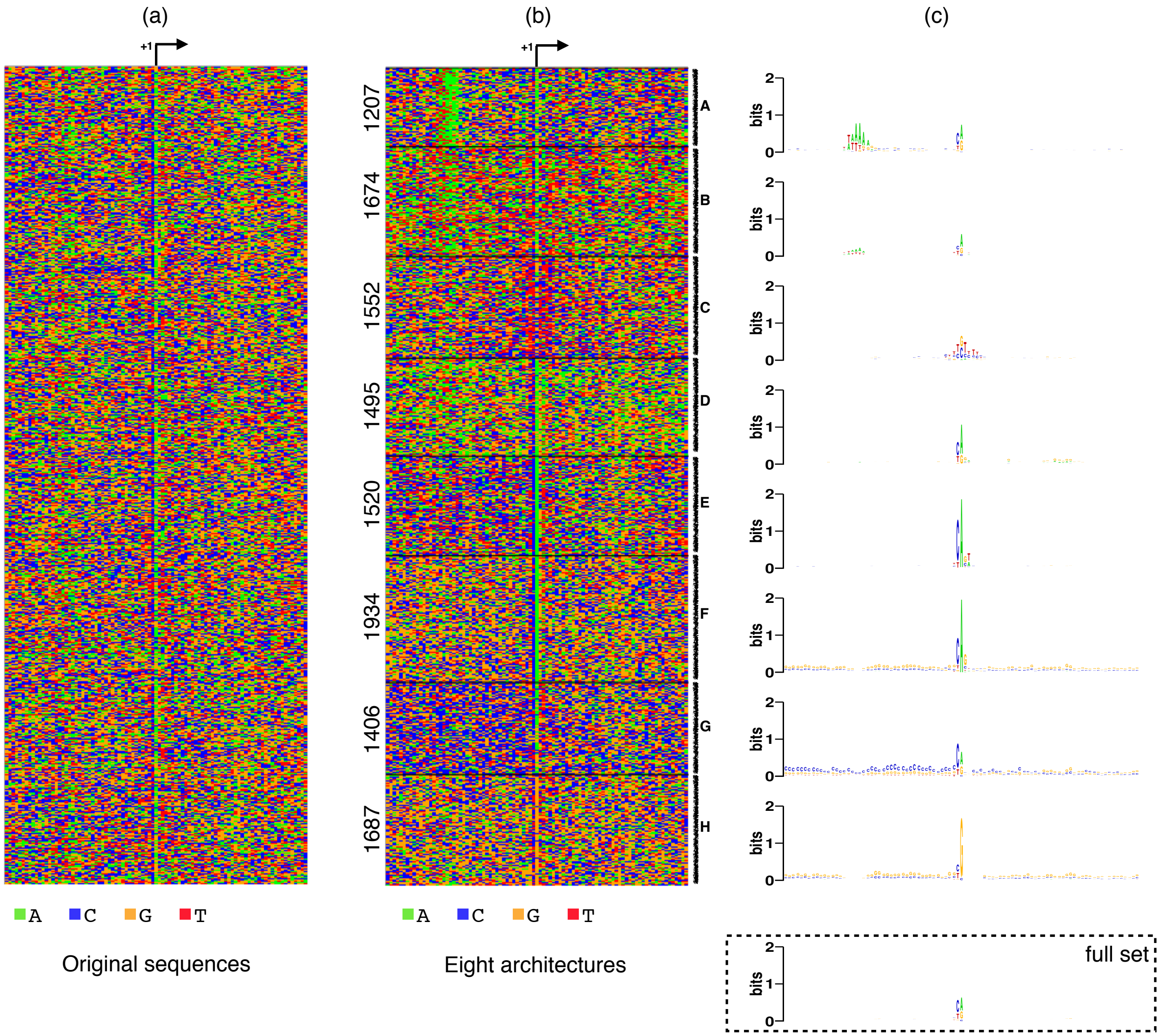
(a)
(b)
(c)
+1
+1
1207
1674
1552
1495
1520
1934
1406
1687
A
B
C
D
E
F
G
H
bits
2
1
0
A C G T
A C G T
Original sequences
Eight architectures
full set

Figure S7

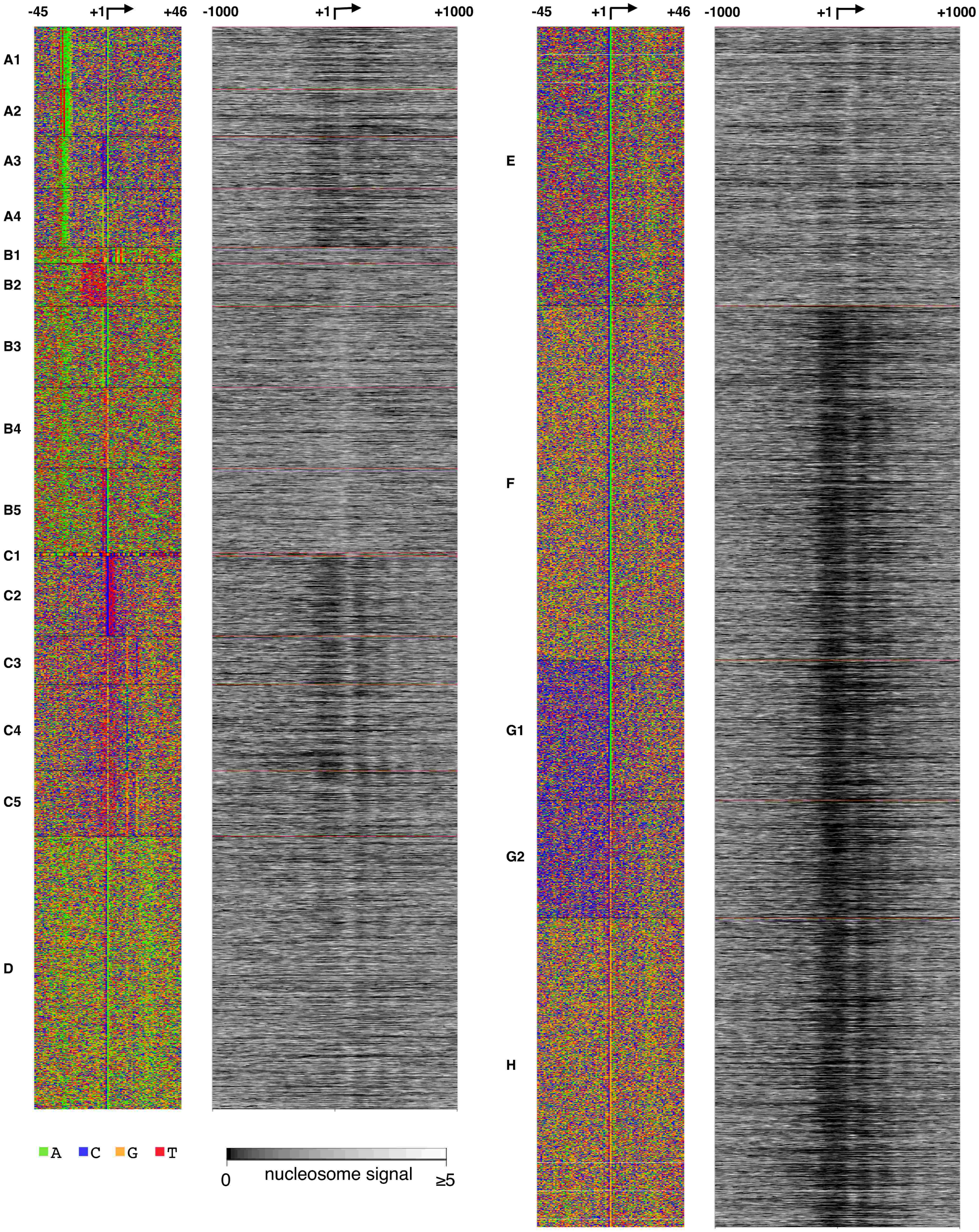
-45
+1
+46
-1000
+1
+1000
-45
+1
+46
-1000
+1
+1000
A1
A2
A3
A4
B1
B2
B3
B4
B5
C1
C2
C3
C4
C5
D
E
F
G1
G2
H
A
C
G
T
0
nucleosome signal
≥5

Figure S8

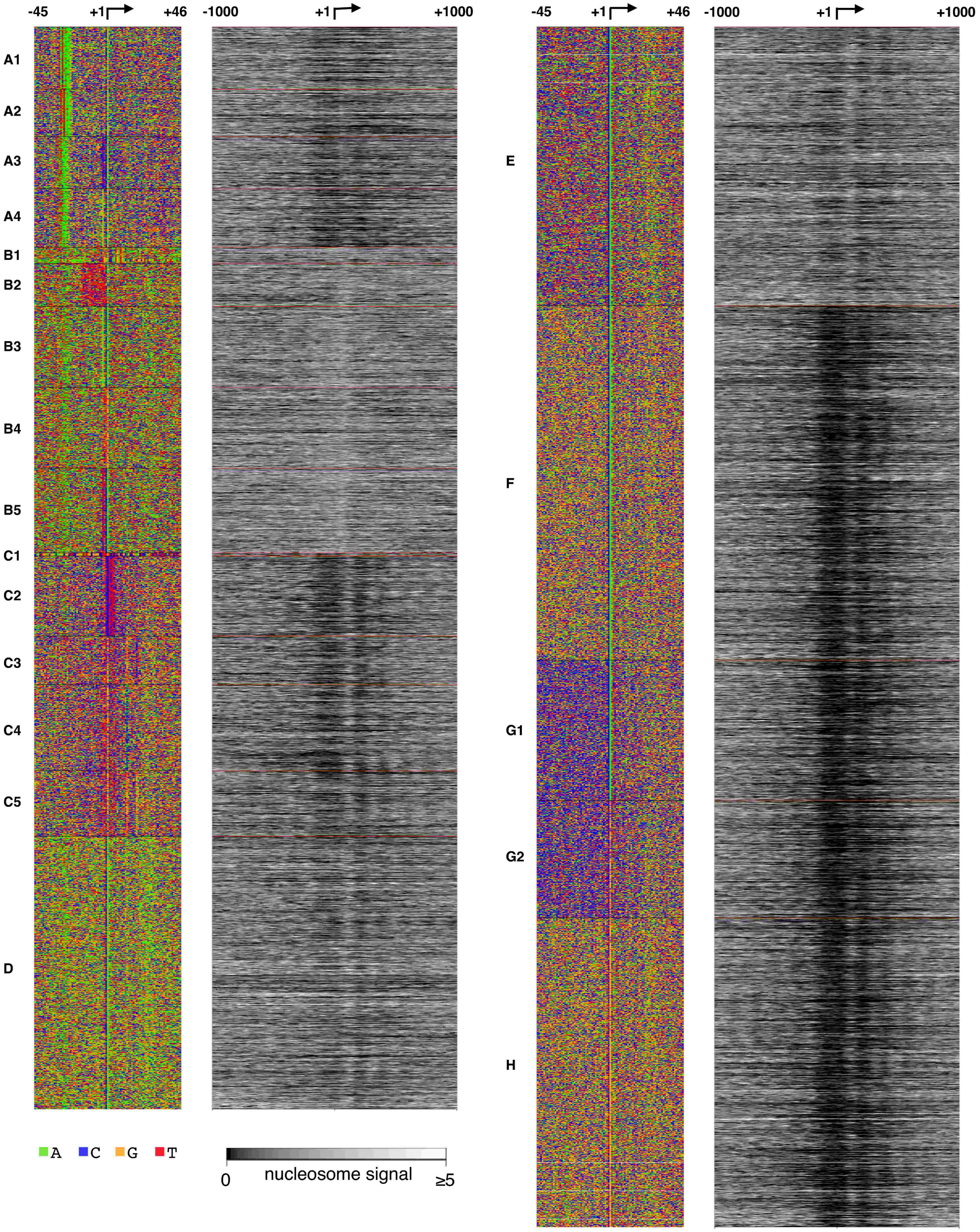

-45
+1
+46
-1000
+1
+1000
A1
A2
A3
A4
B1
B2
B3
B4
B5
C1
C2
C3
C4
C5
D
E
F
G1
G2
H
A
C
G
T
0
nucleosome signal
≥5

Figure S9

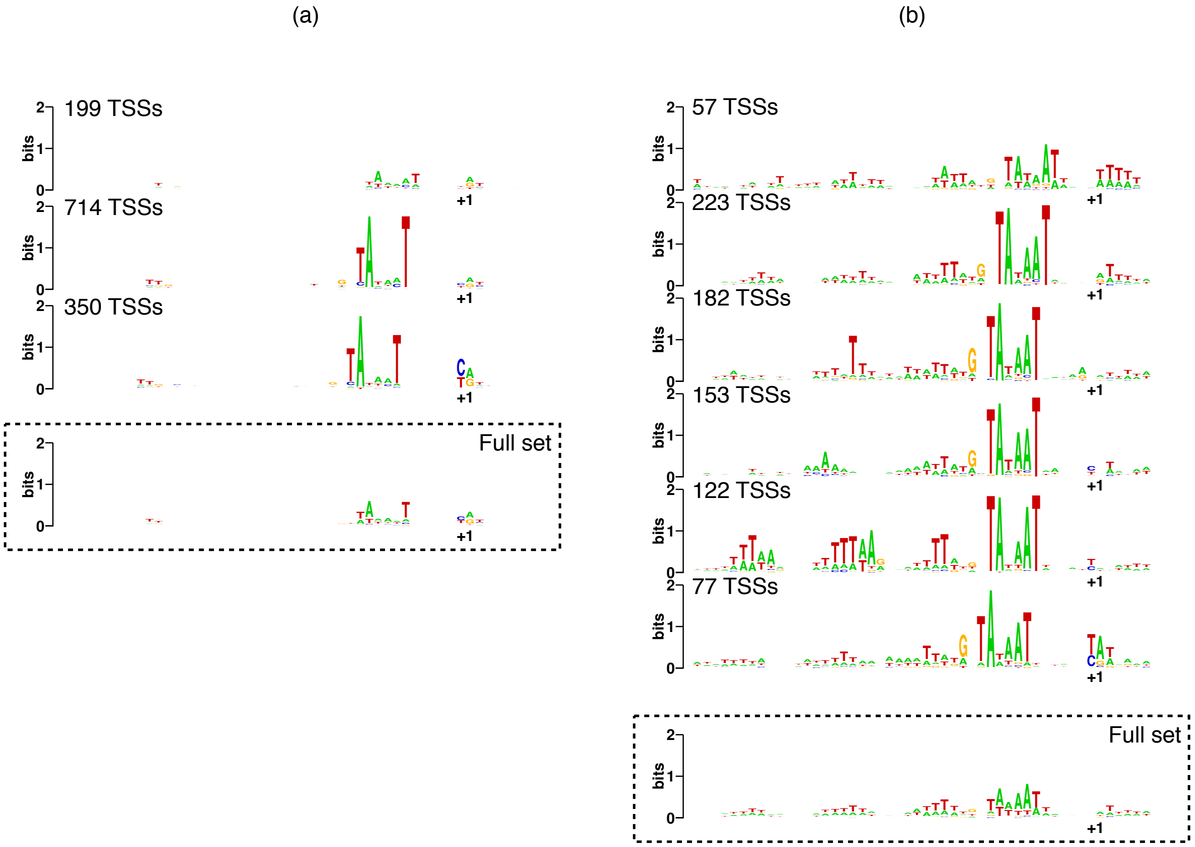
(a)
199 TSSs
714 TSSs
350 TSSs
Full set
bits
+1
(b)
57 TSSs
223 TSSs
182 TSSs
153 TSSs
122 TSSs
77 TSSs
Full set

Figure S10

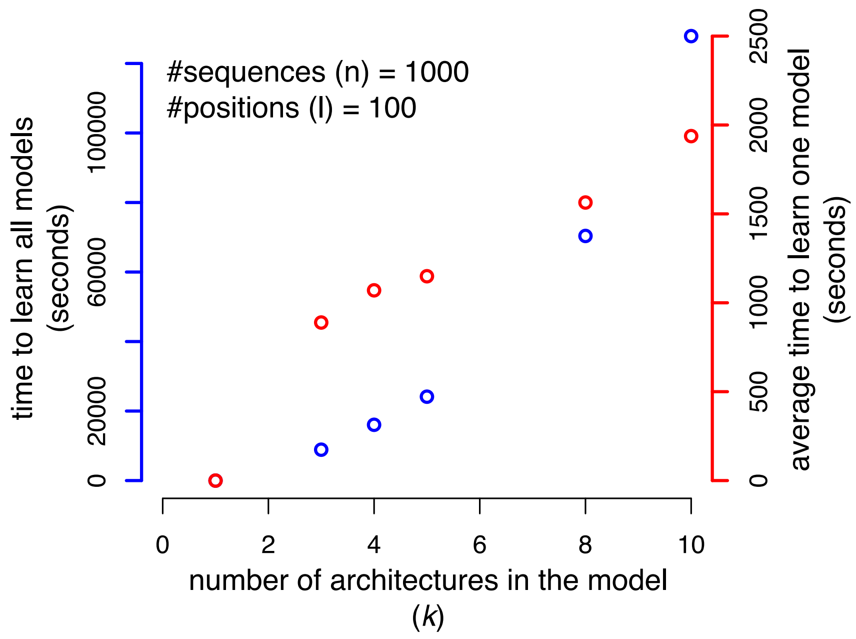
#sequences (n) = 1000
#positions (l) = 100
time to learn all models (seconds)
0
20000
60000
100000
average time to learn one model (seconds)
0
500
1000
1500
2000
2500
0
2
4
6
8
10
number of architectures in the model (k)

Figure S11

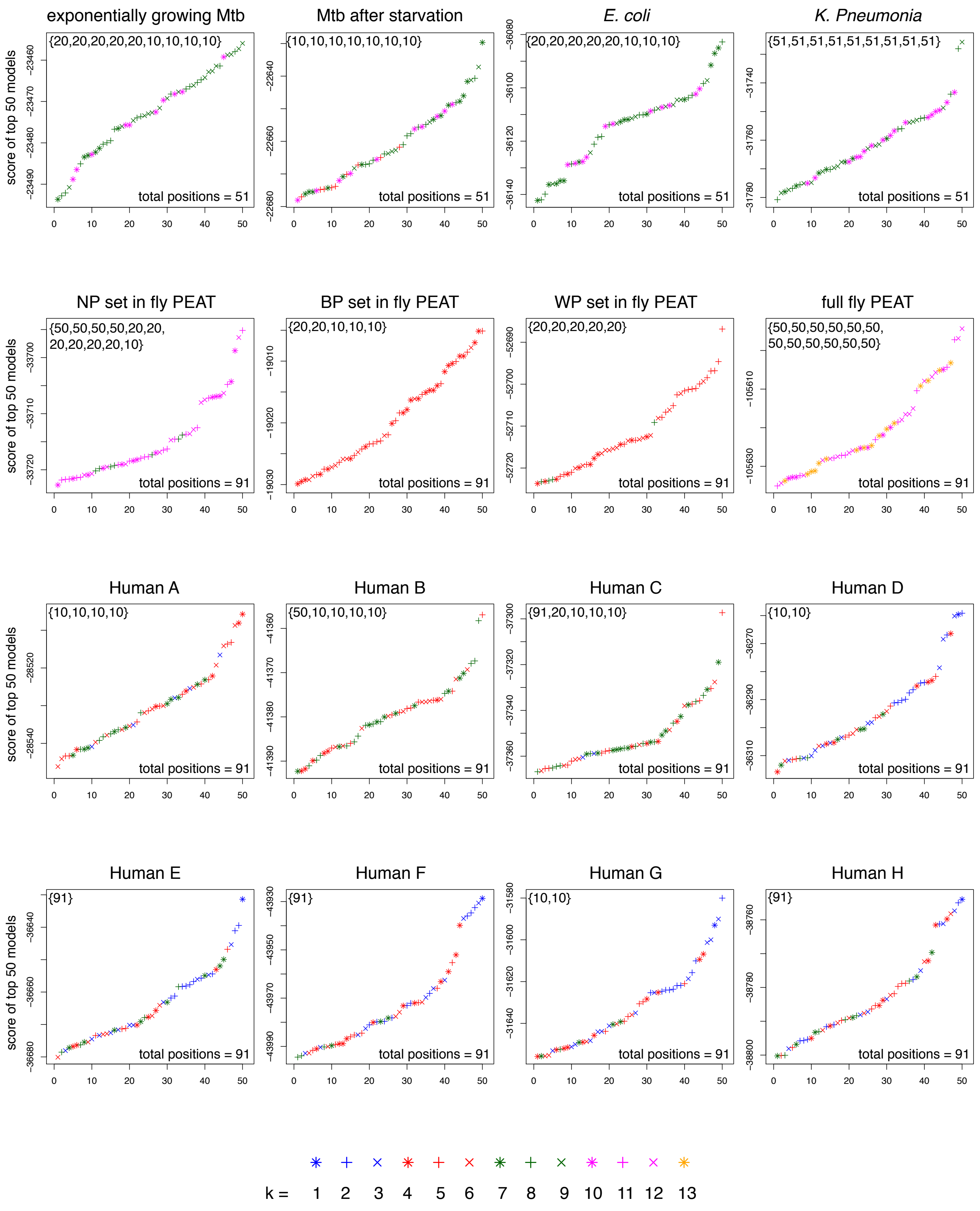

exponentially growing Mtb
{20,20,20,20,20,10,10,10,10}
total positions = 51
Mtb after starvation
{10,10,10,10,10,10,10}
total positions = 51
E. coli
{20,20,20,20,20,10,10,10}
total positions = 51
K. Pneumonia
{51,51,51,51,51,51,51,51,51}
total positions = 51
NP set in fly PEAT
{50,50,50,50,20,20,
20,20,20,20,10}
total positions = 91
BP set in fly PEAT
{20,20,10,10,10}
total positions = 91
WP set in fly PEAT
{20,20,20,20,20}
total positions = 91
full fly PEAT
{50,50,50,50,50,50,
50,50,50,50,50,50}
total positions = 91
Human A
{10,10,10,10}
total positions = 91
Human B
{50,10,10,10,10}
total positions = 91
Human C
{91,20,10,10,10}
total positions = 91
Human D
{10,10}
total positions = 91
Human E
{91}
total positions = 91
Human F
{91}
total positions = 91
Human G
{10,10}
total positions = 91
Human H
{91}
total positions = 91
score of top 50 models
k = 1 2 3 4 5 6 7 8 9 10 11 12 13

Figure S12
