# Supplementary material for: Multiple novel promoter-architectures revealed by decoding the hidden heterogeneity within the genome
Source: Nucleic Acids Res. 2014 Oct 17;42(20):12388–403. doi: 10.1093/nar/gku924 (PMC4227772; doi:10.1093/nar/gku924)
Supplement: SUPPLEMENTARY DATA [file supp_gku924_nar-02085-n-2014-File005.zip › TableS1.pdf]

Architecture NP1

Category	Term	Count	%	PValue	List Total	Pop Hits	Pop Total	Fold Enrichme	Bonferroni	Benjamini	FDR
SMART	SM00690:DM5	6	5.55555556	5.53E-07	33	26	4824	33.7342657	1.16E-05	1.16E-05	4.27E-04
INTERPRO	IPR004145:Protein of unknown function DUF243	6	5.55555556	8.33E-07	72	26	10196	32.6794872	9.50E-05	9.50E-05	9.48E-04
GOTERM_BP_FAT	GO:0042049~cellular acyl-CoA homeostasis	3	2.77777778	6.20E-04	45	7	7937	75.5904762	0.20841311	0.20841311	0.85357115
GOTERM_MF_FAT	GO:0050809~diazepam binding	3	2.77777778	6.73E-04	55	6	7918	71.9818182	0.100897	0.100897	0.80779332
GOTERM_MF_FAT	GO:0004857~enzyme inhibitor activity	6	5.55555556	8.00E-04	55	108	7918	7.9979798	0.11875191	0.06125185	0.95940529

Architecture NP2

Category	Term	Count	%	PValue	List Total	Pop Hits	Pop Total	Fold Enrichment	Bonferroni	Benjamini	FDR
GOTERM_MF_FAT	GO:0042302~structural constituent of cuticle	21	14.38356164	1.25E-21	66	120	7918	20.99469697	1.17E-19	1.17E-19	1.37E-18
GOTERM_MF_FAT	GO:0005214~structural constituent of chitin-based cuticle	20	13.69863014	7.31E-21	66	110	7918	21.81267218	6.88E-19	3.44E-19	8.02E-18
INTERPRO	IPR000618:Insect cuticle protein	20	13.69863014	4.66E-20	93	106	10196	20.68573747	4.29E-18	4.29E-18	5.10E-17
GOTERM_MF_FAT	GO:0005198~structural molecule activity	23	15.75342466	3.91E-12	66	456	7918	6.051103137	3.68E-10	1.23E-10	4.29E-09
INTERPRO	IPR004019:YLP motif	9	6.164383562	6.22E-11	93	27	10196	36.54480287	5.72E-09	2.86E-09	6.80E-08
GOTERM_MF_FAT	GO:0008010~structural constituent of chitin-based larval cuticle	8	5.479452055	2.26E-08	66	39	7918	24.60916861	2.12E-06	5.30E-07	2.48E-05
GOTERM_CC_FAT	GO:0005576~extracellular region	15	10.2739726	3.17E-08	26	512	4786	5.392878606	9.51E-07	9.51E-07	2.70E-05
SMART	SM00690:DM5	6	4.109589041	1.02E-06	37	26	4824	30.08731809	2.14E-05	2.14E-05	7.87E-04
INTERPRO	IPR004145:Protein of unknown function DUF243	6	4.109589041	3.04E-06	93	26	10196	25.30024814	2.79E-04	9.31E-05	0.003316574
INTERPRO	IPR012464:Protein of unknown function DUF1676	5	3.424657534	3.30E-05	93	21	10196	26.10343062	0.003034659	7.60E-04	0.036084285
UP_SEQ_FEATURE	signal peptide	14	9.589041096	1.93E-04	33	419	2975	3.012222463	0.015347639	0.015347639	0.205324306
UP_SEQ_FEATURE	domain:Chitin-binding type R&R	4	2.739726027	2.33E-04	33	12	2975	30.05050505	0.018469187	0.009277631	0.247424391
GOTERM_BP_FAT	GO:0007219~Notch signaling pathway	5	3.424657534	4.26E-04	50	58	7937	13.68448276	0.143611516	0.143611516	0.5841904
GOTERM_BP_FAT	GO:0006952~defense response	7	4.794520548	6.32E-04	50	173	7937	6.42300578	0.205561777	0.10868736	0.865913248
SP_PIR_KEYWORDS	cuticle	4	2.739726027	6.74E-04	145	16	12980	22.37931034	0.058206362	0.058206362	0.728772733
SP_PIR_KEYWORDS	signal	14	9.589041096	8.91E-04	145	425	12980	2.948803245	0.076245025	0.038878273	0.962658895

Architecture NP3

Category	Term	Count	%	PValue	List Total	Pop Hits	Pop Total	Fold Enrichment	Bonferroni	Benjamini	FDR
GOTERM_MF_FAT	GO:0005214~structural constituent of chitin-based cuticle	8	7.142857143	1.55E-05	60	110	7918	9.597575758	0.002215989	0.002215989	0.018370899
GOTERM_MF_FAT	GO:0042302~structural constituent of cuticle	8	7.142857143	2.74E-05	60	120	7918	8.797777778	0.003906759	0.001955291	0.032412827
INTERPRO	IPR000618:Insect cuticle protein	7	6.25	1.14E-04	75	106	10196	8.977610063	0.017241349	0.017241349	0.136854756
SP_PIR_KEYWORDS	stress response	4	3.571428571	8.79E-04	109	23	12980	20.71001197	0.079324917	0.079324917	0.959874533

Architecture NP4

Category	Term	Count	%	PValue	List Total	Pop Hits	Pop Total	Fold Enrichment	Bonferroni	Benjamini	FDR
INTERPRO	IPR001314:Peptidase S1A, chymotrypsin	8	10.52631579	6.70E-05	51	210	10196	7.616059757	0.007874772	0.007874772	0.076647414
SMART	SM00020:Tryp_SPc	8	10.52631579	7.65E-05	23	248	4824	6.765778401	0.001146505	0.001146505	0.053157578
INTERPRO	IPR001254:Peptidase S1 and S6, chymotrypsin/Hap	8	10.52631579	1.89E-04	51	248	10196	6.449082859	0.022089862	0.011106609	0.216408604
INTERPRO	IPR018114:Peptidase S1/S6, chymotrypsin/Hap, active site	7	9.210526316	3.20E-04	51	191	10196	7.326968484	0.037044914	0.012504003	0.365439434
SP_PIR_KEYWORDS	Protease	8	10.52631579	6.35E-04	73	264	12980	5.388127854	0.048308268	0.048308268	0.669225725
GOTERM_MF_FAT	GO:0004252~serine-type endopeptidase activity	8	10.52631579	6.93E-04	44	280	7918	5.141558442	0.085499955	0.085499955	0.802511649
SP_PIR_KEYWORDS	Serine protease	6	7.894736842	9.05E-04	73	135	12980	7.902587519	0.068162171	0.034682524	0.952810959

Architecture NP5

Category	Term	Count	%	PValue	List Total	Pop Hits	Pop Total	Fold Enrichment	Bonferroni	Benjamini	FDR
GOTERM_MF_FAT	GO:0016765~transferase activity, transferring alkyl or aryl (other than methyl) groups	5	5.376344086	3.83E-04	47	60	7918	14.03900709	0.047163048	0.047163048	0.44304621
KEGG_PATHWAY	dme00480:Glutathione metabolism	5	5.376344086	6.39E-04	15	63	2054	10.86772487	0.010810757	0.010810757	0.461880736
INTERPRO	IPR004019:YLP motif	4	4.301075269	6.78E-04	67	27	10196	22.54505252	0.084408502	0.084408502	0.78685497

Architecture NP6

Category	Term	Count	%	PValue	List Total	Pop Hits	Pop Total	Fold Enrichment	Bonferroni	Benjamini	FDR
GOTERM_BP_FAT	GO:0007444~imaginal disc development	18	12	7.30E-06	97	412	7937	3.574867381	0.007316395	0.007316395	0.011541116
INTERPRO	IPR002048:Calcium-binding EF-hand	8	5.333333333	3.09E-05	122	76	10196	8.797238999	0.008222453	0.008222453	0.040579265
INTERPRO	IPR018247:EF-HAND 1	9	6	4.08E-05	122	108	10196	6.964480874	0.010833828	0.005431665	0.053533809
GOTERM_BP_FAT	GO:0048707~instar larval or pupal morphogenesis	15	10	6.58E-05	97	347	7937	3.537092605	0.064054705	0.032557343	0.103992674
GOTERM_BP_FAT	GO:0009886~post-embryonic morphogenesis	15	10	7.92E-05	97	353	7937	3.476972051	0.076568464	0.026203445	0.125124707
SMART	SM00054:EFh	8	5.333333333	9.87E-05	71	76	4824	7.151964418	0.006294299	0.006294299	0.100178019
GOTERM_BP_FAT	GO:0007552~metamorphosis	15	10	1.04E-04	97	362	7937	3.390527995	0.09906971	0.025744655	0.163841719
INTERPRO	IPR018249:EF-HAND 2	8	5.333333333	1.30E-04	122	95	10196	7.037791199	0.034067129	0.011487159	0.170243146
GOTERM_BP_FAT	GO:0048563~post-embryonic organ morphogenesis	13	8.666666667	1.48E-04	97	284	7937	3.745498766	0.13874294	0.02943068	0.234484077
GOTERM_BP_FAT	GO:0007560~imaginal disc morphogenesis	13	8.666666667	1.48E-04	97	284	7937	3.745498766	0.13874294	0.02943068	0.234484077
GOTERM_BP_FAT	GO:0048569~post-embryonic organ development	13	8.666666667	2.26E-04	97	297	7937	3.581554375	0.203011712	0.037113	0.356017787
SP_PIR_KEYWORDS	egf-like domain	6	4	2.29E-04	143	51	12980	10.67873303	0.032424961	0.032424961	0.271050976
GOTERM_MF_FAT	GO:0005509~calcium ion binding	12	8	2.65E-04	103	242	7918	3.811923293	0.062344956	0.062344956	0.342019387
INTERPRO	IPR018248:EF hand	7	4.666666667	2.74E-04	122	76	10196	7.697584124	0.070530808	0.018119241	0.358908125
GOTERM_CC_FAT	GO:0005886~plasma membrane	23	15.33333333	2.98E-04	65	781	4786	2.168383729	0.044592847	0.044592847	0.356628354
SP_PIR_KEYWORDS	calcium	9	6	3.41E-04	143	158	12980	5.170399221	0.047956894	0.024273037	0.403853029
GOTERM_BP_FAT	GO:0048598~embryonic morphogenesis	11	7.333333333	3.80E-04	97	226	7937	3.982620199	0.317681293	0.053144084	0.599011583
GOTERM_BP_FAT	GO:0002165~instar larval or pupal development	15	10	4.61E-04	97	418	7937	2.936294579	0.371165405	0.056336726	0.72646065
GOTERM_CC_FAT	GO:0005578~proteinaceous extracellular matrix	6	4	4.86E-04	65	50	4786	8.835692308	0.071748439	0.036541874	0.581395792
GOTERM_BP_FAT	GO:0009891~positive regulation of biosynthetic process	9	6	5.02E-04	97	153	7937	4.813220133	0.396300548	0.054532176	0.790088771
GOTERM_BP_FAT	GO:0031328~positive regulation of cellular biosynthetic process	9	6	5.02E-04	97	153	7937	4.813220133	0.396300548	0.054532176	0.790088771
INTERPRO	IPR000742:EGF-like, type 3	6	4	5.10E-04	122	56	10196	8.954332553	0.127416824	0.026891274	0.667778443
GOTERM_BP_FAT	GO:0060429~epithelium development	11	7.333333333	5.17E-04	97	235	7937	3.830094319	0.405851796	0.050730606	0.814953316
GOTERM_CC_FAT	GO:0031012~extracellular matrix	6	4	6.39E-04	65	53	4786	8.335558781	0.093158249	0.032070256	0.762919505
GOTERM_BP_FAT	GO:0009791~post-embryonic development	15	10	6.71E-04	97	434	7937	2.828044088	0.490823678	0.059515474	1.055259268
GOTERM_BP_FAT	GO:0001736~establishment of planar polarity	6	4	8.12E-04	97	61	7937	8.048335305	0.558408367	0.065846161	1.276479988
SP_PIR_KEYWORDS	developmental protein	16	10.66666667	8.35E-04	143	540	12980	2.689458689	0.113324723	0.039299095	0.985495278
GOTERM_BP_FAT	GO:0007164~establishment of tissue polarity	6	4	8.75E-04	97	62	7937	7.918523445	0.585501382	0.065501305	1.374677287
GOTERM_BP_FAT	GO:0010557~positive regulation of macromolecule biosynthetic process	8	5.333333333	9.14E-04	97	129	7937	5.074402621	0.601513724	0.063607131	1.435728557
SP_PIR_KEYWORDS	glycoprotein	14	9.333333333	9.67E-04	143	435	12980	2.921308576	0.130082017	0.034239192	1.140932949

Architecture NP7

Category	Term	Count	%	PValue	List Total	Pop Hits	Pop Total	Fold Enrichment	Bonferroni	Benjamini	FDR
GOTERM_BP_FAT	GO:0007552~metamorphosis	17	9.770114943	1.16E-05	102	362	7937	3.654235727	0.009732796	0.009732796	0.017874652
GOTERM_BP_FAT	GO:0009791~post-embryonic development	17	9.770114943	1.05E-04	102	434	7937	3.048003072	0.085061517	0.043475833	0.162352165
GOTERM_BP_FAT	GO:0035150~regulation of tube size	5	2.873563218	1.82E-04	102	23	7937	16.91602728	0.142448208	0.049934707	0.280482951
GOTERM_BP_FAT	GO:0060429~epithelium development	12	6.896551724	1.84E-04	102	235	7937	3.973466834	0.144217145	0.038186441	0.284246426
GOTERM_BP_FAT	GO:0002165~instar larval or pupal development	16	9.195402299	2.38E-04	102	418	7937	2.978515808	0.182062239	0.03939675	0.366647261
GOTERM_BP_FAT	GO:0048729~tissue morphogenesis	12	6.896551724	2.84E-04	102	247	7937	3.78042391	0.213527775	0.039242246	0.438059262
SP_PIR_KEYWORDS	signal	16	9.195402299	3.88E-04	168	425	12980	2.908683473	0.054730844	0.054730844	0.459767044
GOTERM_BP_FAT	GO:0048707~instar larval or pupal morphogenesis	14	8.045977011	4.25E-04	102	347	7937	3.139458665	0.302239195	0.050112074	0.655609976
GOTERM_BP_FAT	GO:0000902~cell morphogenesis	16	9.195402299	4.32E-04	102	442	7937	2.816786443	0.306450385	0.044711187	0.666601251
GOTERM_BP_FAT	GO:0006928~cell motion	13	7.471264368	4.63E-04	102	305	7937	3.316650595	0.324377668	0.042633511	0.714136891
SP_PIR_KEYWORDS	glycoprotein	16	9.195402299	4.95E-04	168	435	12980	2.841817187	0.069293854	0.035268874	0.586219262
GOTERM_BP_FAT	GO:0009886~post-embryonic morphogenesis	14	8.045977011	5.01E-04	102	353	7937	3.086096762	0.345478671	0.041499419	0.771700653
GOTERM_BP_FAT	GO:0002009~morphogenesis of an epithelium	11	6.32183908	5.52E-04	102	225	7937	3.80422658	0.373284402	0.041588836	0.850402152
SP_PIR_KEYWORDS	developmental protein	18	10.34482759	5.93E-04	168	540	12980	2.575396825	0.082374965	0.028248802	0.701362408
GOTERM_BP_FAT	GO:0007474~imaginal disc-derived wing vein specification	5	2.873563218	6.76E-04	102	32	7937	12.15839461	0.435457887	0.046527814	1.039556934
GOTERM_BP_FAT	GO:0007409~axonogenesis	10	5.747126437	8.98E-04	102	198	7937	3.929986136	0.532543903	0.056818121	1.380297186
SP_PIR_KEYWORDS	alternative splicing	19	10.91954023	9.71E-04	168	616	12980	2.383078231	0.131390164	0.034602465	1.146649392

Architecture NP8

Category	Term	Count	%	PValue	List Total	Pop Hits	Pop Total	Fold Enrichment	Bonferroni	Benjamini	FDR
SP_PIR_KEYWORDS	alternative splicing	30	11.67315175	5.65E-06	247	616	12980	2.559282822	0.001004963	0.001004963	0.006944547
GOTERM_BP_FAT	GO:0007552~metamorphosis	20	7.782101167	4.76E-05	151	362	7937	2.904028393	0.051161913	0.051161913	0.076077561
UP_SEQ_FEATURE	splice variant	28	10.89494163	1.21E-04	68	604	2975	2.028145695	0.033899455	0.033899455	0.160309708
GOTERM_BP_FAT	GO:0060541~respiratory system development	12	4.6692607	1.42E-04	151	153	7937	4.122581483	0.145272894	0.075485476	0.227222951
GOTERM_BP_FAT	GO:0007424~open tracheal system development	12	4.6692607	1.42E-04	151	153	7937	4.122581483	0.145272894	0.075485476	0.227222951
GOTERM_BP_FAT	GO:0048569~post-embryonic organ development	17	6.614785992	1.45E-04	151	297	7937	3.008651638	0.147972872	0.051979328	0.231797441
GOTERM_BP_FAT	GO:0009791~post-embryonic development	21	8.171206226	1.80E-04	151	434	7937	2.543366802	0.18033124	0.04849824	0.287760947
SP_PIR_KEYWORDS	developmental protein	24	9.338521401	2.46E-04	247	540	12980	2.335582546	0.042887812	0.021678893	0.30230907
GOTERM_MF_FAT	GO:0003702~RNA polymerase II transcription factor activity	16	6.225680934	2.53E-04	164	257	7918	3.005789124	0.08708639	0.08708639	0.346998038
GOTERM_BP_FAT	GO:0048707~instar larval or pupal morphogenesis	18	7.003891051	2.79E-04	151	347	7937	2.726606485	0.265412355	0.059824946	0.445994843
GOTERM_BP_FAT	GO:0048666~neuron development	18	7.003891051	2.79E-04	151	347	7937	2.726606485	0.265412355	0.059824946	0.445994843
GOTERM_BP_FAT	GO:0007560~imaginal disc morphogenesis	16	6.225680934	2.92E-04	151	284	7937	2.961290924	0.275865483	0.05237493	0.466669855
GOTERM_BP_FAT	GO:0048563~post-embryonic organ morphogenesis	16	6.225680934	2.92E-04	151	284	7937	2.961290924	0.275865483	0.05237493	0.466669855
GOTERM_BP_FAT	GO:0002165~instar larval or pupal development	20	7.782101167	3.15E-04	151	418	7937	2.514971957	0.293601845	0.048441154	0.502432574
GOTERM_BP_FAT	GO:0009886~post-embryonic morphogenesis	18	7.003891051	3.41E-04	151	353	7937	2.680261899	0.314115348	0.046037319	0.544915512
GOTERM_BP_FAT	GO:0034330~cell junction organization	6	2.33463035	5.30E-04	151	36	7937	8.760485651	0.443012424	0.062954645	0.84448944
GOTERM_BP_FAT	GO:0001709~cell fate determination	10	3.891050584	7.21E-04	151	129	7937	4.074644489	0.548912885	0.076523088	1.14705178
GOTERM_BP_FAT	GO:0007444~imaginal disc development	19	7.392996109	7.30E-04	151	412	7937	2.424017874	0.553541783	0.070687256	1.161826976
GOTERM_BP_FAT	GO:0048729~tissue morphogenesis	14	5.447470817	7.74E-04	151	247	7937	2.979274472	0.574743166	0.068775754	1.231489295
GOTERM_MF_FAT	GO:0003779~actin binding	10	3.891050584	8.90E-04	164	122	7918	3.957417033	0.274270721	0.148102542	1.21556271
GOTERM_BP_FAT	GO:0006357~regulation of transcription from RNA polymerase II promoter	12	4.6692607	9.94E-04	151	192	7937	3.285182119	0.666341126	0.080967277	1.578083223

Architecture NP9

Category	Term	Count	%	PValue	List Total	Pop Hits	Pop Total	Fold Enrichment	Bonferroni	Benjamini	FDR
GOTERM_CC_FAT	GO:0022626~cytosolic ribosome	59	60.20408163	3.72E-92	79	92	4786	38.85167859	2.94E-90	2.94E-90	3.95E-89
SP_PIR_KEYWORDS	ribosomal protein	56	57.14285714	2.48E-84	95	157	12980	48.73483071	2.21E-82	2.21E-82	2.69E-81
KEGG_PATHWAY	dme03010:Ribosome	59	60.20408163	7.60E-84	64	88	2054	21.51740057	7.60E-83	7.60E-83	4.56E-81
GOTERM_MF_FAT	GO:0003735~structural constituent of ribosome	60	61.2244898	4.51E-81	86	178	7918	31.03475307	4.82E-79	4.82E-79	5.07E-78
GOTERM_CC_FAT	GO:0044445~cytosolic part	59	60.20408163	1.34E-80	79	127	4786	28.14452307	1.06E-78	5.29E-79	1.42E-77
SP_PIR_KEYWORDS	ribonucleoprotein	51	52.04081633	3.55E-78	95	131	12980	53.19244677	3.16E-76	1.58E-76	3.86E-75
GOTERM_CC_FAT	GO:0033279~ribosomal subunit	59	60.20408163	1.86E-72	79	164	4786	21.79484409	1.47E-70	4.89E-71	1.97E-69
GOTERM_CC_FAT	GO:0005840~ribosome	60	61.2244898	1.11E-70	79	186	4786	19.54267048	8.76E-69	2.19E-69	1.18E-67
GOTERM_BP_FAT	GO:0006412~translation	65	66.32653061	4.93E-59	86	520	7937	11.53633721	2.37E-56	2.37E-56	7.07E-56
GOTERM_CC_FAT	GO:0005829~cytosol	65	66.32653061	1.14E-54	79	437	4786	9.011094053	9.01E-53	1.80E-53	1.21E-51
GOTERM_MF_FAT	GO:0005198~structural molecule activity	60	61.2244898	2.33E-54	86	456	7918	12.11444308	2.49E-52	1.24E-52	2.61E-51
GOTERM_CC_FAT	GO:0030529~ribonucleoprotein complex	61	62.24489796	5.22E-52	79	379	4786	9.750709729	4.13E-50	6.88E-51	5.54E-49
GOTERM_BP_FAT	GO:0000022~mitotic spindle elongation	36	36.73469388	4.48E-51	86	78	7937	42.59570662	2.15E-48	1.08E-48	6.41E-48
GOTERM_BP_FAT	GO:0051231~spindle elongation	36	36.73469388	7.99E-51	86	79	7937	42.05652046	3.84E-48	1.28E-48	1.14E-47
GOTERM_CC_FAT	GO:0022625~cytosolic large ribosomal subunit	34	34.69387755	4.06E-48	79	55	4786	37.45086306	3.21E-46	4.58E-47	4.30E-45
GOTERM_CC_FAT	GO:0015934~large ribosomal subunit	34	34.69387755	1.29E-36	79	102	4786	20.19409283	1.02E-34	1.27E-35	1.36E-33
GOTERM_BP_FAT	GO:0007052~mitotic spindle organization	37	37.75510204	1.47E-36	86	194	7937	17.60183409	7.06E-34	1.76E-34	2.10E-33
GOTERM_CC_FAT	GO:0022627~cytosolic small ribosomal subunit	25	25.51020408	2.05E-35	79	38	4786	39.85676216	1.62E-33	1.80E-34	2.18E-32
GOTERM_BP_FAT	GO:0007051~spindle organization	37	37.75510204	4.15E-34	86	225	7937	15.17669251	2.00E-31	3.99E-32	5.95E-31
SP_PIR_KEYWORDS	ribosome	18	18.36734694	9.26E-32	95	25	12980	98.37473684	8.24E-30	2.75E-30	1.01E-28
GOTERM_CC_FAT	GO:0043232~intracellular non-membrane-bounded organelle	63	64.28571429	2.20E-30	79	943	4786	4.047384458	1.74E-28	1.74E-29	2.33E-27
GOTERM_CC_FAT	GO:0043228~non-membrane-bounded organelle	63	64.28571429	2.20E-30	79	943	4786	4.047384458	1.74E-28	1.74E-29	2.33E-27
GOTERM_BP_FAT	GO:0000226~microtubule cytoskeleton organization	37	37.75510204	1.37E-29	86	298	7937	11.45891213	6.59E-27	1.10E-27	1.96E-26
GOTERM_CC_FAT	GO:0015935~small ribosomal subunit	25	25.51020408	1.48E-27	79	67	4786	22.60532779	1.17E-25	1.06E-26	1.57E-24
GOTERM_BP_FAT	GO:0000278~mitotic cell cycle	37	37.75510204	1.01E-26	86	358	7937	9.538424061	4.84E-24	6.92E-25	1.44E-23
SP_PIR_KEYWORDS	protein biosynthesis	22	22.44897959	2.54E-26	95	93	12980	32.32144878	2.26E-24	5.65E-25	2.76E-23
GOTERM_BP_FAT	GO:0007017~microtubule-based process	37	37.75510204	8.53E-25	86	406	7937	8.410728606	4.10E-22	5.13E-23	1.22E-21
GOTERM_BP_FAT	GO:0007010~cytoskeleton organization	38	38.7755102	7.22E-24	86	465	7937	7.542035509	3.47E-21	3.86E-22	1.03E-20
GOTERM_CC_FAT	GO:0005811~lipid particle	34	34.69387755	6.98E-23	79	249	4786	8.272278989	5.51E-21	4.60E-22	7.40E-20
GOTERM_BP_FAT	GO:0000279~M phase	37	37.75510204	2.43E-22	86	478	7937	7.143840615	1.17E-19	1.17E-20	3.48E-19
GOTERM_BP_FAT	GO:0022403~cell cycle phase	37	37.75510204	8.58E-22	86	496	7937	6.884588335	4.13E-19	3.75E-20	1.23E-18
GOTERM_BP_FAT	GO:0022402~cell cycle process	37	37.75510204	2.35E-20	86	547	7937	6.242698015	1.13E-17	9.42E-19	3.36E-17
GOTERM_BP_FAT	GO:0007049~cell cycle	37	37.75510204	1.22E-18	86	616	7937	5.543434763	5.87E-16	4.51E-17	1.75E-15
GOTERM_MF_FAT	GO:0032183~SUMO binding	11	11.2244898	1.31E-09	86	65	7918	15.58103757	1.40E-07	4.67E-08	1.47E-06
GOTERM_MF_FAT	GO:0032182~small conjugating protein binding	11	11.2244898	1.53E-09	86	66	7918	15.34496124	1.64E-07	4.09E-08	1.72E-06
SP_PIR_KEYWORDS	rrna-binding	6	6.12244898	4.38E-09	95	10	12980	81.97894737	3.90E-07	7.80E-08	4.76E-06
GOTERM_MF_FAT	GO:0019843~rRNA binding	7	7.142857143	1.12E-07	86	23	7918	28.02123357	1.19E-05	2.39E-06	1.26E-04
SP_PIR_KEYWORDS	cytoplasm	15	15.30612245	2.83E-06	95	436	12980	4.700627716	2.52E-04	4.19E-05	0.003069626
SP_PIR_KEYWORDS	rna-binding	9	9.183673469	6.87E-06	95	138	12980	8.910755149	6.11E-04	8.74E-05	0.007459126
SP_PIR_KEYWORDS	elongation factor	4	4.081632653	7.72E-05	95	12	12980	45.54385965	0.006845839	8.58E-04	0.083750521
SMART	SM00739:KOW	3	3.06122449	1.79E-04	22	5	4824	131.5636364	0.004289586	0.004289586	0.143582175
INTERPRO	IPR005824:KOW	3	3.06122449	8.09E-04	94	5	10196	65.08085106	0.168408076	0.168408076	1.030165981

Architecture NP10

Category	Term	Count	%	PValue	List Total	Pop Hits	Pop Total	Fold Enrichment	Bonferroni	Benjamini	FDR
SP_PIR_KEYWORDS	mRNA transport	3	8.571428571	7.83E-04	33	17	12980	69.41176471	0.046630387	0.046630387	0.7841915
SP_PIR_KEYWORDS	protein transport	4	11.42857143	0.00192532	33	101	12980	15.57755776	0.110910807	0.057084737	1.919476521
GOTERM_BP_FAT	GO:0051028~mRNA transport	3	8.571428571	0.003779282	24	32	7937	31.00390625	0.742193565	0.742193565	5.063347946
SP_PIR_KEYWORDS	nucleus	8	22.85714286	0.004520404	33	869	12980	3.62102033	0.241467606	0.088007268	4.454176605
GOTERM_CC_FAT	GO:0043233~organelle lumen	7	20	0.006879583	17	556	4786	3.54443504	0.246508564	0.246508564	6.155350946
GOTERM_CC_FAT	GO:0070013~intracellular organelle lumen	7	20	0.006879583	17	556	4786	3.54443504	0.246508564	0.246508564	6.155350946
GOTERM_CC_FAT	GO:0031974~membrane-enclosed lumen	7	20	0.007844675	17	571	4786	3.451323787	0.275953634	0.149090859	6.991262685
GOTERM_BP_FAT	GO:0050657~nucleic acid transport	3	8.571428571	0.01409272	24	63	7937	15.74801587	0.993786814	0.921176237	17.69730152
GOTERM_BP_FAT	GO:0050658~RNA transport	3	8.571428571	0.01409272	24	63	7937	15.74801587	0.993786814	0.921176237	17.69730152
GOTERM_BP_FAT	GO:0042254~ribosome biogenesis	3	8.571428571	0.014521863	24	64	7937	15.50195313	0.994683525	0.825468187	18.18755262
GOTERM_BP_FAT	GO:0051236~establishment of RNA localization	3	8.571428571	0.014521863	24	64	7937	15.50195313	0.994683525	0.825468187	18.18755262
GOTERM_BP_FAT	GO:0015931~nucleobase, nucleoside, nucleotide and nucleic acid transport	3	8.571428571	0.016294592	24	68	7937	14.59007353	0.997209442	0.7701615	20.18415787
SP_PIR_KEYWORDS	nuclear pore complex	2	5.714285714	0.021977139	33	9	12980	87.40740741	0.742196161	0.287438514	20.02742024
GOTERM_BP_FAT	GO:0030035~microspike assembly	2	5.714285714	0.022958818	24	8	7937	82.67708333	0.999755202	0.810433558	27.2928722
GOTERM_BP_FAT	GO:0046847~filopodium assembly	2	5.714285714	0.022958818	24	8	7937	82.67708333	0.999755202	0.810433558	27.2928722
GOTERM_BP_FAT	GO:0008104~protein localization	5	14.28571429	0.024609428	24	392	7937	4.218218537	0.999866364	0.773889693	28.960456
GOTERM_BP_FAT	GO:0034984~cellular response to DNA damage stimulus	3	8.571428571	0.024788382	24	85	7937	11.67205882	0.999874861	0.722996738	29.13910388
GOTERM_BP_FAT	GO:0022613~ribonucleoprotein complex biogenesis	3	8.571428571	0.029877116	24	94	7937	10.55452128	0.99998077	0.742666088	34.04815072
GOTERM_BP_FAT	GO:0008360~regulation of cell shape	3	8.571428571	0.032262384	24	98	7937	10.12372449	0.999992034	0.728687454	36.23892378
GOTERM_CC_FAT	GO:0005635~nuclear envelope	3	8.571428571	0.032529739	17	86	4786	9.820793434	0.742283278	0.363623403	26.23887474
GOTERM_BP_FAT	GO:0015031~protein transport	4	11.42857143	0.044865365	24	278	7937	4.758393285	0.999999927	0.80666412	46.73625263
GOTERM_BP_FAT	GO:0008345~larval locomotory behavior	2	5.714285714	0.045412941	24	16	7937	41.33854167	0.999999941	0.779662889	47.1537652
GOTERM_BP_FAT	GO:0022604~regulation of cell morphogenesis	3	8.571428571	0.045946547	24	119	7937	8.337184874	0.999999951	0.754197792	47.55770427
GOTERM_BP_FAT	GO:0045184~establishment of protein localization	4	11.42857143	0.047724093	24	285	7937	4.641520468	0.999999975	0.739888176	48.88274759
GOTERM_BP_FAT	GO:0016476~regulation of embryonic cell shape	2	5.714285714	0.048184751	24	17	7937	38.90686275	0.999999979	0.717146969	49.22103671
GOTERM_BP_FAT	GO:0006403~RNA localization	3	8.571428571	0.050191358	24	125	7937	7.937	0.99999999	0.707417291	50.67053858
UP_SEQ_FEATURE	compositionally biased region:Poly-Gln	3	8.571428571	0.05408291	13	95	2975	7.226720648	0.922510777	0.922510777	40.87544917
SP_PIR_KEYWORDS	ribosome biogenesis	2	5.714285714	0.055236699	33	23	12980	34.20289855	0.968760511	0.500033639	43.52886665
GOTERM_CC_FAT	GO:0005730~nucleolus	3	8.571428571	0.064873851	17	126	4786	6.703081232	0.936073894	0.497171874	46.05793844
SP_PIR_KEYWORDS	translocation	2	5.714285714	0.071452932	33	30	12980	26.22222222	0.989134761	0.529376927	52.55309493
SP_PIR_KEYWORDS	cytoplasm	4	11.42857143	0.091182442	33	436	12980	3.608562691	0.997068572	0.565335494	61.76991972
GOTERM_CC_FAT	GO:0005759~mitochondrial matrix	3	8.571428571	0.095038972	17	157	4786	5.3795429	0.983334247	0.559074729	60.10869222
GOTERM_CC_FAT	GO:0031980~mitochondrial lumen	3	8.571428571	0.095038972	17	157	4786	5.3795429	0.983334247	0.559074729	60.10869222
GOTERM_BP_FAT	GO:0030537~larval behavior	2	5.714285714	0.096780297	24	35	7937	18.89761905	1	0.89746376	75.26199854

Architecture NP11

Category	Term	Count	%	PValue	List Total	Pop Hits	Pop Total	Fold Enrichment	Bonferroni	Benjamini	FDR
NONE											

Architecture BP1

Category	Term	Count	%	PValue	List Total	Pop Hits	Pop Total	Fold Enrichment	Bonferroni	Benjamini	FDR
GOTERM_CC_FAT	GO:0043233~organelle lumen	24	21.23893805	5.09E-08	60	556	4786	3.443165468	8.85E-06	8.85E-06	6.23E-05
GOTERM_CC_FAT	GO:0070013~intracellular organelle lumen	24	21.23893805	5.09E-08	60	556	4786	3.443165468	8.85E-06	8.85E-06	6.23E-05
GOTERM_CC_FAT	GO:0031974~membrane-enclosed lumen	24	21.23893805	8.42E-08	60	571	4786	3.352714536	1.46E-05	7.32E-06	1.03E-04
GOTERM_CC_FAT	GO:0031981~nuclear lumen	15	13.27433628	1.84E-04	60	387	4786	3.091731266	0.03143652	0.010590605	0.22457938
GOTERM_CC_FAT	GO:0005654~nucleoplasm	12	10.61946903	3.16E-04	60	265	4786	3.612075472	0.053525541	0.013658685	0.38647138
GOTERM_CC_FAT	GO:0044451~nucleoplasm part	11	9.734513274	5.96E-04	60	240	4786	3.655972222	0.09848498	0.020522208	0.727128769
GOTERM_CC_FAT	GO:0005759~mitochondrial matrix	9	7.96460177	5.99E-04	60	157	4786	4.572611465	0.099056438	0.017235189	0.731559503
GOTERM_CC_FAT	GO:0031980~mitochondrial lumen	9	7.96460177	5.99E-04	60	157	4786	4.572611465	0.099056438	0.017235189	0.731559503

Architecture BP2

Category	Term	Count	%	PValue	List Total	Pop Hits	Pop Total	Fold Enrichment	Bonferroni	Benjamini	FDR
GOTERM_BP_FAT	GO:0043933~macromolecular complex subunit organization	17	8.947368421	4.47E-06	121	281	7937	3.968383283	0.004346374	0.004346374	0.00703555
GOTERM_BP_FAT	GO:0065003~macromolecular complex assembly	15	7.894736842	1.87E-05	121	246	7937	3.999697642	0.018030215	0.009056114	0.029384801
GOTERM_BP_FAT	GO:0034621~cellular macromolecular complex subunit organization	12	6.315789474	2.37E-04	121	203	7937	3.877539389	0.206423484	0.074173511	0.372758737
GOTERM_CC_FAT	GO:0005811~lipid particle	14	7.368421053	4.31E-04	87	249	4786	3.093015741	0.083794531	0.083794531	0.540200226
SP_PIR_KEYWORDS	cytoplasm	17	8.947368421	4.78E-04	185	436	12980	2.735680635	0.076367343	0.076367343	0.579799423
GOTERM_CC_FAT	GO:0070013~intracellular organelle lumen	22	11.57894737	6.20E-04	87	556	4786	2.176713801	0.118291888	0.061006863	0.776183898
GOTERM_CC_FAT	GO:0043233~organelle lumen	22	11.57894737	6.20E-04	87	556	4786	2.176713801	0.118291888	0.061006863	0.776183898
GOTERM_BP_FAT	GO:0034622~cellular macromolecular complex assembly	10	5.263157895	8.48E-04	121	165	7937	3.97545705	0.56267905	0.186794943	1.327072419
GOTERM_BP_FAT	GO:0046907~intracellular transport	14	7.368421053	8.51E-04	121	313	7937	2.93396351	0.564024719	0.15298252	1.331984156
GOTERM_CC_FAT	GO:0031974~membrane-enclosed lumen	22	11.57894737	8.84E-04	87	571	4786	2.119532178	0.164261166	0.058059376	1.104478965

Architecture BP3

Category	Term	Count	%	PValue	List Total	Pop Hits	Pop Total	Fold Enrichment	Bonferroni	Benjamini	FDR
GOTERM_BP_FAT	GO:0006412~translation	34	44.73684211	1.17E-24	59	520	7937	8.79589309	4.38E-22	4.38E-22	1.62E-21
SP_PIR_KEYWORDS	protein biosynthesis	16	21.05263158	1.69E-18	73	93	12980	30.59066136	1.43E-16	1.43E-16	1.81E-15
GOTERM_CC_FAT	GO:0005829~cytosol	30	39.47368421	6.51E-18	52	437	4786	6.318429854	6.45E-16	6.45E-16	7.21E-15
KEGG_PATHWAY	dme03010:Ribosome	18	23.68421053	6.68E-18	28	88	2054	15.00487013	1.54E-16	1.54E-16	5.30E-15
SP_PIR_KEYWORDS	ribonucleoprotein	17	22.36842105	1.12E-17	73	131	12980	23.07434905	9.55E-16	4.78E-16	1.21E-14
GOTERM_CC_FAT	GO:0022626~cytosolic ribosome	18	23.68421053	1.22E-17	52	92	4786	18.00752508	1.21E-15	6.05E-16	1.36E-14
SP_PIR_KEYWORDS	Initiation factor	12	15.78947368	2.56E-17	73	35	12980	60.962818	2.18E-15	7.26E-16	2.76E-14
GOTERM_CC_FAT	GO:0044445~cytosolic part	19	25	1.61E-16	52	127	4786	13.76953362	1.10E-14	3.66E-15	1.22E-13
SP_PIR_KEYWORDS	ribosomal protein	17	22.36842105	2.14E-16	73	157	12980	19.25311927	1.89E-14	4.66E-15	2.44E-13
GOTERM_MF_FAT	GO:0003743~translation initiation factor activity	13	17.10526316	1.17E-14	63	58	7918	28.17022441	1.70E-12	1.70E-12	1.39E-11
GOTERM_MF_FAT	GO:0003735~structural constituent of ribosome	18	23.68421053	1.36E-14	63	178	7918	12.7094703	1.98E-12	9.89E-13	1.61E-11
GOTERM_CC_FAT	GO:0033279~ribosomal subunit	18	23.68421053	2.92E-13	52	164	4786	10.10178236	2.89E-11	7.22E-12	3.23E-10
GOTERM_MF_FAT	GO:0008135~translation factor activity, nucleic acid binding	13	17.10526316	1.10E-12	63	83	7918	19.68521706	1.60E-10	5.34E-11	1.30E-09
GOTERM_CC_FAT	GO:0005840~ribosome	18	23.68421053	2.36E-12	52	186	4786	8.906947891	2.34E-10	4.68E-11	2.62E-09
GOTERM_BP_FAT	GO:0006413~translational initiation	11	14.47368421	4.38E-12	59	55	7937	26.90508475	1.64E-09	8.19E-10	6.05E-09
GOTERM_CC_FAT	GO:0030529~ribonucleoprotein complex	23	30.26315789	5.99E-12	52	379	4786	5.585447534	5.93E-10	9.89E-11	6.64E-09
GOTERM_BP_FAT	GO:0000022~mitotic spindle elongation	12	15.78947368	6.27E-12	59	78	7937	20.69621904	2.34E-09	7.82E-10	8.66E-09
GOTERM_BP_FAT	GO:0051231~spindle elongation	12	15.78947368	7.24E-12	59	79	7937	20.43424158	2.71E-09	6.77E-10	1.00E-08
GOTERM_CC_FAT	GO:0022625~cytosolic large ribosomal subunit	11	14.47368421	1.52E-10	52	55	4786	18.40769231	1.51E-08	2.16E-09	1.69E-07
GOTERM_MF_FAT	GO:0005198~structural molecule activity	20	26.31578947	8.22E-10	63	456	7918	5.512392091	1.20E-07	3.00E-08	9.77E-07
GOTERM_CC_FAT	GO:0005852~eukaryotic translation initiation factor 3 complex	7	9.210526316	7.99E-09	52	16	4786	40.26682692	7.91E-07	9.89E-08	8.85E-06
GOTERM_BP_FAT	GO:0007052~mitotic spindle organization	13	17.10526316	1.08E-08	59	194	7937	9.01459025	4.04E-06	8.07E-07	1.49E-05
GOTERM_BP_FAT	GO:0007051~spindle organization	13	17.10526316	5.66E-08	59	225	7937	7.772580038	2.12E-05	3.53E-06	7.82E-05
GOTERM_CC_FAT	GO:0015934~large ribosomal subunit	11	14.47368421	7.66E-08	52	102	4786	9.92571644	7.58E-06	8.42E-07	8.48E-05
GOTERM_CC_FAT	GO:0043232~intracellular non-membrane-bounded organelle	27	35.52631579	5.10E-07	52	943	4786	2.635247573	5.05E-05	5.05E-06	5.65E-04
GOTERM_CC_FAT	GO:0043228~non-membrane-bounded organelle	27	35.52631579	5.10E-07	52	943	4786	2.635247573	5.05E-05	5.05E-06	5.65E-04
GOTERM_BP_FAT	GO:0000226~microtubule cytoskeleton organization	13	17.10526316	1.19E-06	59	298	7937	5.868558753	4.45E-04	6.36E-05	0.001644964
GOTERM_CC_FAT	GO:0022627~cytosolic small ribosomal subunit	7	9.210526316	2.31E-06	52	38	4786	16.95445344	2.28E-04	2.07E-05	0.00255405
SP_PIR_KEYWORDS	cytoplasm	13	17.10526316	4.38E-06	73	436	12980	5.301621214	3.72E-04	7.45E-05	0.004712053
GOTERM_CC_FAT	GO:0005811~lipid particle	13	17.10526316	7.80E-06	52	249	4786	4.805220884	7.71E-04	6.43E-05	0.008635236
GOTERM_BP_FAT	GO:0000278~mitotic cell cycle	13	17.10526316	8.03E-06	59	358	7937	4.88500142	0.002999397	3.75E-04	0.011092612
INTERPRO	IPR012340:Nucleic acid-binding, OB-fold	6	7.894736842	1.43E-05	74	44	10196	18.78869779	0.002743955	0.002743955	0.017815914
GOTERM_BP_FAT	GO:0007010~cytoskeleton organization	14	18.42105263	2.25E-05	59	465	7937	4.050227811	0.008384955	9.35E-04	0.031090823
GOTERM_BP_FAT	GO:0007017~microtubule-based process	13	17.10526316	2.85E-05	59	406	7937	4.307464307	0.010602238	0.001065316	0.039354681
GOTERM_BP_FAT	GO:0000279~M phase	14	18.42105263	3.01E-05	59	478	7937	3.940075172	0.01120793	0.001024131	0.04161521
SP_PIR_KEYWORDS	phosphoprotein	16	21.05263158	3.25E-05	73	815	12980	3.490713505	0.002757637	4.60E-04	0.03494642
GOTERM_BP_FAT	GO:0022403~cell cycle phase	14	18.42105263	4.44E-05	59	496	7937	3.797088573	0.016468096	0.00138281	0.06130316
GOTERM_CC_FAT	GO:0015935~small ribosomal subunit	7	9.210526316	6.59E-05	52	67	4786	9.615958668	0.006501669	5.02E-04	0.072966152
GOTERM_BP_FAT	GO:0007049~cell cycle	15	19.73684211	1.02E-04	59	616	7937	3.275781422	0.037411891	0.002928755	0.140711375
GOTERM_BP_FAT	GO:0022402~cell cycle process	14	18.42105263	1.21E-04	59	547	7937	3.443063861	0.044380695	0.003237296	0.16750285
GOTERM_MF_FAT	GO:0000049~tRNA binding	4	5.263157895	1.57E-04	63	14	7918	35.90929705	0.022593338	0.004560067	0.185868193
SP_PIR_KEYWORDS	ribosome	4	5.263157895	3.45E-04	73	25	12980	28.44931507	0.028889647	0.004179124	0.370364176
GOTERM_BP_FAT	GO:0022613~ribonucleoprotein complex biogenesis	6	7.894736842	5.84E-04	59	94	7937	8.586729174	0.196399336	0.014471127	0.804219672
SP_PIR_KEYWORDS	rna-binding	6	7.894736842	9.99E-04	73	138	12980	7.730792138	0.081412499	0.010558626	1.069080736

Architecture BP4

Category	Term	Count	%	PValue	List Total	Pop Hits	Pop Total	Fold Enrichment	Bonferroni	Benjamini	FDR
SP_PIR_KEYWORDS	alternative splicing	33	12.45283019	3.90E-07	254	616	12980	2.737626547	7.95E-05	7.95E-05	4.90E-04
GOTERM_BP_FAT	GO:0055114~oxidation reduction	32	12.0754717	1.69E-06	161	620	7937	2.544419956	0.001919178	0.001919178	0.002706831
GOTERM_CC_FAT	GO:0005811~lipid particle	21	7.924528302	1.78E-06	118	249	4786	3.420665714	3.19E-04	3.19E-04	0.002191478
SP_PIR_KEYWORDS	oxidoreductase	29	10.94339623	4.75E-06	254	561	12980	2.641655087	9.68E-04	4.84E-04	0.00596809
GOTERM_BP_FAT	GO:0046164~alcohol catabolic process	8	3.018867925	6.52E-05	161	51	7937	7.733041043	0.071611335	0.036470724	0.104646402
GOTERM_BP_FAT	GO:0019320~hexose catabolic process	7	2.641509434	2.01E-04	161	43	7937	8.025278059	0.204743701	0.073520731	0.322286125
GOTERM_BP_FAT	GO:0006007~glucose catabolic process	7	2.641509434	2.01E-04	161	43	7937	8.025278059	0.204743701	0.073520731	0.322286125
GOTERM_BP_FAT	GO:0006006~glucose metabolic process	8	3.018867925	2.07E-04	161	61	7937	6.465329396	0.210648948	0.057421459	0.332753996
GOTERM_BP_FAT	GO:0046365~monosaccharide catabolic process	7	2.641509434	2.29E-04	161	44	7937	7.842885375	0.229638467	0.050841114	0.366946703
GOTERM_BP_FAT	GO:0019318~hexose metabolic process	9	3.396226415	2.95E-04	161	85	7937	5.219802704	0.285913681	0.054579274	0.473384636
GOTERM_BP_FAT	GO:0006732~coenzyme metabolic process	9	3.396226415	3.75E-04	161	88	7937	5.041854884	0.347812159	0.059233565	0.600461365
GOTERM_BP_FAT	GO:0006091~generation of precursor metabolites and energy	14	5.283018868	4.21E-04	161	217	7937	3.180524945	0.381197443	0.05823182	0.674031406
GOTERM_BP_FAT	GO:0044275~cellular carbohydrate catabolic process	7	2.641509434	5.20E-04	161	51	7937	6.766410912	0.447163147	0.06373311	0.83167139
GOTERM_BP_FAT	GO:0006096~glycolysis	6	2.264150943	6.23E-04	161	35	7937	8.451109139	0.50829285	0.068526182	0.99527816
GOTERM_BP_FAT	GO:0005996~monosaccharide metabolic process	9	3.396226415	7.23E-04	161	97	7937	4.574053916	0.561721886	0.072248215	1.155620585

Architecture BP5

Category	Term	Count	%	PValue	List Total	Pop Hits	Pop Total	Fold Enrichment	Bonferroni	Benjamini	FDR
GOTERM_BP_FAT	GO:0043933~macromolecular complex subunit organization	12	11.32075472	1.98E-05	68	281	7937	4.984509106	0.013978891	0.013978891	0.029916858
GOTERM_BP_FAT	GO:0034621~cellular macromolecular complex subunit organization	9	8.490566038	2.85E-04	68	203	7937	5.174804405	0.183424591	0.096354379	0.429770558
GOTERM_BP_FAT	GO:0007300~ovarian nurse cell to oocyte transport	5	4.716981132	3.05E-04	68	39	7937	14.96417798	0.195242758	0.069845772	0.460618972
SP_PIR_KEYWORDS	proteasome	5	4.716981132	3.69E-04	102	44	12980	14.46078431	0.041926552	0.041926552	0.420363691
GOTERM_BP_FAT	GO:0051493~regulation of cytoskeleton organization	5	4.716981132	7.98E-04	68	50	7937	11.67205882	0.43301783	0.132254539	1.198806208

Architecture WP1

Category	Term	Count	%	PValue	List Total	Pop Hits	Pop Total	Fold Enrichment	Bonferroni	Benjamini	FDR
GOTERM_CC_FAT	GO:0043233~organelle lumen	61	12.68191268	2.74E-08	256	556	4786	2.051104429	1.07E-05	1.07E-05	3.80E-05
GOTERM_CC_FAT	GO:0070013~intracellular organelle lumen	61	12.68191268	2.74E-08	256	556	4786	2.051104429	1.07E-05	1.07E-05	3.80E-05
GOTERM_CC_FAT	GO:0031974~membrane-enclosed lumen	62	12.88981289	2.97E-08	256	571	4786	2.029963879	1.16E-05	5.81E-06	4.13E-05
GOTERM_CC_FAT	GO:0044429~mitochondrial part	50	10.3950104	2.99E-08	256	411	4786	2.274368917	1.17E-05	3.90E-06	4.16E-05
GOTERM_CC_FAT	GO:0031090~organelle membrane	52	10.81081081	3.27E-08	256	438	4786	2.219534817	1.28E-05	3.20E-06	4.55E-05
GOTERM_BP_FAT	GO:0006396~RNA processing	36	7.484407484	3.38E-08	323	312	7937	2.835317933	5.07E-05	5.07E-05	5.61E-05
GOTERM_BP_FAT	GO:0006091~generation of precursor metabolites and energy	28	5.821205821	1.68E-07	323	217	7937	3.170678118	2.53E-04	1.27E-04	2.80E-04
GOTERM_BP_FAT	GO:0006397~mRNA processing	26	5.405405405	2.50E-07	323	194	7937	3.293255881	3.76E-04	1.25E-04	4.15E-04
GOTERM_BP_FAT	GO:0006119~oxidative phosphorylation	21	4.365904366	3.00E-07	323	132	7937	3.909301998	4.51E-04	1.13E-04	4.99E-04
GOTERM_CC_FAT	GO:0005739~mitochondrion	59	12.26611227	4.33E-07	256	571	4786	1.93173982	1.69E-04	3.38E-05	6.02E-04
GOTERM_BP_FAT	GO:0016071~mRNA metabolic process	26	5.405405405	2.47E-06	323	219	7937	2.917313429	0.003711736	7.43E-04	0.004111792
GOTERM_CC_FAT	GO:0030529~ribonucleoprotein complex	43	8.93970894	2.56E-06	256	379	4786	2.121104057	1.00E-03	1.67E-04	0.003555879
GOTERM_BP_FAT	GO:0015980~energy derivation by oxidation of organic compounds	18	3.742203742	2.96E-06	323	114	7937	3.87990875	0.004444145	7.42E-04	0.004924931
GOTERM_CC_FAT	GO:0044455~mitochondrial membrane part	22	4.573804574	6.10E-06	256	134	4786	3.069379664	0.002381769	3.41E-04	0.008478032
GOTERM_CC_FAT	GO:0019866~organelle inner membrane	29	6.029106029	8.64E-06	256	217	4786	2.498451901	0.003371559	4.22E-04	0.012006987
GOTERM_BP_FAT	GO:0022900~electron transport chain	15	3.118503119	1.36E-05	323	90	7937	4.095459236	0.020296236	0.002925005	0.022670813
SP_PIR_KEYWORDS	ribosomal protein	19	3.95010395	1.60E-05	473	157	12980	3.320989483	0.003028416	0.003028416	0.019838131
GOTERM_BP_FAT	GO:0016310~phosphorylation	37	7.692307692	1.88E-05	323	425	7937	2.139275178	0.027840792	0.00352324	0.031216663
GOTERM_BP_FAT	GO:0045333~cellular respiration	16	3.326403326	1.94E-05	323	105	7937	3.744419873	0.028708984	0.00323134	0.032204285
GOTERM_CC_FAT	GO:0031967~organelle envelope	38	7.9002079	2.19E-05	256	344	4786	2.065179869	0.008526377	9.51E-04	0.030440564
GOTERM_CC_FAT	GO:0031975~envelope	38	7.9002079	2.33E-05	256	345	4786	2.059193841	0.009068015	9.11E-04	0.032382819
GOTERM_MF_FAT	GO:0003729~mRNA binding	22	4.573804574	2.50E-05	328	186	7918	2.855297666	0.01247645	0.01247645	0.035967353
GOTERM_CC_FAT	GO:0005761~mitochondrial ribosome	15	3.118503119	2.66E-05	256	74	4786	3.789590372	0.010357227	9.46E-04	0.037009917
GOTERM_CC_FAT	GO:0000313~organellar ribosome	15	3.118503119	2.66E-05	256	74	4786	3.789590372	0.010357227	9.46E-04	0.037009917
GOTERM_BP_FAT	GO:0008380~RNA splicing	19	3.95010395	2.79E-05	323	148	7937	3.154610493	0.04106255	0.004184165	0.046352601
KEGG_PATHWAY	dme00190:Oxidative phosphorylation	22	4.573804574	3.39E-05	126	135	2054	2.656554968	0.002198187	0.002198187	0.034499474
SP_PIR_KEYWORDS	nucleotide-binding	50	10.3950104	3.73E-05	473	743	12980	1.846693167	0.007066438	0.003539483	0.046377639
GOTERM_BP_FAT	GO:0006793~phosphorus metabolic process	43	8.93970894	4.70E-05	323	551	7937	1.917656049	0.068135396	0.006394716	0.077999651
GOTERM_BP_FAT	GO:0006796~phosphate metabolic process	43	8.93970894	4.70E-05	323	551	7937	1.917656049	0.068135396	0.006394716	0.077999651
GOTERM_BP_FAT	GO:0000375~RNA splicing, via transesterification reactions	17	3.534303534	5.05E-05	323	127	7937	3.289266473	0.073124828	0.006308052	0.083931193
GOTERM_MF_FAT	GO:0000166~nucleotide binding	77	16.00831601	6.38E-05	328	1206	7918	1.541292521	0.031562972	0.015908018	0.091853604
GOTERM_MF_FAT	GO:0022890~inorganic cation transmembrane transporter activity	17	3.534303534	6.88E-05	328	128	7918	3.206126143	0.034023948	0.011472429	0.099137198
GOTERM_CC_FAT	GO:0031980~mitochondrial lumen	22	4.573804574	7.19E-05	256	157	4786	2.619725318	0.027732116	0.002340915	0.099943552
GOTERM_CC_FAT	GO:0005759~mitochondrial matrix	22	4.573804574	7.19E-05	256	157	4786	2.619725318	0.027732116	0.002340915	0.099943552
GOTERM_CC_FAT	GO:0005681~spliceosome	14	2.910602911	7.42E-05	256	71	4786	3.686399648	0.02858156	0.002228127	0.103048068
GOTERM_CC_FAT	GO:0005746~mitochondrial respiratory chain	15	3.118503119	8.74E-05	256	82	4786	3.419874238	0.033603311	0.002438513	0.121455033
GOTERM_BP_FAT	GO:0042773~ATP synthesis coupled electron transport	12	2.494802495	1.19E-04	323	71	7937	4.153141761	0.164295287	0.013711282	0.198262629
SP_PIR_KEYWORDS	cytoplasm	33	6.860706861	1.27E-04	473	436	12980	2.077021549	0.023862928	0.008018433	0.157864527
GOTERM_CC_FAT	GO:0005743~mitochondrial inner membrane	25	5.197505198	1.28E-04	256	200	4786	2.336914063	0.048837234	0.00333244	0.177863949
GOTERM_CC_FAT	GO:0070469~respiratory chain	15	3.118503119	1.31E-04	256	85	4786	3.299172794	0.049860635	0.003191559	0.181684618
GOTERM_MF_FAT	GO:0015078~hydrogen ion transmembrane transporter activity	14	2.910602911	1.31E-04	328	95	7918	3.557509628	0.063713269	0.016323679	0.188455855
SP_PIR_KEYWORDS	nucleus	54	11.22661123	1.38E-04	473	869	12980	1.705247946	0.025894895	0.006537554	0.171473095
SP_PIR_KEYWORDS	transferase	54	11.22661123	1.54E-04	473	873	12980	1.697434668	0.028858264	0.005839454	0.191367111
GOTERM_MF_FAT	GO:0015077~monovalent inorganic cation transmembrane transporter activity	14	2.910602911	1.62E-04	328	97	7918	3.484158914	0.078385624	0.016193136	0.233617621
GOTERM_BP_FAT	GO:0000377~RNA splicing, via transesterification reactions with bulged adenosine as nucleophile	16	3.326403326	1.66E-04	323	126	7937	3.120349894	0.220361505	0.017623215	0.274869571
GOTERM_BP_FAT	GO:0000398~nuclear mRNA splicing, via spliceosome	16	3.326403326	1.66E-04	323	126	7937	3.120349894	0.220361505	0.017623215	0.274869571
GOTERM_BP_FAT	GO:0022904~respiratory electron transport chain	12	2.494802495	1.75E-04	323	74	7937	3.984771149	0.23113862	0.017370338	0.290217697
GOTERM_CC_FAT	GO:0005740~mitochondrial envelope	29	6.029106029	2.03E-04	256	258	4786	2.101411095	0.076189071	0.00465079	0.281366224
GOTERM_BP_FAT	GO:0006886~intracellular protein transport	19	3.95010395	2.32E-04	323	174	7937	2.683231913	0.294821211	0.021594912	0.385496836
GOTERM_CC_FAT	GO:0031981~nuclear lumen	38	7.9002079	2.71E-04	256	387	4786	1.835715439	0.100398535	0.005860727	0.375473514
GOTERM_BP_FAT	GO:0006754~ATP biosynthetic process	12	2.494802495	2.82E-04	323	78	7937	3.78042391	0.345122555	0.024592963	0.466976941
GOTERM_MF_FAT	GO:0016887~ATPase activity	30	6.237006237	2.91E-04	328	353	7918	2.051578802	0.136350992	0.02413544	0.41914211
GOTERM_BP_FAT	GO:0034613~cellular protein localization	19	3.95010395	3.30E-04	323	179	7937	2.608281301	0.391422748	0.027213489	0.547643699
GOTERM_BP_FAT	GO:0042775~mitochondrial ATP synthesis coupled electron transport	11	2.286902287	3.32E-04	323	67	7937	4.034332979	0.393171559	0.025947384	0.550808278
GOTERM_BP_FAT	GO:0046034~ATP metabolic process	12	2.494802495	3.53E-04	323	80	7937	3.685913313	0.411662355	0.026174075	0.584831448
GOTERM_MF_FAT	GO:0042625~ATPase activity, coupled to transmembrane movement of ions	12	2.494802495	3.67E-04	328	79	7918	3.666872492	0.168792451	0.026065107	0.528326435
GOTERM_MF_FAT	GO:0042623~ATPase activity, coupled	27	5.613305613	3.76E-04	328	307	7918	2.12308334	0.172550297	0.023397795	0.541240307
GOTERM_CC_FAT	GO:0031966~mitochondrial membrane	27	5.613305613	3.83E-04	256	241	4786	2.094495591	0.13900716	0.007846376	0.530729548
GOTERM_BP_FAT	GO:0022613~ribonucleoprotein complex biogenesis	13	2.702702703	3.88E-04	323	94	7937	3.398359792	0.441956213	0.027394806	0.642926361
GOTERM_BP_FAT	GO:0009206~purine ribonucleoside triphosphate biosynthetic process	12	2.494802495	4.39E-04	323	82	7937	3.596012988	0.482913086	0.029534365	0.72663636
GOTERM_BP_FAT	GO:0009145~purine nucleoside triphosphate biosynthetic process	12	2.494802495	4.39E-04	323	82	7937	3.596012988	0.482913086	0.029534365	0.72663636
GOTERM_BP_FAT	GO:0006605~protein targeting	14	2.910602911	4.40E-04	323	109	7937	3.156133723	0.484006598	0.028358027	0.728960183
GOTERM_BP_FAT	GO:0009142~nucleoside triphosphate biosynthetic process	12	2.494802495	4.88E-04	323	83	7937	3.55268753	0.519713163	0.030095007	0.807644617
GOTERM_BP_FAT	GO:0009201~ribonucleoside triphosphate biosynthetic process	12	2.494802495	4.88E-04	323	83	7937	3.55268753	0.519713163	0.030095007	0.807644617
GOTERM_BP_FAT	GO:0034220~ion transmembrane transport	10	2.079002079	4.89E-04	323	58	7937	4.236681969	0.520918466	0.029006378	0.810400554
GOTERM_BP_FAT	GO:0016568~chromatin modification	14	2.910602911	5.73E-04	323	112	7937	3.071594427	0.577703099	0.032612017	0.948677008
GOTERM_MF_FAT	GO:0032555~purine ribonucleotide binding	59	12.26611227	6.07E-04	328	923	7918	1.543092514	0.263125028	0.033357294	0.871070385
GOTERM_MF_FAT	GO:0032553~ribonucleotide binding	59	12.26611227	6.07E-04	328	923	7918	1.543092514	0.263125028	0.033357294	0.871070385
GOTERM_BP_FAT	GO:0000278~mitotic cell cycle	29	6.029106029	6.15E-04	323	358	7937	1.990530467	0.603419523	0.033674628	1.017467715
SP_PIR_KEYWORDS	phosphoprotein	49	10.18711019	6.35E-04	473	815	12980	1.649878721	0.113667443	0.019909641	0.786196833
GOTERM_MF_FAT	GO:0017076~purine nucleotide binding	62	12.88981289	7.06E-04	328	989	7918	1.513341883	0.29895749	0.034895289	1.012560155
GOTERM_BP_FAT	GO:0009205~purine ribonucleoside triphosphate metabolic process	12	2.494802495	7.33E-04	323	87	7937	3.389345575	0.667644204	0.038577233	1.210646854
GOTERM_BP_FAT	GO:0009144~purine nucleoside triphosphate metabolic process	12	2.494802495	7.33E-04	323	87	7937	3.389345575	0.667644204	0.038577233	1.210646854
GOTERM_CC_FAT	GO:0005654~nucleoplasm	28	5.821205821	7.35E-04	256	265	4786	1.975353774	0.24980269	0.014268182	1.016703991
GOTERM_MF_FAT	GO:0015399~primary active transmembrane transporter activity	17	3.534303534	7.35E-04	328	157	7918	2.61391176	0.309136186	0.033060474	1.054034654
GOTERM_MF_FAT	GO:0015405~P-P-bond-hydrolysis-driven transmembrane transporter activity	17	3.534303534	7.35E-04	328	157	7918	2.61391176	0.309136186	0.033060474	1.054034654
GOTERM_CC_FAT	GO:0000314~organellar small ribosomal subunit	8	1.663201663	7.94E-04	256	30	4786	4.985416667	0.266861459	0.014673216	1.097620633
GOTERM_CC_FAT	GO:0005763~mitochondrial small ribosomal subunit	8	1.663201663	7.94E-04	256	30	4786	4.985416667	0.266861459	0.014673216	1.097620633
GOTERM_BP_FAT	GO:0009199~ribonucleoside triphosphate metabolic process	12	2.494802495	8.08E-04	323	88	7937	3.350830284	0.703109116	0.041010869	1.333833457
GOTERM_BP_FAT	GO:0015031~protein transport	24	4.98960499	8.83E-04	323	278	7937	2.121388957	0.735015216	0.043303839	1.4577936
GOTERM_BP_FAT	GO:0009141~nucleoside triphosphate metabolic process	12	2.494802495	8.89E-04	323	89	7937	3.313180506	0.737279377	0.042201825	1.467143477
GOTERM_BP_FAT	GO:0070727~cellular macromolecule localization	22	4.573804574	8.89E-04	323	244	7937	2.215576308	0.737382454	0.040922137	1.46757103
GOTERM_BP_FAT	GO:0006612~protein targeting to membrane	7	1.455301455	9.16E-04	323	29	7937	5.931354756	0.747740291	0.040877281	1.511403135
GOTERM_MF_FAT	GO:0015662~ATPase activity, coupled to transmembrane movement of ions, phosphorylative mechanism	11	2.286902287	9.56E-04	328	75	7918	3.540569106	0.381788159	0.039284546	1.368547394

Architecture WP2

Category	Term	Count	%	PValue	List Total	Pop Hits	Pop Total	Fold Enrichment	Bonferroni	Benjamini	FDR
NONE											

Architecture WP3

Category	Term	Count	%	PValue	List Total	Pop Hits	Pop Total	Fold Enrichment	Bonferroni	Benjamini	FDR
SP_PIR_KEYWORDS	cytoplasm	39	9.397590361	7.99E-09	403	436	12980	2.881029891	1.78E-06	1.78E-06	1.02E-05
GOTERM_BP_FAT	GO:0007010~cytoskeleton organization	42	10.12048193	9.01E-09	268	465	7937	2.67496389	1.17E-05	1.17E-05	1.47E-05
SP_PIR_KEYWORDS	phosphoprotein	52	12.53012048	1.14E-06	403	815	12980	2.055016822	2.55E-04	1.27E-04	0.001456437
SP_PIR_KEYWORDS	ribosomal protein	19	4.578313253	1.73E-06	403	157	12980	3.897836292	3.86E-04	1.29E-04	0.002208205
GOTERM_BP_FAT	GO:0000226~microtubule cytoskeleton organization	28	6.746987952	2.30E-06	268	298	7937	2.782680557	0.002986581	0.001494407	0.003759149
GOTERM_CC_FAT	GO:0043228~non-membrane-bounded organelle	69	16.62650602	2.31E-06	206	943	4786	1.69997632	8.04E-04	8.04E-04	0.003160095
GOTERM_CC_FAT	GO:0043232~intracellular non-membrane-bounded organelle	69	16.62650602	2.31E-06	206	943	4786	1.69997632	8.04E-04	8.04E-04	0.003160095
GOTERM_BP_FAT	GO:0008104~protein localization	33	7.951807229	2.48E-06	268	392	7937	2.493156031	0.003210219	0.00107122	0.004041085
GOTERM_BP_FAT	GO:0007017~microtubule-based process	33	7.951807229	5.30E-06	268	406	7937	2.407185133	0.006858026	0.001718933	0.008648643
SP_PIR_KEYWORDS	ribonucleoprotein	16	3.855421687	1.28E-05	403	131	12980	3.933854867	0.002859849	7.16E-04	0.016382404
SP_PIR_KEYWORDS	cytoskeleton	13	3.13253012	1.82E-05	403	89	12980	4.704603117	0.004048161	8.11E-04	0.023202579
GOTERM_MF_FAT	GO:0003735~structural constituent of ribosome	19	4.578313253	3.53E-05	272	178	7918	3.107278586	0.014120519	0.014120519	0.049261629
GOTERM_MF_FAT	GO:0000166~nucleotide binding	67	16.14457831	4.31E-05	272	1206	7918	1.617238562	0.017212895	0.008643805	0.060140743
GOTERM_BP_FAT	GO:0015031~protein transport	24	5.78313253	5.71E-05	268	278	7937	2.556748631	0.071488274	0.014724965	0.093177982
GOTERM_BP_FAT	GO:0043484~regulation of RNA splicing	11	2.65060241	6.26E-05	268	66	7937	4.935945274	0.078150631	0.013470688	0.102219742
GOTERM_BP_FAT	GO:0000278~mitotic cell cycle	28	6.746987952	6.52E-05	268	358	7937	2.316309514	0.081260131	0.012034465	0.10646188
GOTERM_BP_FAT	GO:0070727~cellular macromolecule localization	22	5.301204819	6.90E-05	268	244	7937	2.670265476	0.085738193	0.011142249	0.112596057
GOTERM_BP_FAT	GO:0007049~cell cycle	40	9.638554217	8.05E-05	268	616	7937	1.923095561	0.09927138	0.011549593	0.131316149
GOTERM_BP_FAT	GO:0045184~establishment of protein localization	24	5.78313253	8.44E-05	268	285	7937	2.493951296	0.103897644	0.010910112	0.137779288
SP_PIR_KEYWORDS	alternative splicing	38	9.156626506	8.74E-05	403	616	12980	1.986884084	0.019305515	0.003243778	0.111458668
SP_PIR_KEYWORDS	acetylation	7	1.686746988	9.13E-05	403	25	12980	9.018362283	0.02015224	0.00290407	0.116394376
SP_PIR_KEYWORDS	nucleus	48	11.56626506	1.27E-04	403	869	12980	1.779062098	0.027964147	0.003539046	0.162121993
SP_PIR_KEYWORDS	cell cycle	12	2.891566265	1.82E-04	403	96	12980	4.026054591	0.039763791	0.004498294	0.231853333
GOTERM_CC_FAT	GO:0033279~ribosomal subunit	19	4.578313253	1.91E-04	206	164	4786	2.691629174	0.064179455	0.032621819	0.260129376
GOTERM_BP_FAT	GO:0050684~regulation of mRNA processing	10	2.409638554	2.32E-04	268	63	7937	4.700900261	0.260280317	0.027035452	0.378195047
GOTERM_BP_FAT	GO:0048024~regulation of nuclear mRNA splicing, via spliceosome	10	2.409638554	2.32E-04	268	63	7937	4.700900261	0.260280317	0.027035452	0.378195047
GOTERM_BP_FAT	GO:0000022~mitotic spindle elongation	11	2.65060241	2.62E-04	268	78	7937	4.176569078	0.288464152	0.027962398	0.426820465
GOTERM_CC_FAT	GO:0015629~actin cytoskeleton	13	3.13253012	2.87E-04	206	87	4786	3.471599152	0.095155727	0.032781457	0.391876763
GOTERM_BP_FAT	GO:0051231~spindle elongation	11	2.65060241	2.91E-04	268	79	7937	4.123701115	0.314986582	0.028681932	0.474348748
GOTERM_CC_FAT	GO:0005840~ribosome	20	4.819277108	3.24E-04	206	186	4786	2.498173087	0.106689527	0.027811216	0.442041832
GOTERM_CC_FAT	GO:0005811~lipid particle	24	5.78313253	3.41E-04	206	249	4786	2.239326237	0.111771942	0.023426585	0.464345086
GOTERM_BP_FAT	GO:0051276~chromosome organization	22	5.301204819	5.13E-04	268	282	7937	2.310442469	0.486837658	0.04653685	0.835000235
GOTERM_BP_FAT	GO:0007051~spindle organization	19	4.578313253	5.57E-04	268	225	7937	2.500878939	0.515004978	0.047095995	0.905335046
GOTERM_CC_FAT	GO:0030133~transport vesicle	6	1.445783133	5.57E-04	206	17	4786	8.19988578	0.176311308	0.031810168	0.758752191
GOTERM_BP_FAT	GO:0016192~vesicle-mediated transport	27	6.506024096	6.58E-04	268	391	7937	2.045071955	0.57475418	0.05204002	1.068939972
GOTERM_BP_FAT	GO:0022402~cell cycle process	34	8.192771084	7.08E-04	268	547	7937	1.840827853	0.601281072	0.052651406	1.148995049
GOTERM_BP_FAT	GO:0006793~phosphorus metabolic process	34	8.192771084	8.04E-04	268	551	7937	1.827464312	0.648111153	0.056373116	1.30409819
GOTERM_BP_FAT	GO:0006796~phosphate metabolic process	34	8.192771084	8.04E-04	268	551	7937	1.827464312	0.648111153	0.056373116	1.30409819
GOTERM_BP_FAT	GO:0007052~mitotic spindle organization	17	4.096385542	8.09E-04	268	194	7937	2.595187721	0.650600736	0.053840585	1.31290494
GOTERM_MF_FAT	GO:0005198~structural molecule activity	30	7.228915663	8.56E-04	272	456	7918	1.915150929	0.291801901	0.108643193	1.188354431
SP_PIR_KEYWORDS	nucleotide-binding	40	9.638554217	8.68E-04	403	743	12980	1.733966984	0.176127241	0.01918745	1.102202356
SP_PIR_KEYWORDS	mitosis	8	1.927710843	9.26E-04	403	51	12980	5.0523038	0.186662694	0.018607374	1.174990373

Architecture WP4

Category	Term	Count	%	PValue	List Total	Pop Hits	Pop Total	Fold Enrichment	Bonferroni	Benjamini	FDR
GOTERM_BP_FAT	GO:0008104~protein localization	38	7.142857143	6.56E-07	316	392	7937	2.434819814	9.60E-04	9.60E-04	0.001086507
GOTERM_BP_FAT	GO:0045184~establishment of protein localization	30	5.639097744	2.61E-06	316	285	7937	2.643904064	0.003814338	0.001908991	0.004321848
SP_PIR_KEYWORDS	cytoplasm	39	7.330827068	4.40E-06	516	436	12980	2.250106678	9.54E-04	9.54E-04	0.005587749
GOTERM_BP_FAT	GO:0015031~protein transport	29	5.45112782	4.77E-06	316	278	7937	2.620127948	0.006968063	0.002328103	0.007907561
GOTERM_CC_FAT	GO:0005811~lipid particle	30	5.639097744	6.95E-06	233	249	4786	2.474791871	0.002395193	0.002395193	0.009485833
GOTERM_BP_FAT	GO:0016044~membrane organization	31	5.827067669	8.68E-06	316	319	7937	2.440845601	0.012634657	0.003173741	0.01437873
GOTERM_CC_FAT	GO:0044429~mitochondrial part	41	7.706766917	1.06E-05	233	411	4786	2.049079498	0.003639437	0.001821377	0.01442213
GOTERM_BP_FAT	GO:0007010~cytoskeleton organization	38	7.142857143	3.51E-05	316	465	7937	2.052579284	0.050106884	0.010228492	0.058118732
SP_PIR_KEYWORDS	mitochondrion	21	3.947368421	4.58E-05	516	185	12980	2.855436832	0.009899034	0.004961827	0.058211937
GOTERM_CC_FAT	GO:0031090~organelle membrane	41	7.706766917	4.78E-05	233	438	4786	1.922766379	0.016348225	0.005479377	0.065183609
GOTERM_BP_FAT	GO:0044265~cellular macromolecule catabolic process	23	4.323308271	8.03E-05	316	225	7937	2.567524613	0.111003856	0.019419364	0.132977715
GOTERM_CC_FAT	GO:0031967~organelle envelope	34	6.390977444	9.29E-05	233	344	4786	2.030192634	0.031553383	0.007983441	0.126750326
GOTERM_BP_FAT	GO:0009057~macromolecule catabolic process	26	4.887218045	9.81E-05	316	277	7937	2.357560664	0.13387134	0.020322348	0.162405204
GOTERM_CC_FAT	GO:0031975~envelope	34	6.390977444	9.84E-05	233	345	4786	2.024308018	0.033376373	0.006766221	0.134193971
GOTERM_BP_FAT	GO:0046907~intracellular transport	28	5.263157895	1.11E-04	316	313	7937	2.246896105	0.150490668	0.020180633	0.18427819
GOTERM_CC_FAT	GO:0005739~mitochondrion	48	9.022556391	1.33E-04	233	571	4786	1.726719933	0.04492496	0.007631624	0.181664859
SP_PIR_KEYWORDS	protein transport	14	2.631578947	1.73E-04	516	101	12980	3.486837056	0.036926279	0.012463452	0.219983059
SP_PIR_KEYWORDS	cytoplasmic vesicle	6	1.127819549	2.08E-04	516	15	12980	10.0620155	0.044137446	0.011221847	0.263867668
SP_PIR_KEYWORDS	nucleus	57	10.71428571	2.08E-04	516	869	12980	1.649985281	0.044181395	0.008996711	0.264136085
GOTERM_BP_FAT	GO:0034622~cellular macromolecular complex assembly	18	3.383458647	2.80E-04	316	165	7937	2.74004603	0.336666286	0.044584127	0.463139071
GOTERM_BP_FAT	GO:0051603~proteolysis involved in cellular protein catabolic process	19	3.571428571	3.32E-04	316	183	7937	2.607785156	0.385216642	0.047484152	0.548663116
GOTERM_BP_FAT	GO:0044257~cellular protein catabolic process	19	3.571428571	3.32E-04	316	183	7937	2.607785156	0.385216642	0.047484152	0.548663116
GOTERM_BP_FAT	GO:0030163~protein catabolic process	20	3.759398496	3.56E-04	316	200	7937	2.511708861	0.406326482	0.046296393	0.587953199
SP_PIR_KEYWORDS	phosphoprotein	53	9.962406015	4.50E-04	516	815	12980	1.635849146	0.093076501	0.016151014	0.570201141
SP_PIR_KEYWORDS	transit peptide	14	2.631578947	5.76E-04	516	114	12980	3.089215286	0.117606427	0.017715081	0.72965001
GOTERM_CC_FAT	GO:0005740~mitochondrial envelope	26	4.887218045	5.97E-04	233	258	4786	2.070000333	0.186198107	0.029005083	0.811735107
SMART	SM00504:Ubox	4	0.751879699	6.03E-04	195	5	4824	19.79076923	0.083232511	0.083232511	0.713011587
GOTERM_BP_FAT	GO:0016192~vesicle-mediated transport	30	5.639097744	8.10E-04	316	391	7937	1.927142348	0.69504421	0.094226229	1.33408335
INTERPRO	IPR003613:U box domain	4	0.751879699	8.69E-04	464	5	10196	17.57931034	0.503234733	0.503234733	1.326312751
SP_PIR_KEYWORDS	mitochondrion inner membrane	9	1.691729323	9.39E-04	516	52	12980	4.353756708	0.184379085	0.025153932	1.185811148

Architecture WP5

Category	Term	Count	%	PValue	List Total	Pop Hits	Pop Total	Fold Enrichment	Bonferroni	Benjamini	FDR
GOTERM_CC_FAT	GO:0005739~mitochondrion	64	13.19587629	2.35E-11	228	571	4786	2.35278213	6.71E-09	6.71E-09	3.11E-08
GOTERM_CC_FAT	GO:0044429~mitochondrial part	51	10.51546392	1.49E-10	228	411	4786	2.604750928	4.27E-08	2.14E-08	1.98E-07
SP_PIR_KEYWORDS	ribosomal protein	24	4.948453608	1.14E-08	473	157	12980	4.194934084	2.36E-06	2.36E-06	1.44E-05
GOTERM_MF_FAT	GO:0003735~structural constituent of ribosome	25	5.154639175	8.96E-08	310	178	7918	3.587350489	4.15E-05	4.15E-05	1.28E-04
GOTERM_CC_FAT	GO:0031980~mitochondrial lumen	24	4.948453608	9.35E-07	228	157	4786	3.208850151	2.67E-04	8.91E-05	0.001240237
GOTERM_CC_FAT	GO:0005759~mitochondrial matrix	24	4.948453608	9.35E-07	228	157	4786	3.208850151	2.67E-04	8.91E-05	0.001240237
SP_PIR_KEYWORDS	mitochondrion	23	4.742268041	9.96E-07	473	185	12980	3.411690761	2.05E-04	1.03E-04	0.001254209
GOTERM_CC_FAT	GO:0005761~mitochondrial ribosome	16	3.298969072	1.30E-06	228	74	4786	4.538643907	3.73E-04	9.33E-05	0.001730801
GOTERM_CC_FAT	GO:0000313~organellar ribosome	16	3.298969072	1.30E-06	228	74	4786	4.538643907	3.73E-04	9.33E-05	0.001730801
GOTERM_CC_FAT	GO:0005840~ribosome	26	5.360824742	1.60E-06	228	186	4786	2.934257687	4.57E-04	9.15E-05	0.002122433
GOTERM_BP_FAT	GO:0044265~cellular macromolecule catabolic process	26	5.360824742	1.61E-06	307	225	7937	2.987506334	0.002082928	0.002082928	0.002627664
GOTERM_CC_FAT	GO:0033279~ribosomal subunit	24	4.948453608	2.04E-06	228	164	4786	3.071887035	5.84E-04	9.74E-05	0.002709833
SP_PIR_KEYWORDS	transit peptide	17	3.505154639	3.51E-06	473	114	12980	4.092207262	7.22E-04	2.41E-04	0.004418027
