# Supplementary material for: Multiple novel promoter-architectures revealed by decoding the hidden heterogeneity within the genome
Source: Nucleic Acids Res. 2014 Oct 17;42(20):12388–403. doi: 10.1093/nar/gku924 (PMC4227772; doi:10.1093/nar/gku924)
Supplement: SUPPLEMENTARY DATA [file supp_gku924_nar-02085-n-2014-File005.zip › TableS2.pdf]

Architecture A1

Category	Term	Count	%	PValue	List Total	Pop Hits	Pop Total	Fold Enrichment	Bonferroni	Benjamini	FDR
GOTERM_CC_FAT	GO:0005576~extracellular region	83	25.15151515	1.53E-13	234	2010	12782	2.255615087	4.64E-11	4.64E-11	2.05E-10
SP_PIR_KEYWORDS	Secreted	70	21.21212121	1.90E-12	322	1689	19235	2.475738152	7.77E-10	7.77E-10	2.65E-09
GOTERM_BP_FAT	GO:0030216~keratinocyte differentiation	14	4.242424242	1.84E-10	251	66	13528	11.43257274	3.40E-07	3.40E-07	3.13E-07
GOTERM_BP_FAT	GO:0009913~epidermal cell differentiation	14	4.242424242	5.78E-10	251	72	13528	10.47985834	1.07E-06	5.35E-07	9.85E-07
GOTERM_BP_FAT	GO:0031424~keratinization	11	3.333333333	4.36E-09	251	43	13528	13.78745483	8.06E-06	2.69E-06	7.42E-06
INTERPRO	IPR003267:Small proline-rich	9	2.727272727	5.91E-09	288	25	16659	20.82375	3.59E-06	3.59E-06	8.74E-06
GOTERM_BP_FAT	GO:0008544~epidermis development	19	5.757575758	8.62E-09	251	184	13528	5.565390611	1.60E-05	3.99E-06	1.47E-05
SP_PIR_KEYWORDS	keratinization	10	3.03030303	1.56E-08	322	40	19235	14.93400621	6.39E-06	3.19E-06	2.18E-05
GOTERM_BP_FAT	GO:0007398~ectoderm development	19	5.757575758	2.95E-08	251	199	13528	5.145888807	5.46E-05	1.09E-05	5.03E-05
GOTERM_CC_FAT	GO:0001533~cornified envelope	8	2.424242424	8.26E-08	234	22	12782	19.86324786	2.50E-05	1.25E-05	1.11E-04
GOTERM_MF_FAT	GO:0005198~structural molecule activity	33	10	1.35E-07	236	634	12983	2.863437684	6.46E-05	6.46E-05	1.93E-04
SP_PIR_KEYWORDS	signal	90	27.27272727	6.86E-07	322	3250	19235	1.65422838	2.81E-04	9.38E-05	9.60E-04
UP_SEQ_FEATURE	signal peptide	90	27.27272727	9.05E-07	322	3250	19113	1.643736264	9.79E-04	9.79E-04	0.001444616
GOTERM_BP_FAT	GO:0030855~epithelial cell differentiation	14	4.242424242	1.50E-06	251	137	13528	5.50766278	0.002777033	4.63E-04	0.002560898
GOTERM_CC_FAT	GO:0045095~keratin filament	11	3.333333333	4.35E-06	234	88	12782	6.827991453	0.001316005	4.39E-04	0.005816361
GOTERM_BP_FAT	GO:0060429~epithelium development	17	5.151515152	4.94E-06	251	227	13528	4.036295347	0.009098552	0.00130489	0.008416864
SP_PIR_KEYWORDS	keratin	13	3.939393939	5.51E-06	322	144	19235	5.392835576	0.002257582	5.65E-04	0.007716227
GOTERM_CC_FAT	GO:0031983~vesicle lumen	8	2.424242424	1.79E-05	234	46	12782	9.499814195	0.005395786	0.001351684	0.023894479
GOTERM_BP_FAT	GO:0030005~cellular di-, tri-valent inorganic cation homeostasis	16	4.848484848	2.11E-05	251	227	13528	3.798866209	0.038370836	0.004878858	0.036025048
GOTERM_CC_FAT	GO:0044430~cytoskeletal part	37	11.21212121	2.17E-05	234	952	12782	2.122988939	0.006552317	0.001313912	0.02903214
GOTERM_BP_FAT	GO:0055080~cation homeostasis	18	5.454545455	2.36E-05	251	286	13528	3.392082022	0.042678106	0.004834453	0.040157583
GOTERM_BP_FAT	GO:0050801~ion homeostasis	22	6.666666667	2.41E-05	251	409	13528	2.899073632	0.043596593	0.004447628	0.041041191
GOTERM_BP_FAT	GO:0048878~chemical homeostasis	25	7.575757576	2.77E-05	251	512	13528	2.631660857	0.049899856	0.004642634	0.047127878
GOTERM_CC_FAT	GO:0005882~intermediate filament	14	4.242424242	3.05E-05	234	183	12782	4.178880015	0.009191938	0.00153789	0.040779572
GOTERM_BP_FAT	GO:0006874~cellular calcium ion homeostasis	14	4.242424242	3.55E-05	251	183	13528	4.123222955	0.06356678	0.005458139	0.060463806
GOTERM_CC_FAT	GO:0045111~intermediate filament cytoskeleton	14	4.242424242	3.82E-05	234	187	12782	4.089492207	0.011516095	0.001653334	0.05114785
GOTERM_BP_FAT	GO:0055066~di-, tri-valent inorganic cation homeostasis	16	4.848484848	3.84E-05	251	239	13528	3.608128157	0.068539642	0.005446777	0.065364133
GOTERM_BP_FAT	GO:0055074~calcium ion homeostasis	14	4.242424242	4.70E-05	251	188	13528	4.01356277	0.083282761	0.006191908	0.080046026
GOTERM_CC_FAT	GO:0005615~extracellular space	29	8.787878788	5.17E-05	234	685	12782	2.31254601	0.01553728	0.001955492	0.069141884
GOTERM_BP_FAT	GO:0006873~cellular ion homeostasis	20	6.060606061	6.80E-05	251	374	13528	2.882161195	0.118149598	0.008347157	0.115720529
UP_SEQ_FEATURE	repeat:3	14	4.242424242	7.10E-05	322	214	19113	3.88317757	0.073908169	0.037663349	0.113181255
GOTERM_BP_FAT	GO:0006875~cellular metal ion homeostasis	14	4.242424242	7.22E-05	251	196	13528	3.849743882	0.125049656	0.008314501	0.122945846
GOTERM_CC_FAT	GO:0005856~cytoskeleton	46	13.93939394	7.42E-05	234	1381	12782	1.819479258	0.022240565	0.002495948	0.09929455
UP_SEQ_FEATURE	repeat:2	15	4.545454545	7.60E-05	322	246	19113	3.619337979	0.078971228	0.027048783	0.121257344
GOTERM_BP_FAT	GO:0030003~cellular cation homeostasis	16	4.848484848	7.71E-05	251	254	13528	3.395049722	0.132929803	0.008355214	0.131266831
GOTERM_BP_FAT	GO:0055082~cellular chemical homeostasis	20	6.060606061	8.39E-05	251	380	13528	2.836653386	0.143802137	0.00858812	0.14287124
GOTERM_CC_FAT	GO:0030141~secretory granule	13	3.939393939	1.14E-04	234	180	12782	3.945061728	0.033815868	0.003434175	0.151830751
GOTERM_BP_FAT	GO:0055065~metal ion homeostasis	14	4.242424242	1.14E-04	251	205	13528	3.680730736	0.189917942	0.011024034	0.193771989
SP_PIR_KEYWORDS	Pyrrolidone carboxylic acid	8	2.424242424	1.25E-04	322	67	19235	7.132659683	0.049777977	0.010159958	0.174175279
GOTERM_CC_FAT	GO:0060205~cytoplasmic membrane-bounded vesicle lumen	7	2.121212121	1.36E-04	234	44	12782	8.69017094	0.040379457	0.00374002	0.181888469
GOTERM_CC_FAT	GO:0044421~extracellular region part	35	10.60606061	1.37E-04	234	960	12782	1.991497507	0.040772521	0.003462909	0.183694719
GOTERM_BP_FAT	GO:0006955~immune response	28	8.484848485	1.89E-04	251	690	13528	2.187100872	0.295363255	0.017351344	0.321863438
SP_PIR_KEYWORDS	methylation	14	4.242424242	2.27E-04	322	242	19235	3.45580309	0.089034604	0.015421578	0.317868048
SP_PIR_KEYWORDS	cadmium	4	1.212121212	2.42E-04	322	8	19235	29.86801242	0.094582486	0.014093895	0.338655948
UP_SEQ_FEATURE	repeat:1	14	4.242424242	2.51E-04	322	243	19113	3.419753086	0.237640314	0.065584615	0.399394094
SP_PIR_KEYWORDS	acetylated amino end	9	2.727272727	2.71E-04	322	100	19235	5.376242236	0.105211235	0.013799848	0.378827778
GOTERM_BP_FAT	GO:0042592~homeostatic process	29	8.787878788	3.18E-04	251	751	13528	2.081219728	0.444338309	0.027592909	0.539656145
UP_SEQ_FEATURE	region of interest:Beta	4	1.212121212	5.16E-04	322	10	19113	23.74285714	0.427887582	0.105673139	0.820229177
UP_SEQ_FEATURE	region of interest:Alpha	4	1.212121212	5.16E-04	322	10	19113	23.74285714	0.427887582	0.105673139	0.820229177
GOTERM_MF_FAT	GO:0046870~cadmium ion binding	4	1.212121212	6.40E-04	236	10	12983	22.00508475	0.264409075	0.142334025	0.91179867
UP_SEQ_FEATURE	disulfide bond	70	21.21212121	6.77E-04	322	2819	19113	1.473926924	0.519324852	0.114934773	1.074639798
UP_SEQ_FEATURE	metal ion-binding site:Divalent metal cation; cluster B	4	1.212121212	7.01E-04	322	11	19113	21.58441558	0.531570048	0.102676097	1.112283487
UP_SEQ_FEATURE	metal ion-binding site:Divalent metal cation; cluster A	4	1.212121212	7.01E-04	322	11	19113	21.58441558	0.531570048	0.102676097	1.112283487
UP_SEQ_FEATURE	region of interest:Coil 2	7	2.121212121	7.08E-04	322	64	19113	6.4921875	0.535411152	0.091377268	1.124291625
UP_SEQ_FEATURE	region of interest:Linker 12	7	2.121212121	7.08E-04	322	64	19113	6.4921875	0.535411152	0.091377268	1.124291625
SP_PIR_KEYWORDS	heparin-binding	7	2.121212121	7.45E-04	322	65	19235	6.433110368	0.263148969	0.033360783	1.037173006
INTERPRO	IPR018064:Metallothionein, vertebrate, metal binding site	4	1.212121212	7.54E-04	288	11	16659	21.03409091	0.367667395	0.204806561	1.10919751
UP_SEQ_FEATURE	region of interest:3 X 9 AA tandem repeats of P-K-C-P-[EQ]- P-C-P-P	3	0.909090909	8.34E-04	322	3	19113	59.35714286	0.594649978	0.09546458	1.323008525
GOTERM_CC_FAT	GO:0031093~platelet alpha granule lumen	6	1.818181818	8.46E-04	234	41	12782	7.993746091	0.226222412	0.019535189	1.126421267
SP_PIR_KEYWORDS	metal-thiolate cluster	4	1.212121212	9.06E-04	322	12	19235	19.91200828	0.310338553	0.036473652	1.260544653
GOTERM_CC_FAT	GO:0000786~nucleosome	7	2.121212121	9.82E-04	234	63	12782	6.069325736	0.257444738	0.021036857	1.306129444
INTERPRO	IPR000006:Metallothionein, vertebrate	4	1.212121212	9.92E-04	288	12	16659	19.28125	0.453066539	0.182204284	1.457745999
INTERPRO	IPR003019:Metallothionein superfamily, eukaryotic	4	1.212121212	9.92E-04	288	12	16659	19.28125	0.453066539	0.182204284	1.457745999

Architecture A2

Category	Term	Count	%	PValue	List Total	Pop Hits	Pop Total	Fold Enrichment	Bonferroni	Benjamini	FDR
SP_PIR_KEYWORDS	disease mutation	57	23.26530612	1.45E-12	245	1591	19235	2.812747726	6.20E-10	6.20E-10	2.04E-09
SP_PIR_KEYWORDS	Secreted	54	22.04081633	4.69E-10	245	1689	19235	2.510095335	2.01E-07	1.00E-07	6.61E-07
SP_PIR_KEYWORDS	disulfide bond	70	28.57142857	1.11E-07	245	2924	19235	1.87951925	4.74E-05	1.58E-05	1.56E-04
SP_PIR_KEYWORDS	signal	75	30.6122449	1.36E-07	245	3250	19235	1.81177394	5.84E-05	1.46E-05	1.92E-04
UP_SEQ_FEATURE	signal peptide	75	30.6122449	1.47E-07	244	3250	19113	1.807660782	1.90E-04	1.90E-04	2.41E-04
GOTERM_CC_FAT	GO:0044421~extracellular region part	36	14.69387755	2.97E-07	186	960	12782	2.577016129	7.82E-05	7.82E-05	3.89E-04
SP_PIR_KEYWORDS	cleavage on pair of basic residues	17	6.93877551	4.01E-07	245	271	19235	4.924994352	1.72E-04	3.43E-05	5.65E-04
GOTERM_CC_FAT	GO:0005615~extracellular space	29	11.83673469	5.86E-07	186	685	12782	2.909332078	1.54E-04	7.71E-05	7.67E-04
SP_PIR_KEYWORDS	keratinization	8	3.265306122	6.33E-07	245	40	19235	15.70204082	2.71E-04	4.51E-05	8.91E-04
UP_SEQ_FEATURE	disulfide bond	66	26.93877551	7.46E-07	244	2819	19113	1.833951698	9.62E-04	4.81E-04	0.001216715
GOTERM_BP_FAT	GO:0008544~epidermis development	15	6.12244898	8.46E-07	207	184	13528	5.327662256	0.001611926	0.001611926	0.001447031
GOTERM_BP_FAT	GO:0007398~ectoderm development	15	6.12244898	2.16E-06	207	199	13528	4.926079674	0.004107637	0.002055932	0.003692013
GOTERM_CC_FAT	GO:0005576~extracellular region	55	22.44897959	2.37E-06	186	2010	12782	1.880409779	6.23E-04	2.08E-04	0.003103795
UP_SEQ_FEATURE	region of interest:Coil 1A	9	3.673469388	2.91E-06	244	70	19113	10.07125293	0.003748925	0.001251207	0.00474934
UP_SEQ_FEATURE	region of interest:Coil 1B	9	3.673469388	2.91E-06	244	70	19113	10.07125293	0.003748925	0.001251207	0.00474934
UP_SEQ_FEATURE	region of interest:Linker 1	9	3.673469388	2.91E-06	244	70	19113	10.07125293	0.003748925	0.001251207	0.00474934
UP_SEQ_FEATURE	region of interest:Rod	9	3.673469388	3.25E-06	244	71	19113	9.929404295	0.004178171	0.001046183	0.005294258
GOTERM_BP_FAT	GO:0031424~keratinization	8	3.265306122	3.47E-06	207	43	13528	12.15863386	0.006593372	0.002202639	0.00593357
UP_SEQ_FEATURE	region of interest:Head	9	3.673469388	4.01E-06	244	73	19113	9.657365821	0.005162329	0.001034604	0.006544498
UP_SEQ_FEATURE	region of interest:Tail	9	3.673469388	4.93E-06	244	75	19113	9.399836066	0.006335459	0.001058708	0.008036399
INTERPRO	IPR018039:Intermediate filament protein, conserved site	9	3.673469388	5.75E-06	227	72	16659	9.17345815	0.002985905	0.002985905	0.008326581
INTERPRO	IPR016044:Filament	9	3.673469388	5.75E-06	227	72	16659	9.17345815	0.002985905	0.002985905	0.008326581
SP_PIR_KEYWORDS	Intermediate filament	9	3.673469388	6.52E-06	245	78	19235	9.058869702	0.002787401	3.99E-04	0.00918563
UP_SEQ_FEATURE	region of interest:Coil 2	8	3.265306122	1.65E-05	244	64	19113	9.791495902	0.021075203	0.003038297	0.026930964
UP_SEQ_FEATURE	region of interest:Linker 12	8	3.265306122	1.65E-05	244	64	19113	9.791495902	0.021075203	0.003038297	0.026930964
GOTERM_BP_FAT	GO:0003013~circulatory system process	13	5.306122449	2.79E-05	207	186	13528	4.567658823	0.051820622	0.013214802	0.047718654
GOTERM_BP_FAT	GO:0008015~blood circulation	13	5.306122449	2.79E-05	207	186	13528	4.567658823	0.051820622	0.013214802	0.047718654
GOTERM_BP_FAT	GO:0048545~response to steroid hormone stimulus	13	5.306122449	3.82E-05	207	192	13528	4.424919485	0.070187539	0.014449069	0.0652545
GOTERM_BP_FAT	GO:0030855~epithelial cell differentiation	11	4.489795918	4.61E-05	207	137	13528	5.247293628	0.084076898	0.014530542	0.078744868
INTERPRO	IPR001664:Intermediate filament protein	8	3.265306122	5.87E-05	227	73	16659	8.042483857	0.03007448	0.015152032	0.084993754
GOTERM_BP_FAT	GO:0030216~keratinocyte differentiation	8	3.265306122	6.24E-05	207	66	13528	7.921534182	0.112124479	0.016845603	0.106616048
SP_PIR_KEYWORDS	gluconeogenesis	5	2.040816327	8.43E-05	245	19	19235	20.66058002	0.035435582	0.004499678	0.118663201
GOTERM_BP_FAT	GO:0009913~epidermal cell differentiation	8	3.265306122	1.09E-04	207	72	13528	7.261406334	0.188021038	0.025699115	0.186650608
GOTERM_BP_FAT	GO:0007507~heart development	13	5.306122449	1.13E-04	207	215	13528	3.951556005	0.193642739	0.023630585	0.192870624
GOTERM_MF_FAT	GO:0005198~structural molecule activity	23	9.387755102	1.60E-04	192	634	12983	2.453083925	0.072133224	0.072133224	0.228382003
GOTERM_BP_FAT	GO:0060429~epithelium development	13	5.306122449	1.87E-04	207	227	13528	3.742663176	0.300185697	0.035064507	0.319658439
UP_SEQ_FEATURE	sequence variant	180	73.46938776	1.88E-04	244	11992	19113	1.17576335	0.215480019	0.029879904	0.306404454
GOTERM_BP_FAT	GO:0009309~amine biosynthetic process	8	3.265306122	2.30E-04	207	81	13528	6.454583408	0.354739212	0.039044655	0.392199123
GOTERM_CC_FAT	GO:0005882~intermediate filament	11	4.489795918	3.30E-04	186	183	12782	4.130736236	0.083032313	0.021437637	0.430656614
GOTERM_BP_FAT	GO:0042755~eating behavior	5	2.040816327	3.68E-04	207	23	13528	14.20709935	0.504624436	0.056856271	0.628097242
GOTERM_CC_FAT	GO:0045111~intermediate filament cytoskeleton	11	4.489795918	3.91E-04	186	187	12782	4.042378242	0.097812895	0.020376211	0.511184296
GOTERM_BP_FAT	GO:0009725~response to hormone stimulus	16	6.530612245	5.04E-04	207	367	13528	2.849162158	0.617642221	0.071285337	0.858653399
SP_PIR_KEYWORDS	keratin	9	3.673469388	5.11E-04	245	144	19235	4.906887755	0.196603788	0.024029609	0.717827725
GOTERM_BP_FAT	GO:0010033~response to organic substance	24	9.795918367	6.34E-04	207	721	13528	2.175400511	0.701512681	0.082735246	1.078621813
GOTERM_BP_FAT	GO:0006941~striated muscle contraction	6	2.448979592	6.44E-04	207	46	13528	8.524259609	0.706894224	0.078557389	1.09476414
KEGG_PATHWAY	hsa05410:Hypertrophic cardiomyopathy (HCM)	8	3.265306122	7.90E-04	93	85	5085	5.146110057	0.088363687	0.088363687	0.899460286
PIR_SUPERFAMILY	PIRSF002282:cytoskeletal keratin	7	2.857142857	8.38E-04	137	61	7396	6.195046069	0.115228476	0.115228476	0.991713738
GOTERM_BP_FAT	GO:0003007~heart morphogenesis	7	2.857142857	8.45E-04	207	73	13528	6.266693137	0.800366224	0.095799743	1.434893224
GOTERM_BP_FAT	GO:0044057~regulation of system process	14	5.714285714	9.13E-04	207	309	13528	2.960961806	0.824657219	0.09734287	1.549533176

Architecture A3

Category	Term	Count	%	PValue	List Total	Pop Hits	Pop Total	Fold Enrichment	Bonferroni	Benjamini	FDR
SP_PIR_KEYWORDS	acetylated amino end	15	5.72519084	4.71E-11	252	100	19235	11.44940476	1.77E-08	1.77E-08	6.51E-08
SP_PIR_KEYWORDS	Secreted	53	20.22900763	3.91E-09	252	1689	19235	2.395178419	1.47E-06	7.33E-07	5.40E-06
GOTERM_CC_FAT	GO:0005576~extracellular region	58	22.13740458	7.57E-08	182	2010	12782	2.02655951	1.85E-05	1.85E-05	9.80E-05
SP_PIR_KEYWORDS	signal	76	29.00763359	2.19E-07	252	3250	19235	1.784932845	8.20E-05	2.73E-05	3.02E-04
GOTERM_CC_FAT	GO:0000786~nucleosome	10	3.816793893	2.30E-07	182	63	12782	11.14774115	5.64E-05	2.82E-05	2.98E-04
UP_SEQ_FEATURE	signal peptide	76	29.00763359	2.83E-07	252	3250	19113	1.773611722	2.47E-04	2.47E-04	4.39E-04
GOTERM_CC_FAT	GO:0044421~extracellular region part	35	13.35877863	5.22E-07	182	960	12782	2.560496795	1.28E-04	4.26E-05	6.75E-04
GOTERM_CC_FAT	GO:0032993~protein-DNA complex	10	3.816793893	3.38E-06	182	86	12782	8.166368515	8.28E-04	2.07E-04	0.004377507
GOTERM_CC_FAT	GO:0005615~extracellular space	27	10.30534351	4.00E-06	182	685	12782	2.768220101	9.80E-04	1.96E-04	0.005181699
SP_PIR_KEYWORDS	disulfide bond	65	24.80916031	1.40E-05	252	2924	19235	1.696788211	0.005221776	0.001308008	0.019288364
SP_PIR_KEYWORDS	methylation	14	5.34351145	1.86E-05	252	242	19235	4.415748393	0.006940117	0.001391893	0.025656958
UP_SEQ_FEATURE	disulfide bond	63	24.04580153	2.09E-05	252	2819	19113	1.695015963	0.01810888	0.009095807	0.032496087
GOTERM_MF_FAT	GO:0005179~hormone activity	10	3.816793893	2.98E-05	192	108	12983	6.261091821	0.011190428	0.011190428	0.04126773
GOTERM_BP_FAT	GO:0007586~digestion	9	3.435114504	5.70E-05	200	91	13528	6.68967033	0.0959405	0.0959405	0.096527753
GOTERM_BP_FAT	GO:0034728~nucleosome organization	9	3.435114504	6.66E-05	200	93	13528	6.545806452	0.111191982	0.057233847	0.112801667
SP_PIR_KEYWORDS	chromosomal protein	10	3.816793893	1.23E-04	252	145	19235	5.264094143	0.045221084	0.007682912	0.170358308
GOTERM_CC_FAT	GO:0000785~chromatin	12	4.580152672	1.30E-04	182	200	12782	4.213846154	0.031398318	0.005302859	0.168477363
GOTERM_BP_FAT	GO:0051046~regulation of secretion	12	4.580152672	1.99E-04	200	202	13528	4.018217822	0.297487479	0.111034944	0.33751765
INTERPRO	IPR005819:Histone H5	4	1.526717557	2.00E-04	229	9	16659	32.33187773	0.100035713	0.100035713	0.289717526
GOTERM_CC_FAT	GO:0016460~myosin II complex	5	1.908396947	2.33E-04	182	22	12782	15.96153846	0.055502942	0.008124347	0.301365667
GOTERM_BP_FAT	GO:0006334~nucleosome assembly	8	3.053435115	2.33E-04	200	84	13528	6.441904762	0.338354985	0.098104414	0.394694886
GOTERM_BP_FAT	GO:0031497~chromatin assembly	8	3.053435115	2.90E-04	200	87	13528	6.219770115	0.40158956	0.097598451	0.4904531
GOTERM_BP_FAT	GO:0006323~DNA packaging	9	3.435114504	3.30E-04	200	117	13528	5.203076923	0.442778958	0.092866404	0.55837957
GOTERM_BP_FAT	GO:0001525~angiogenesis	10	3.816793893	3.39E-04	200	148	13528	4.57027027	0.451509841	0.082220388	0.573415605
PIR_SUPERFAMILY	PIRSF038219:histone H1	4	1.526717557	3.40E-04	140	8	7396	26.41428571	0.04642901	0.04642901	0.399870759
GOTERM_BP_FAT	GO:0065004~protein-DNA complex assembly	8	3.053435115	3.82E-04	200	91	13528	5.946373626	0.491869346	0.081145124	0.646152393
SP_PIR_KEYWORDS	nucleosome core	6	2.290076336	3.94E-04	252	48	19235	9.541170635	0.137325106	0.020881383	0.542791391
SP_PIR_KEYWORDS	DNA binding	14	5.34351145	5.61E-04	252	340	19235	3.142973856	0.189773319	0.025962186	0.772379756
GOTERM_BP_FAT	GO:0032101~regulation of response to external stimulus	10	3.816793893	5.73E-04	200	159	13528	4.25408805	0.63709704	0.10651383	0.965854616
GOTERM_BP_FAT	GO:0006333~chromatin assembly or disassembly	9	3.435114504	5.73E-04	200	127	13528	4.793385827	0.637521107	0.09649976	0.966963339
GOTERM_BP_FAT	GO:0042593~glucose homeostasis	6	2.290076336	7.39E-04	200	49	13528	8.28244898	0.729580074	0.112093667	1.244401793
GOTERM_BP_FAT	GO:0033500~carbohydrate homeostasis	6	2.290076336	7.39E-04	200	49	13528	8.28244898	0.729580074	0.112093667	1.244401793
INTERPRO	IPR005818:Histone H1/H5	4	1.526717557	8.24E-04	229	14	16659	20.78477854	0.352289	0.195195055	1.188420743
SP_PIR_KEYWORDS	cleavage on pair of basic residues	12	4.580152672	9.03E-04	252	271	19235	3.379898085	0.287265162	0.036928202	1.240000277
SP_PIR_KEYWORDS	hormone	7	2.671755725	9.72E-04	252	87	19235	6.141443167	0.305632583	0.035818191	1.334958394

Architecture A4

Category	Term	Count	%	PValue	List Total	Pop Hits	Pop Total	Fold Enrichment	Bonferroni	Benjamini	FDR
GOTERM_CC_FAT	GO:0005576~extracellular region	59	19.73244147	3.05E-06	206	2010	12782	1.821325412	8.20E-04	8.20E-04	0.004005511
SP_PIR_KEYWORDS	Secreted	50	16.72240803	4.74E-06	288	1689	19235	1.977151997	0.001755188	0.001755188	0.006531993
GOTERM_CC_FAT	GO:0044421~extracellular region part	35	11.70568562	9.42E-06	206	960	12782	2.262186489	0.002529855	0.001265729	0.012373163
GOTERM_CC_FAT	GO:0005615~extracellular space	27	9.030100334	3.79E-05	206	685	12782	2.445709021	0.010153543	0.003396034	0.049840868
SP_PIR_KEYWORDS	ubl conjugation	23	7.692307692	7.81E-05	288	588	19235	2.612463388	0.028553725	0.014380259	0.107661155
GOTERM_CC_FAT	GO:0000785~chromatin	13	4.347826087	9.09E-05	206	200	12782	4.03315534	0.024154833	0.006094191	0.119372815
GOTERM_BP_FAT	GO:0031328~positive regulation of cellular biosynthetic process	26	8.695652174	2.00E-04	227	685	13528	2.261989131	0.308645888	0.308645888	0.339977902
GOTERM_BP_FAT	GO:0010557~positive regulation of macromolecule biosynthetic process	25	8.361204013	2.49E-04	227	654	13528	2.278085384	0.368102311	0.205080074	0.422631524
GOTERM_BP_FAT	GO:0009891~positive regulation of biosynthetic process	26	8.695652174	2.49E-04	227	695	13528	2.229442525	0.368959787	0.142266554	0.42387911
GOTERM_BP_FAT	GO:0045935~positive regulation of nucleobase, nucleoside, nucleotide and nucleic acid metabolic process	24	8.026755853	3.16E-04	227	624	13528	2.292104371	0.441886431	0.135667894	0.536644284
SP_PIR_KEYWORDS	isopeptide bond	15	5.016722408	3.31E-04	288	319	19235	3.140510711	0.115629785	0.040132319	0.455871452
GOTERM_BP_FAT	GO:0032101~regulation of response to external stimulus	11	3.678929766	3.38E-04	227	159	13528	4.122904718	0.464582045	0.11745149	0.574735532
GOTERM_BP_FAT	GO:0009628~response to abiotic stimulus	17	5.685618729	4.69E-04	227	368	13528	2.753016663	0.57965312	0.134498208	0.796458747
UP_SEQ_FEATURE	domain:CTCK	5	1.672240803	4.91E-04	288	25	19113	13.27291667	0.430158925	0.430158925	0.786325876
GOTERM_BP_FAT	GO:0051173~positive regulation of nitrogen compound metabolic process	24	8.026755853	4.92E-04	227	644	13528	2.220921006	0.596589071	0.121628498	0.834093433
GOTERM_BP_FAT	GO:0010604~positive regulation of macromolecule metabolic process	29	9.698996656	5.04E-04	227	857	13528	2.016623916	0.605331087	0.10971488	0.854136924
GOTERM_BP_FAT	GO:0007584~response to nutrient	10	3.344481605	5.70E-04	227	140	13528	4.256765261	0.650871178	0.110347028	0.966231304
INTERPRO	IPR006207:Cystine knot, C-terminal	5	1.672240803	5.83E-04	263	25	16659	12.66844106	0.275669968	0.275669968	0.848102476
GOTERM_MF_FAT	GO:0016836~hydro-lyase activity	6	2.006688963	7.01E-04	212	44	12983	8.350986278	0.252048037	0.252048037	0.978519622
GOTERM_BP_FAT	GO:0033273~response to vitamin	7	2.341137124	8.01E-04	227	66	13528	6.320651448	0.772303735	0.137546695	1.356023255

Architecture B1

Category	Term	Count	%	PValue	List Total	Pop Hits	Pop Total	Fold Enrichment	Bonferroni	Benjamini	FDR
NONE											

Architecture B2

Category	Term	Count	%	PValue	List Total	Pop Hits	Pop Total	Fold Enrichment	Bonferroni	Benjamini	FDR
INTERPRO	IPR007125:Histone core	7	3.664921466	3.69E-06	157	45	16659	16.50573248	0.001584942	0.001584942	0.005199203
SP_PIR_KEYWORDS	nucleosome core	7	3.664921466	4.65E-06	176	48	19235	15.93809186	0.001399439	0.001399439	0.006220181
SP_PIR_KEYWORDS	citrullination	5	2.617801047	4.26E-05	176	22	19235	24.83858471	0.012742437	0.006391645	0.056946768
GOTERM_CC_FAT	GO:0000786~nucleosome	7	3.664921466	4.54E-05	133	63	12782	10.67836257	0.009712536	0.009712536	0.057555185
KEGG_PATHWAY	hsa05322:Systemic lupus erythematosus	8	4.188481675	6.34E-05	54	99	5085	7.609427609	0.006072017	0.006072017	0.069851492
SP_PIR_KEYWORDS	methylation	11	5.759162304	7.70E-05	176	242	19235	4.967716942	0.022902637	0.007693247	0.102858908
GOTERM_BP_FAT	GO:0032582~negative regulation of gene-specific transcription	6	3.141361257	1.08E-04	135	48	13528	12.52592593	0.153491698	0.153491698	0.179377276
INTERPRO	IPR009072:Histone-fold	6	3.141361257	1.48E-04	157	54	16659	11.78980892	0.061465066	0.031219873	0.207715588
GOTERM_BP_FAT	GO:0006334~nucleosome assembly	7	3.664921466	1.82E-04	135	84	13528	8.350617284	0.245852362	0.131583258	0.303554526
GOTERM_BP_FAT	GO:0031497~chromatin assembly	7	3.664921466	2.21E-04	135	87	13528	8.062664964	0.289768458	0.107790893	0.367980358
GOTERM_CC_FAT	GO:0032993~protein-DNA complex	7	3.664921466	2.59E-04	133	86	12782	7.82252142	0.054131716	0.0274424	0.327738108
GOTERM_BP_FAT	GO:0065004~protein-DNA complex assembly	7	3.664921466	2.82E-04	135	91	13528	7.708262108	0.354236547	0.103565906	0.470076861
GOTERM_BP_FAT	GO:0034728~nucleosome organization	7	3.664921466	3.18E-04	135	93	13528	7.542493031	0.388662267	0.093732845	0.528807833
SP_PIR_KEYWORDS	chromosomal protein	8	4.188481675	3.69E-04	176	145	19235	6.029780564	0.105033375	0.027360932	0.491688114
GOTERM_BP_FAT	GO:0032583~regulation of gene-specific transcription	8	4.188481675	3.70E-04	135	134	13528	5.982531786	0.435742745	0.09096726	0.614658884
GOTERM_BP_FAT	GO:0006952~defense response	17	8.90052356	3.80E-04	135	615	13528	2.769960855	0.444671841	0.080594496	0.631737976
UP_SEQ_FEATURE	cross-link:Glycyl lysine isopeptide (Lys-Gly) (interchain with G-Cter in ubiquitin)	9	4.712041885	4.85E-04	175	199	19113	4.939468772	0.294361936	0.294361936	0.732093568
PIR_SUPERFAMILY	PIRSF002048:histone H2A	4	2.094240838	5.63E-04	84	15	7396	23.47936508	0.058439291	0.058439291	0.630591561
SP_PIR_KEYWORDS	Secreted	30	15.70680628	6.41E-04	176	1689	19235	1.941203778	0.175442397	0.037846863	0.853198415
INTERPRO	IPR002119:Histone H2A	4	2.094240838	6.99E-04	157	19	16659	22.33858532	0.259716662	0.095380403	0.980882744
SP_PIR_KEYWORDS	isopeptide bond	11	5.759162304	7.03E-04	176	319	19235	3.768612853	0.190678425	0.034645439	0.935300039

Architecture B3

Category	Term	Count	%	PValue	List Total	Pop Hits	Pop Total	Fold Enrichment	Bonferroni	Benjamini	FDR
SP_PIR_KEYWORDS	signal	118	29.7979798	1.14E-12	369	3250	19235	1.892624557	4.48E-10	4.48E-10	1.59E-09
UP_SEQ_FEATURE	signal peptide	118	29.7979798	1.76E-12	369	3250	19113	1.880620388	2.24E-09	2.24E-09	2.87E-09
SP_PIR_KEYWORDS	Secreted	76	19.19191919	3.42E-12	369	1689	19235	2.345577393	1.34E-09	6.70E-10	4.76E-09
GOTERM_CC_FAT	GO:0005576~extracellular region	83	20.95959596	2.88E-10	266	2010	12782	1.984262896	8.13E-08	8.13E-08	3.82E-07
SP_PIR_KEYWORDS	glycoprotein	132	33.33333333	4.87E-09	369	4318	19235	1.593518529	1.91E-06	6.36E-07	6.77E-06
UP_SEQ_FEATURE	glycosylation site:N-linked (GlcNAc...)	123	31.06060606	1.57E-07	369	4129	19113	1.542988617	1.99E-04	9.98E-05	2.55E-04
GOTERM_CC_FAT	GO:0005615~extracellular space	36	9.090909091	6.95E-07	266	685	12782	2.525393776	1.96E-04	9.80E-05	9.20E-04
GOTERM_MF_FAT	GO:0005125~cytokine activity	17	4.292929293	2.21E-06	264	195	12983	4.287315462	0.001251491	0.001251491	0.003241548
GOTERM_CC_FAT	GO:0044421~extracellular region part	43	10.85858586	2.92E-06	266	960	12782	2.152357456	8.22E-04	2.74E-04	0.003860425
SP_PIR_KEYWORDS	cytokine	15	3.787878788	1.07E-05	369	181	19235	4.319947896	0.004166942	0.001043367	0.014812917
SP_PIR_KEYWORDS	disulfide bond	88	22.22222222	1.17E-05	369	2924	19235	1.568812815	0.004586713	9.19E-04	0.016308457
KEGG_PATHWAY	hsa05322:Systemic lupus erythematosus	11	2.777777778	2.39E-05	103	99	5085	5.485436893	0.002459534	0.002459534	0.026680825
GOTERM_BP_FAT	GO:0006952~defense response	29	7.323232323	2.52E-05	264	615	13528	2.416309436	0.043650533	0.043650533	0.04272626
UP_SEQ_FEATURE	disulfide bond	83	20.95959596	6.38E-05	369	2819	19113	1.525055013	0.07796083	0.026693126	0.103783663
GOTERM_BP_FAT	GO:0006955~immune response	29	7.323232323	1.85E-04	264	690	13528	2.153667106	0.278749886	0.150735546	0.312395305
GOTERM_MF_FAT	GO:0008307~structural constituent of muscle	7	1.767676768	1.86E-04	264	42	12983	8.196338384	0.099739599	0.051179469	0.271614606
KEGG_PATHWAY	hsa00140:Steroid hormone biosynthesis	7	1.767676768	2.75E-04	103	46	5085	7.512663571	0.027909366	0.014053432	0.30625691
SP_PIR_KEYWORDS	polymorphism	251	63.38383838	9.62E-04	369	11550	19235	1.132811272	0.314339763	0.06095841	1.329890174

Architecture B4

Category	Term	Count	%	PValue	List Total	Pop Hits	Pop Total	Fold Enrichment	Bonferroni	Benjamini	FDR
SP_PIR_KEYWORDS	Secreted	57	15.28150134	5.45E-08	302	1689	19235	2.149465376	2.01E-05	2.01E-05	7.51E-05
GOTERM_CC_FAT	GO:0005576~extracellular region	62	16.62198391	1.54E-06	216	2010	12782	1.825327068	4.58E-04	4.58E-04	0.002057883
GOTERM_BP_FAT	GO:0051046~regulation of secretion	12	3.217158177	3.44E-04	213	202	13528	3.772974481	0.428763693	0.428763693	0.576105654
SP_PIR_KEYWORDS	signal	75	20.10723861	3.97E-04	302	3250	19235	1.469816607	0.136240548	0.0706134	0.545620605
UP_SEQ_FEATURE	signal peptide	75	20.10723861	4.80E-04	302	3250	19113	1.460494142	0.367297714	0.367297714	0.750730219
SP_PIR_KEYWORDS	plasma	8	2.144772118	6.44E-04	302	93	19235	5.478886278	0.211496087	0.07615038	0.883709457
UP_SEQ_FEATURE	disulfide bond	66	17.69436997	8.39E-04	302	2819	19113	1.481735809	0.551080132	0.329985173	1.30981559
GOTERM_BP_FAT	GO:0002526~acute inflammatory response	8	2.144772118	8.71E-04	213	98	13528	5.184631599	0.75753701	0.50759469	1.451360819
UP_SEQ_FEATURE	binding site:Substrate	14	3.753351206	9.07E-04	302	297	19113	2.983276473	0.579336173	0.25071842	1.415378237

Architecture B5

Category	Term	Count	%	PValue	List Total	Pop Hits	Pop Total	Fold Enrichment	Bonferroni	Benjamini	FDR
GOTERM_BP_FAT	GO:0009611~response to wounding	29	7.397959184	1.13E-06	260	530	13528	2.846966618	0.002083879	0.002083879	0.001922864
INTERPRO	IPR007125:Histone core	9	2.295918367	1.48E-06	309	45	16659	10.78252427	8.77E-04	8.77E-04	0.002185534
SP_PIR_KEYWORDS	nucleosome core	9	2.295918367	3.15E-06	368	48	19235	9.800441576	0.001204071	0.001204071	0.004369523
SP_PIR_KEYWORDS	glycoprotein	121	30.86734694	3.44E-06	368	4318	19235	1.464694681	0.001311882	6.56E-04	0.004761013
KEGG_PATHWAY	hsa05322:Systemic lupus erythematosus	12	3.06122449	3.94E-06	104	99	5085	5.926573427	4.72E-04	4.72E-04	0.004517476
UP_SEQ_FEATURE	glycosylation site:N-linked (GlcNAc...)	114	29.08163265	2.16E-05	368	4129	19113	1.433973117	0.024194537	0.024194537	0.034691565
GOTERM_CC_FAT	GO:0000786~nucleosome	9	2.295918367	4.09E-05	263	63	12782	6.942965779	0.010578132	0.010578132	0.05344887
GOTERM_BP_FAT	GO:0006952~defense response	28	7.142857143	5.05E-05	260	615	13528	2.36888055	0.089037685	0.045556542	0.085922538
INTERPRO	IPR016186:C-type lectin-like	10	2.551020408	6.55E-05	309	95	16659	5.675012775	0.038059987	0.019214594	0.096581859
GOTERM_MF_FAT	GO:0030246~carbohydrate binding	20	5.102040816	1.01E-04	262	354	12983	2.799629103	0.05120146	0.05120146	0.146247067
INTERPRO	IPR018378:C-type lectin, conserved site	9	2.295918367	1.36E-04	309	82	16659	5.91723893	0.077162377	0.026412258	0.199769915
INTERPRO	IPR001304:C-type lectin	9	2.295918367	1.36E-04	309	82	16659	5.91723893	0.077162377	0.026412258	0.199769915
UP_SEQ_FEATURE	domain:C-type lectin	9	2.295918367	1.36E-04	368	79	19113	5.91693038	0.143058671	0.074288744	0.218477622
GOTERM_BP_FAT	GO:0006955~immune response	29	7.397959184	1.42E-04	260	690	13528	2.186800446	0.231361019	0.0839747	0.242257723
GOTERM_CC_FAT	GO:0005576~extracellular region	65	16.58163265	1.48E-04	263	2010	12782	1.571666383	0.037798713	0.019081407	0.193524104
SP_PIR_KEYWORDS	disulfide bond	83	21.17346939	1.62E-04	368	2924	19235	1.483696582	0.060128136	0.020458404	0.224654601
UP_SEQ_FEATURE	disulfide bond	81	20.66326531	1.67E-04	368	2819	19113	1.492351011	0.172112231	0.061018241	0.267223172
GOTERM_BP_FAT	GO:0006954~inflammatory response	18	4.591836735	1.76E-04	260	325	13528	2.881704142	0.278126441	0.078245464	0.299962293
GOTERM_BP_FAT	GO:0006334~nucleosome assembly	9	2.295918367	2.01E-04	260	84	13528	5.574725275	0.310498222	0.071659969	0.342118465
SP_PIR_KEYWORDS	Secreted	54	13.7755102	2.17E-04	368	1689	19235	1.671123253	0.079437183	0.020479889	0.299744506
GOTERM_BP_FAT	GO:0031497~chromatin assembly	9	2.295918367	2.57E-04	260	87	13528	5.382493369	0.377734501	0.076019894	0.436327243
SMART	SM00034:CLECT	9	2.295918367	2.91E-04	190	82	9079	5.244608472	0.040444048	0.040444048	0.343303414
SP_PIR_KEYWORDS	signal	89	22.70408163	2.94E-04	368	3250	19235	1.431367057	0.106353156	0.022237931	0.406989449
GOTERM_BP_FAT	GO:0065004~protein-DNA complex assembly	9	2.295918367	3.50E-04	260	91	13528	5.145900254	0.475943517	0.088175711	0.59384346
UP_SEQ_FEATURE	signal peptide	89	22.70408163	3.64E-04	368	3250	19113	1.422288462	0.338224923	0.098060095	0.583143977
GOTERM_CC_FAT	GO:0032993~protein-DNA complex	9	2.295918367	3.74E-04	263	86	12782	5.086126094	0.092598022	0.031870975	0.487314251
GOTERM_BP_FAT	GO:0034728~nucleosome organization	9	2.295918367	4.05E-04	260	93	13528	5.035235732	0.527320365	0.089414225	0.688344489
INTERPRO	IPR009072:Histone-fold	7	1.785714286	4.66E-04	309	54	16659	6.988673139	0.241091183	0.066643837	0.684632111
INTERPRO	IPR000436:Sushi/SCR/CCP	7	1.785714286	5.68E-04	309	56	16659	6.73907767	0.285582313	0.065045579	0.833933649
INTERPRO	IPR016060:Complement control module	7	1.785714286	6.25E-04	309	57	16659	6.620848237	0.309340022	0.059820635	0.917415833
SP_PIR_KEYWORDS	sushi	7	1.785714286	6.73E-04	368	56	19235	6.533627717	0.226695476	0.041942095	0.928066165
GOTERM_BP_FAT	GO:0002684~positive regulation of immune system process	14	3.571428571	6.82E-04	260	238	13528	3.060633484	0.716309101	0.130629209	1.154606082
SP_PIR_KEYWORDS	citrullination	5	1.275510204	7.26E-04	368	22	19235	11.87932312	0.242398608	0.038880802	1.001755027
UP_SEQ_FEATURE	domain:Sushi 5	5	1.275510204	7.44E-04	368	22	19113	11.80397727	0.569629464	0.155171597	1.1873176
GOTERM_BP_FAT	GO:0006333~chromatin assembly or disassembly	10	2.551020408	7.47E-04	260	127	13528	4.096910963	0.748773787	0.129023384	1.265275961
UP_SEQ_FEATURE	short sequence motif:Antp-type hexapeptide	5	1.275510204	8.87E-04	368	23	19113	11.29076087	0.634079761	0.154271294	1.414159039
UP_SEQ_FEATURE	sequence variant	260	66.32653061	8.93E-04	368	11992	19113	1.126063209	0.636761259	0.134692817	1.424431007
INTERPRO	IPR001827:Homeobox protein, antennapedia type, conserved site	5	1.275510204	9.09E-04	309	24	16659	11.23179612	0.416315826	0.074030151	1.331775718

Architecture C1

Category	Term	Count	%	PValue	List Total	Pop Hits	Pop Total	Fold Enrichment	Bonferroni	Benjamini	FDR
INTERPRO	IPR013087:Zinc finger, C2H2-type/integrase, DNA-binding	14	100	2.38E-19	14	621	16659	26.82608696	9.50E-19	9.50E-19	9.07E-17
UP_SEQ_FEATURE	zinc finger region:C2H2-type 7	13	92.85714286	3.28E-19	14	451	19113	39.35207475	2.39E-17	2.39E-17	3.42E-16
UP_SEQ_FEATURE	zinc finger region:C2H2-type 10	12	85.71428571	2.97E-18	14	334	19113	49.04961506	2.17E-16	1.08E-16	3.10E-15
INTERPRO	IPR007087:Zinc finger, C2H2-type	14	100	5.05E-18	14	784	16659	21.24872449	2.02E-17	1.01E-17	1.93E-15
INTERPRO	IPR015880:Zinc finger, C2H2-like	14	100	6.26E-18	14	797	16659	20.902133	2.51E-17	8.35E-18	2.39E-15
UP_SEQ_FEATURE	zinc finger region:C2H2-type 11	11	78.57142857	1.68E-16	14	293	19113	51.25377864	1.62E-14	5.44E-15	2.33E-13
UP_SEQ_FEATURE	zinc finger region:C2H2-type 5	12	85.71428571	5.87E-16	14	538	19113	30.45087626	4.05E-14	1.01E-14	5.77E-13
UP_SEQ_FEATURE	zinc finger region:C2H2-type 4	12	85.71428571	1.22E-15	14	575	19113	28.49142857	8.92E-14	1.79E-14	1.28E-12
UP_SEQ_FEATURE	zinc finger region:C2H2-type 9	11	78.57142857	2.08E-15	14	376	19113	39.93977964	1.54E-13	2.56E-14	2.20E-12
INTERPRO	IPR001909:Krueppel-associated box	11	78.57142857	4.58E-15	14	355	16659	36.87102616	1.82E-14	4.55E-15	1.74E-12
UP_SEQ_FEATURE	zinc finger region:C2H2-type 8	11	78.57142857	5.89E-15	14	417	19113	36.01284687	4.30E-13	6.14E-14	6.14E-12
PIR_SUPERFAMILY	PIRSF005559:zinc finger protein ZFP-36	9	64.28571429	1.13E-14	9	137	7396	53.98540146	1.13E-14	1.13E-14	1.13E-12
SMART	SM00355:ZnF_C2H2	14	100	1.68E-14	14	797	9079	11.39146801	3.35E-14	3.35E-14	3.79E-12
SP_PIR_KEYWORDS	zinc-finger	14	100	2.21E-14	14	1718	19235	11.19615832	2.87E-13	2.87E-13	1.46E-11
UP_SEQ_FEATURE	zinc finger region:C2H2-type 6	11	78.57142857	2.87E-14	14	488	19113	30.77327283	2.10E-12	2.62E-13	3.00E-11
SP_PIR_KEYWORDS	dna-binding	14	100	6.58E-14	14	1868	19235	10.29710921	8.56E-13	4.28E-13	4.36E-11
UP_SEQ_FEATURE	domain:KRAB	10	71.42857143	8.69E-14	14	332	19113	41.12091222	6.35E-12	7.05E-13	9.07E-11
UP_SEQ_FEATURE	zinc finger region:C2H2-type 13	9	64.28571429	1.60E-13	14	201	19113	61.12899787	1.17E-11	1.17E-12	1.67E-10
SP_PIR_KEYWORDS	transcription regulation	14	100	1.90E-13	14	2026	19235	9.494076999	2.47E-12	8.22E-13	1.26E-10
SP_PIR_KEYWORDS	Transcription	14	100	2.53E-13	14	2071	19235	9.287783679	3.28E-12	8.21E-13	1.67E-10
UP_SEQ_FEATURE	zinc finger region:C2H2-type 2	11	78.57142857	2.59E-13	14	608	19113	24.69960056	1.89E-11	1.72E-12	2.70E-10
UP_SEQ_FEATURE	zinc finger region:C2H2-type 3	11	78.57142857	3.25E-13	14	622	19113	24.143661	2.37E-11	1.98E-12	3.39E-10
SP_PIR_KEYWORDS	zinc	14	100	5.20E-13	14	2189	19235	8.787117405	6.76E-12	1.35E-12	3.44E-10
UP_SEQ_FEATURE	zinc finger region:C2H2-type 1; degenerate	8	57.14285714	6.03E-13	14	122	19113	89.52224824	4.40E-11	3.39E-12	6.29E-10
UP_SEQ_FEATURE	zinc finger region:C2H2-type 12	9	64.28571429	9.94E-13	14	252	19113	48.75765306	7.25E-11	5.18E-12	1.04E-09
SMART	SM00349:KRAB	11	78.57142857	1.90E-12	14	355	9079	20.0943662	3.80E-12	1.90E-12	4.29E-10
SP_PIR_KEYWORDS	metal-binding	14	100	2.80E-11	14	2972	19235	6.472072678	3.64E-10	6.06E-11	1.85E-08
GOTERM_BP_FAT	GO:0006350~transcription	14	100	2.96E-11	14	2101	13528	6.438838648	4.74E-10	4.74E-10	2.10E-08
UP_SEQ_FEATURE	zinc finger region:C2H2-type 15	7	50	1.75E-10	14	134	19113	71.31716418	1.28E-08	8.51E-10	1.83E-07
GOTERM_MF_FAT	GO:0008270~zinc ion binding	14	100	1.75E-10	14	2311	12983	5.617914323	2.10E-09	2.10E-09	1.13E-07
GOTERM_MF_FAT	GO:0003677~DNA binding	14	100	1.96E-10	14	2331	12983	5.56971257	2.35E-09	1.18E-09	1.26E-07
UP_SEQ_FEATURE	zinc finger region:C2H2-type 14	7	50	4.57E-10	14	157	19113	60.86942675	3.33E-08	2.08E-09	4.77E-07
GOTERM_BP_FAT	GO:0045449~regulation of transcription	14	100	4.79E-10	14	2601	13528	5.201076509	7.66E-09	3.83E-09	3.40E-07
GOTERM_MF_FAT	GO:0046914~transition metal ion binding	14	100	1.99E-09	14	2785	12983	4.661759425	2.39E-08	7.97E-09	1.28E-06
SP_PIR_KEYWORDS	nucleus	14	100	3.26E-09	14	4283	19235	4.491010974	4.24E-08	6.06E-09	2.16E-06
UP_SEQ_FEATURE	zinc finger region:C2H2-type 16	6	42.85714286	5.12E-09	14	103	19113	79.52704577	3.74E-07	2.20E-08	5.35E-06
GOTERM_BP_FAT	GO:0006355~regulation of transcription, DNA-dependent	11	78.57142857	2.87E-07	14	1773	13528	5.995004432	4.59E-06	1.53E-06	2.04E-04
GOTERM_MF_FAT	GO:0046872~metal ion binding	14	100	3.48E-07	14	4140	12983	3.135990338	4.18E-06	1.04E-06	2.24E-04
GOTERM_BP_FAT	GO:0051252~regulation of RNA metabolic process	11	78.57142857	3.55E-07	14	1813	13528	5.862737373	5.68E-06	1.42E-06	2.52E-04
GOTERM_MF_FAT	GO:0043169~cation binding	14	100	3.93E-07	14	4179	12983	3.106724097	4.72E-06	9.44E-07	2.53E-04
GOTERM_MF_FAT	GO:0043167~ion binding	14	100	4.76E-07	14	4241	12983	3.061306296	5.71E-06	9.52E-07	3.06E-04
SP_PIR_KEYWORDS	zinc finger	5	35.71428571	2.60E-06	14	153	19235	44.89962652	3.38E-05	4.23E-06	0.00172302
SP_PIR_KEYWORDS	DNA binding	5	35.71428571	6.05E-05	14	340	19235	20.20483193	7.86E-04	8.73E-05	0.040015375
UP_SEQ_FEATURE	zinc finger region:C2H2-type 22	3	21.42857143	1.27E-04	14	25	19113	163.8257143	0.009228187	5.15E-04	0.132474366
UP_SEQ_FEATURE	zinc finger region:C2H2-type 21	3	21.42857143	1.38E-04	14	26	19113	157.5247253	0.009989585	5.28E-04	0.14345171
UP_SEQ_FEATURE	zinc finger region:C2H2-type 20	3	21.42857143	2.66E-04	14	36	19113	113.7678571	0.019201759	9.69E-04	0.276843401
UP_SEQ_FEATURE	zinc finger region:C2H2-type 3; degenerate	3	21.42857143	3.12E-04	14	39	19113	105.0164835	0.022521392	0.00108412	0.325174752
UP_SEQ_FEATURE	zinc finger region:C2H2-type 1	5	35.71428571	3.73E-04	14	542	19113	12.59422773	0.026859792	0.00123683	0.388551047

Architecture C2

Category	Term	Count	%	PValue	List Total	Pop Hits	Pop Total	Fold Enrichment	Bonferroni	Benjamini	FDR
GOTERM_BP_FAT	GO:0006414~translational elongation	43	11.05398458	4.27E-45	274	101	13528	21.01987425	6.20E-42	6.20E-42	7.07E-42
KEGG_PATHWAY	hsa03010:Ribosome	41	10.53984576	1.29E-44	119	87	5085	20.13764126	1.38E-42	1.38E-42	1.45E-41
GOTERM_CC_FAT	GO:0022626~cytosolic ribosome	39	10.02570694	1.45E-42	272	81	12782	22.62608932	4.44E-40	4.44E-40	1.95E-39
SP_PIR_KEYWORDS	ribosome	35	8.997429306	3.34E-39	370	73	19235	24.92502777	1.16E-36	1.16E-36	4.56E-36
SP_PIR_KEYWORDS	ribosomal protein	46	11.8251928	1.76E-36	370	188	19235	12.72009776	6.10E-34	3.05E-34	2.40E-33
GOTERM_CC_FAT	GO:0033279~ribosomal subunit	41	10.53984576	2.95E-36	272	128	12782	15.05233226	9.04E-34	4.52E-34	3.96E-33
GOTERM_BP_FAT	GO:0006412~translation	57	14.6529563	4.42E-36	274	331	13528	8.502172139	6.42E-33	3.21E-33	7.31E-33
GOTERM_MF_FAT	GO:0003735~structural constituent of ribosome	43	11.05398458	4.85E-34	269	168	12983	12.35327049	2.41E-31	2.41E-31	6.98E-31
SP_PIR_KEYWORDS	protein biosynthesis	44	11.31105398	5.51E-34	370	188	19235	12.16705003	1.91E-31	6.38E-32	7.53E-31
SP_PIR_KEYWORDS	ribonucleoprotein	50	12.85347044	7.15E-33	370	279	19235	9.316574639	2.48E-30	6.21E-31	9.77E-30
GOTERM_CC_FAT	GO:0005840~ribosome	46	11.8251928	2.73E-32	272	215	12782	10.05424077	8.35E-30	2.78E-30	3.66E-29
GOTERM_CC_FAT	GO:0044445~cytosolic part	38	9.768637532	3.65E-29	272	152	12782	11.74816176	1.12E-26	2.80E-27	4.90E-26
GOTERM_CC_FAT	GO:0030529~ribonucleoprotein complex	58	14.91002571	1.08E-25	272	515	12782	5.292375785	3.29E-23	6.59E-24	1.44E-22
GOTERM_CC_FAT	GO:0022625~cytosolic large ribosomal subunit	21	5.398457584	3.91E-24	272	38	12782	25.96962074	1.20E-21	2.00E-22	5.24E-21
GOTERM_CC_FAT	GO:0015934~large ribosomal subunit	23	5.912596401	7.16E-21	272	67	12782	16.13180421	2.19E-18	3.13E-19	9.60E-18
GOTERM_MF_FAT	GO:0005198~structural molecule activity	52	13.36760925	2.09E-17	269	634	12983	3.958556636	1.04E-14	5.18E-15	3.00E-14
GOTERM_CC_FAT	GO:0022627~cytosolic small ribosomal subunit	17	4.370179949	4.15E-17	272	40	12782	19.971875	1.27E-14	1.59E-15	5.56E-14
SP_PIR_KEYWORDS	acetylation	112	28.79177378	9.43E-17	370	2635	19235	2.209672291	3.85E-14	7.66E-15	1.55E-13
GOTERM_CC_FAT	GO:0015935~small ribosomal subunit	18	4.627249357	9.03E-15	272	63	12782	13.42647059	2.75E-12	3.06E-13	1.21E-11
GOTERM_MF_FAT	GO:0003723~RNA binding	49	12.59640103	3.17E-13	269	718	12983	3.293778671	1.57E-10	5.25E-11	4.56E-10
GOTERM_CC_FAT	GO:0005829~cytosol	64	16.45244216	5.21E-10	272	1330	12782	2.26130031	1.59E-07	1.59E-08	6.98E-07
GOTERM_CC_FAT	GO:0043228~non-membrane-bounded organelle	99	25.44987147	7.35E-10	272	2596	12782	1.792092473	2.25E-07	2.05E-08	9.85E-07
GOTERM_CC_FAT	GO:0043232~intracellular non-membrane-bounded organelle	99	25.44987147	7.35E-10	272	2596	12782	1.792092473	2.25E-07	2.05E-08	9.85E-07
GOTERM_BP_FAT	GO:0042254~ribosome biogenesis	16	4.11311054	2.28E-08	274	122	13528	6.475050856	3.31E-05	1.10E-05	3.77E-05
GOTERM_BP_FAT	GO:0022613~ribonucleoprotein complex biogenesis	19	4.884318766	2.40E-08	274	180	13528	5.211516626	3.49E-05	8.73E-06	3.98E-05
GOTERM_BP_FAT	GO:0006413~translational initiation	10	2.570694087	2.28E-07	274	45	13528	10.97161395	3.31E-04	6.63E-05	3.78E-04
GOTERM_BP_FAT	GO:0006364~rRNA processing	13	3.341902314	3.10E-07	274	92	13528	6.976515392	4.49E-04	7.49E-05	5.12E-04
GOTERM_BP_FAT	GO:0016072~rRNA metabolic process	13	3.341902314	4.96E-07	274	96	13528	6.685827251	7.20E-04	1.03E-04	8.21E-04
GOTERM_BP_FAT	GO:0042273~ribosomal large subunit biogenesis	6	1.542416452	7.49E-07	274	10	13528	29.62335766	0.001086351	1.36E-04	0.001238896
GOTERM_BP_FAT	GO:0034470~ncRNA processing	16	4.11311054	5.89E-06	274	187	13528	4.224364729	0.008518959	9.50E-04	0.009751089
SP_PIR_KEYWORDS	Initiation factor	9	2.313624679	6.12E-06	370	52	19235	8.997661123	0.002122901	3.54E-04	0.008365107
GOTERM_BP_FAT	GO:0034660~ncRNA metabolic process	17	4.370179949	1.76E-05	274	230	13528	3.649254205	0.025296193	0.002558885	0.02919946
GOTERM_MF_FAT	GO:0008135~translation factor activity, nucleic acid binding	11	2.827763496	3.37E-05	269	98	12983	5.417381079	0.016596275	0.004175144	0.04843791
GOTERM_MF_FAT	GO:0003743~translation initiation factor activity	9	2.313624679	3.39E-05	269	61	12983	7.120909257	0.016716431	0.003365868	0.048791486
GOTERM_MF_FAT	GO:0003729~mRNA binding	9	2.313624679	6.05E-05	269	66	12983	6.581446435	0.029632684	0.005000887	0.087045921
SP_PIR_KEYWORDS	rna-binding	25	6.426735219	1.30E-04	370	540	19235	2.406781782	0.044020421	0.006410611	0.177054501
GOTERM_CC_FAT	GO:0005852~eukaryotic translation initiation factor 3 complex	5	1.285347044	2.24E-04	272	15	12782	15.66421569	0.066350032	0.005704805	0.300264164
SP_PIR_KEYWORDS	diamond-blackfan anemia	4	1.028277635	2.31E-04	370	7	19235	29.70656371	0.077181888	0.009990154	0.315684099
SP_PIR_KEYWORDS	rrna-binding	5	1.285347044	2.60E-04	370	17	19235	15.29014308	0.08634254	0.009983118	0.354823565
GOTERM_BP_FAT	GO:0006396~RNA processing	25	6.426735219	3.01E-04	274	547	13528	2.256501955	0.354243364	0.038977547	0.497234363
UP_SEQ_FEATURE	transit peptide:Mitochondrion	22	5.655526992	3.14E-04	370	467	19113	2.433508884	0.26013601	0.26013601	0.492960584
SP_PIR_KEYWORDS	transit peptide	22	5.655526992	3.77E-04	370	476	19235	2.40273677	0.122726406	0.013008288	0.514091086
SP_PIR_KEYWORDS	blocked amino end	9	2.313624679	3.90E-04	370	92	19235	5.085634548	0.126447324	0.012214503	0.530735231
GOTERM_CC_FAT	GO:0005730~nucleolus	30	7.712082262	4.04E-04	272	698	12782	2.019741278	0.116316823	0.009466956	0.540174994
SP_PIR_KEYWORDS	mitochondrion	31	7.969151671	7.23E-04	370	832	19235	1.936996492	0.22192412	0.020693818	0.982901698

Architecture C3

Category	Term	Count	%	PValue	List Total	Pop Hits	Pop Total	Fold Enrichment	Bonferroni	Benjamini	FDR
UP_SEQ_FEATURE	domain:KRAB	19	8.260869565	4.60E-08	217	332	19113	5.04062795	2.99E-05	2.99E-05	6.87E-05
SMART	SM00349:KRAB	18	7.826086957	3.17E-07	102	355	9079	4.513173157	2.56E-05	2.56E-05	3.37E-04
INTERPRO	IPR001909:Krueppel-associated box	18	7.826086957	7.91E-07	193	355	16659	4.376589068	2.98E-04	2.98E-04	0.001093739
UP_SEQ_FEATURE	zinc finger region:C2H2-type 7	20	8.695652174	9.55E-07	217	451	19113	3.905913127	6.19E-04	3.10E-04	0.001425031
UP_SEQ_FEATURE	zinc finger region:C2H2-type 8	19	8.260869565	1.32E-06	217	417	19113	4.013161821	8.57E-04	2.86E-04	0.001971211
UP_SEQ_FEATURE	zinc finger region:C2H2-type 9	18	7.826086957	1.40E-06	217	376	19113	4.216516325	9.10E-04	2.28E-04	0.002094769
UP_SEQ_FEATURE	zinc finger region:C2H2-type 5	21	9.130434783	3.33E-06	217	538	19113	3.438002159	0.002160159	4.32E-04	0.004973905
UP_SEQ_FEATURE	zinc finger region:C2H2-type 4	21	9.130434783	8.95E-06	217	575	19113	3.216774194	0.005794899	9.68E-04	0.013366917
UP_SEQ_FEATURE	zinc finger region:C2H2-type 6	19	8.260869565	1.17E-05	217	488	19113	3.429279671	0.007558422	0.001083289	0.017449889
UP_SEQ_FEATURE	zinc finger region:C2H2-type 3	21	9.130434783	2.77E-05	217	622	19113	2.973706047	0.017836974	0.002247218	0.04138916
UP_SEQ_FEATURE	zinc finger region:C2H2-type 11	14	6.086956522	3.07E-05	217	293	19113	4.208521414	0.019740885	0.002212919	0.045850319
INTERPRO	IPR013087:Zinc finger, C2H2-type/integrase, DNA-binding	21	9.130434783	3.38E-05	193	621	16659	2.918900653	0.012661112	0.006350722	0.046724689
UP_SEQ_FEATURE	zinc finger region:C2H2-type 13	11	4.782608696	1.00E-04	217	201	19113	4.820207717	0.062845709	0.006469715	0.149184146
PIR_SUPERFAMILY	PIRSF005559:zinc finger protein ZFP-36	9	3.913043478	1.16E-04	82	137	7396	5.925226989	0.010079464	0.010079464	0.125768479
UP_SEQ_FEATURE	zinc finger region:C2H2-type 12	12	5.217391304	1.45E-04	217	252	19113	4.194206715	0.089619386	0.008499357	0.215732236
SMART	SM00355:ZnF_C2H2	22	9.565217391	1.47E-04	102	797	9079	2.456983295	0.011875912	0.005955691	0.157065427
INTERPRO	IPR007087:Zinc finger, C2H2-type	22	9.565217391	2.86E-04	193	784	16659	2.422134398	0.102259588	0.035319295	0.394885609
INTERPRO	IPR015880:Zinc finger, C2H2-like	22	9.565217391	3.56E-04	193	797	16659	2.382626559	0.125662491	0.033014911	0.491340791
UP_SEQ_FEATURE	zinc finger region:C2H2-type 14	9	3.913043478	4.23E-04	217	157	19113	5.049076873	0.240151787	0.022626475	0.629709679
UP_SEQ_FEATURE	zinc finger region:C2H2-type 10	13	5.652173913	4.36E-04	217	334	19113	3.428198902	0.24666325	0.021552276	0.649378836
UP_SEQ_FEATURE	zinc finger region:C2H2-type 15	8	3.47826087	8.34E-04	217	134	19113	5.258408419	0.418263967	0.037956495	1.238339333

Architecture C4

Category	Term	Count	%	PValue	List Total	Pop Hits	Pop Total	Fold Enrichment	Bonferroni	Benjamini	FDR
SP_PIR_KEYWORDS	nucleus	133	31.51658768	6.81E-08	390	4283	19235	1.531549896	2.20E-05	2.20E-05	9.21E-05
GOTERM_BP_FAT	GO:0006396~RNA processing	32	7.582938389	8.66E-08	264	547	13528	2.997728658	1.18E-04	1.18E-04	1.42E-04
GOTERM_CC_FAT	GO:0070013~intracellular organelle lumen	61	14.4549763	3.30E-07	227	1779	12782	1.930753554	1.02E-04	1.02E-04	4.43E-04
GOTERM_CC_FAT	GO:0043233~organelle lumen	61	14.4549763	7.24E-07	227	1820	12782	1.887258556	2.23E-04	1.12E-04	9.72E-04
SMART	SM00349:KRAB	23	5.450236967	1.05E-06	175	355	9079	3.361239437	1.30E-04	1.30E-04	0.00121429
GOTERM_CC_FAT	GO:0031974~membrane-enclosed lumen	61	14.4549763	1.40E-06	227	1856	12782	1.850652248	4.32E-04	1.44E-04	0.001884214
INTERPRO	IPR013087:Zinc finger, C2H2-type/integrase, DNA-binding	32	7.582938389	2.36E-06	331	621	16659	2.593458558	0.001447109	0.001447109	0.003494352
INTERPRO	IPR001909:Krueppel-associated box	23	5.450236967	2.53E-06	331	355	16659	3.260771882	0.001554088	7.77E-04	0.00375287
GOTERM_CC_FAT	GO:0031981~nuclear lumen	50	11.84834123	5.19E-06	227	1450	12782	1.941667933	0.001597468	4.00E-04	0.006963976
UP_SEQ_FEATURE	domain:KRAB	21	4.976303318	1.64E-05	389	332	19113	3.107853006	0.01592096	0.01592096	0.025850595
SMART	SM00355:ZnF_C2H2	34	8.056872038	1.65E-05	175	797	9079	2.213199498	0.002030054	0.001015543	0.019048254
UP_SEQ_FEATURE	zinc finger region:C2H2-type 9	22	5.213270142	3.10E-05	389	376	19113	2.87484275	0.029910593	0.015068832	0.048907258
GOTERM_BP_FAT	GO:0016071~mRNA metabolic process	21	4.976303318	3.74E-05	264	370	13528	2.908353808	0.04954766	0.025088548	0.061408835
INTERPRO	IPR007087:Zinc finger, C2H2-type	34	8.056872038	3.82E-05	331	784	16659	2.182648437	0.023196115	0.007792605	0.056615731
INTERPRO	IPR015880:Zinc finger, C2H2-like	34	8.056872038	5.29E-05	331	797	16659	2.147046894	0.031940851	0.00808268	0.078300561
UP_SEQ_FEATURE	zinc finger region:C2H2-type 7	24	5.687203791	5.38E-05	389	451	19113	2.614652386	0.051239037	0.017379993	0.084696523
GOTERM_BP_FAT	GO:0006397~mRNA processing	19	4.502369668	5.74E-05	264	321	13528	3.033040687	0.075011527	0.025656467	0.094210252
UP_SEQ_FEATURE	zinc finger region:C2H2-type 10	20	4.739336493	5.83E-05	389	334	19113	2.942136293	0.05545222	0.014161027	0.091859823
UP_SEQ_FEATURE	zinc finger region:C2H2-type 6	25	5.924170616	6.59E-05	389	488	19113	2.517094062	0.062464042	0.012817184	0.103851386
GOTERM_BP_FAT	GO:0006350~transcription	65	15.4028436	1.17E-04	264	2101	13528	1.585320122	0.146607629	0.038858811	0.191453979
UP_SEQ_FEATURE	zinc finger region:C2H2-type 1	26	6.161137441	1.28E-04	389	542	19113	2.356966012	0.117476232	0.02061285	0.201114889
UP_SEQ_FEATURE	zinc finger region:C2H2-type 4	27	6.398104265	1.30E-04	389	575	19113	2.30714653	0.119358411	0.017993935	0.204547254
UP_SEQ_FEATURE	zinc finger region:C2H2-type 8	22	5.213270142	1.35E-04	389	417	19113	2.59218435	0.124086765	0.01642465	0.21320188
GOTERM_BP_FAT	GO:0008380~RNA splicing	17	4.028436019	1.41E-04	264	284	13528	3.067328212	0.173822	0.037469001	0.230547407
GOTERM_BP_FAT	GO:0034660~ncRNA metabolic process	15	3.55450237	1.65E-04	264	230	13528	3.341897233	0.200508621	0.036609637	0.270138251
UP_SEQ_FEATURE	zinc finger region:C2H2-type 3	28	6.63507109	1.89E-04	389	622	19113	2.211805355	0.168786596	0.02033144	0.297367816
GOTERM_BP_FAT	GO:0051252~regulation of RNA metabolic process	57	13.507109	2.46E-04	264	1813	13528	1.611041468	0.284095892	0.046622322	0.403175592
GOTERM_MF_FAT	GO:0003677~DNA binding	69	16.3507109	2.49E-04	254	2331	12983	1.513032155	0.101463672	0.101463672	0.350775041
SP_PIR_KEYWORDS	mrna processing	16	3.791469194	2.82E-04	390	260	19235	3.035108481	0.08701711	0.044498619	0.380155116
UP_SEQ_FEATURE	zinc finger region:C2H2-type 5	25	5.924170616	2.85E-04	389	538	19113	2.283163387	0.243602975	0.027532757	0.448744274
SP_PIR_KEYWORDS	acetylation	79	18.72037915	2.97E-04	390	2635	19235	1.478679512	0.091551393	0.031498894	0.400903848
GOTERM_MF_FAT	GO:0008270~zinc ion binding	68	16.11374408	3.38E-04	254	2311	12983	1.504008559	0.134860536	0.069871265	0.474662836
UP_SEQ_FEATURE	zinc finger region:C2H2-type 11	17	4.028436019	3.48E-04	389	293	19113	2.850759364	0.288853537	0.030513575	0.547624334
GOTERM_BP_FAT	GO:0006355~regulation of transcription, DNA-dependent	55	13.03317536	4.61E-04	264	1773	13528	1.589584508	0.465497584	0.075315221	0.754353135
GOTERM_CC_FAT	GO:0000502~proteasome complex	7	1.658767773	7.05E-04	227	61	12782	6.461616235	0.195305427	0.042527709	0.942071729
UP_SEQ_FEATURE	zinc finger region:C2H2-type 2	26	6.161137441	7.13E-04	389	608	19113	2.101111149	0.502266097	0.056482915	1.117637774
GOTERM_CC_FAT	GO:0005654~nucleoplasm	30	7.109004739	8.85E-04	227	882	12782	1.915250682	0.238576839	0.044411282	1.180290911
SP_PIR_KEYWORDS	proteasome	7	1.658767773	9.11E-04	390	56	19235	6.165064103	0.254906517	0.070920899	1.223499489

Architecture C5

Category	Term	Count	%	PValue	List Total	Pop Hits	Pop Total	Fold Enrichment	Bonferroni	Benjamini	FDR
UP_SEQ_FEATURE	zinc finger region:C2H2-type 6	24	7.547169811	3.41E-06	303	488	19113	3.102256127	0.003744853	0.003744853	0.005449937
UP_SEQ_FEATURE	zinc finger region:C2H2-type 7	22	6.918238994	1.10E-05	303	451	19113	3.077034533	0.012033969	0.006035196	0.017585354
SP_PIR_KEYWORDS	nucleus	100	31.44654088	1.49E-05	303	4283	19235	1.482181839	0.004669991	0.004669991	0.020003262
UP_SEQ_FEATURE	domain:KRAB	18	5.660377358	2.32E-05	303	332	19113	3.419957056	0.025255986	0.008490547	0.037151862
GOTERM_BP_FAT	GO:0006396~RNA processing	24	7.547169811	2.72E-05	220	547	13528	2.697955792	0.041768408	0.041768408	0.045494082
SMART	SM00349:KRAB	18	5.660377358	2.79E-05	140	355	9079	3.288169014	0.003285395	0.003285395	0.031911197
INTERPRO	IPR001909:Krueppel-associated box	18	5.660377358	3.88E-05	258	355	16659	3.273960039	0.019457985	0.019457985	0.055905107
UP_SEQ_FEATURE	zinc finger region:C2H2-type 4	24	7.547169811	4.67E-05	303	575	19113	2.632871287	0.050167842	0.012785064	0.074738742
UP_SEQ_FEATURE	zinc finger region:C2H2-type 5	23	7.232704403	4.94E-05	303	538	19113	2.696694762	0.052904268	0.010812144	0.078926518
SMART	SM00355:ZnF_C2H2	28	8.805031447	6.21E-05	140	797	9079	2.278293601	0.007303692	0.003658539	0.071070489
INTERPRO	IPR007087:Zinc finger, C2H2-type	28	8.805031447	8.28E-05	258	784	16659	2.306063123	0.041129529	0.020780683	0.119453108
GOTERM_MF_FAT	GO:0008270~zinc ion binding	59	18.55345912	9.54E-05	204	2311	12983	1.624788946	0.039903198	0.039903198	0.134190475
INTERPRO	IPR013087:Zinc finger, C2H2-type/integrase, DNA-binding	24	7.547169811	9.84E-05	258	621	16659	2.495449949	0.048681871	0.016497977	0.14192734
INTERPRO	IPR015880:Zinc finger, C2H2-like	28	8.805031447	1.09E-04	258	797	16659	2.268448542	0.053875711	0.013749928	0.157483969
UP_SEQ_FEATURE	zinc finger region:C2H2-type 8	19	5.974842767	1.23E-04	303	417	19113	2.874112591	0.126711298	0.022328459	0.196621522
GOTERM_CC_FAT	GO:0031981~nuclear lumen	40	12.57861635	2.01E-04	193	1450	12782	1.826978739	0.055593627	0.055593627	0.266530386
GOTERM_BP_FAT	GO:0006397~mRNA processing	16	5.031446541	2.34E-04	220	321	13528	3.064967431	0.307148195	0.167622799	0.390587636
GOTERM_MF_FAT	GO:0046914~transition metal ion binding	66	20.75471698	2.73E-04	204	2785	12983	1.508216285	0.110013444	0.056609012	0.383589776
UP_SEQ_FEATURE	zinc finger region:C2H2-type 9	17	5.34591195	3.41E-04	303	376	19113	2.851985465	0.313111705	0.052240783	0.544097233
GOTERM_BP_FAT	GO:0016071~mRNA metabolic process	17	5.34591195	3.49E-04	220	370	13528	2.825257985	0.420929732	0.166490753	0.5809858
UP_SEQ_FEATURE	zinc finger region:C2H2-type 3	23	7.232704403	3.87E-04	303	622	19113	2.332510904	0.347250517	0.051923641	0.617719451
SP_PIR_KEYWORDS	zinc-finger	46	14.46540881	4.53E-04	303	1718	19235	1.699746808	0.133082417	0.06891591	0.608485565
SP_PIR_KEYWORDS	zinc	55	17.29559748	5.03E-04	303	2189	19235	1.595021311	0.146541554	0.051448766	0.674928526
GOTERM_BP_FAT	GO:0008380~RNA splicing	14	4.402515723	7.40E-04	220	284	13528	3.031241997	0.686389339	0.251662163	1.229137575
SP_PIR_KEYWORDS	mrna processing	13	4.088050314	8.74E-04	303	260	19235	3.174092409	0.240840476	0.066566762	1.17070498
UP_SEQ_FEATURE	zinc finger region:C2H2-type 10	15	4.716981132	9.22E-04	303	334	19113	2.832898559	0.637947783	0.106746804	1.464983877

Architecture D

Category	Term	Count	%	PValue	List Total	Pop Hits	Pop Total	Fold Enrichment	Bonferroni	Benjamini	FDR
UP_SEQ_FEATURE	domain:Ig-like V-type	26	1.987767584	3.63E-07	1248	124	19113	3.211189516	0.001038692	0.001038692	6.50E-04
SP_PIR_KEYWORDS	alternative splicing	569	43.50152905	5.76E-07	1249	7488	19235	1.170243353	3.57E-04	3.57E-04	8.54E-04
UP_SEQ_FEATURE	splice variant	565	43.19571865	2.21E-06	1248	7458	19113	1.160219988	0.006306046	0.003158009	0.003955478
INTERPRO	IPR013106:Immunoglobulin V-set	34	2.599388379	7.05E-05	1080	249	16659	2.1062249	0.103079356	0.103079356	0.117476593
SP_PIR_KEYWORDS	polymorphism	814	62.2324159	7.28E-05	1249	11550	19235	1.085355904	0.044038183	0.022267001	0.107858519
UP_SEQ_FEATURE	sequence variant	844	64.52599388	1.20E-04	1248	11992	19113	1.077866815	0.291923518	0.108694219	0.215617322
PIR_SUPERFAMILY	PIRSF005491:tumor associated protein MAGE	8	0.611620795	2.62E-04	487	21	7396	5.785469835	0.125578009	0.125578009	0.377968747
GOTERM_CC_FAT	GO:0009434~microtubule-based flagellum	8	0.611620795	5.17E-04	846	23	12782	5.255216363	0.225405237	0.225405237	0.740532354
PIR_SUPERFAMILY	PIRSF036923:CD300 antigen like protein	5	0.382262997	5.49E-04	487	7	7396	10.84775594	0.245285616	0.131257009	0.790999118
SP_PIR_KEYWORDS	alternative promoter usage	11	0.840978593	7.26E-04	1249	47	19235	3.604330273	0.362044315	0.139144674	1.071263474
UP_SEQ_FEATURE	domain:MAGE	9	0.688073394	8.24E-04	1248	32	19113	4.307316707	0.905748978	0.445921221	1.465941302
GOTERM_BP_FAT	GO:0042981~regulation of apoptosis	77	5.886850153	9.10E-04	895	804	13528	1.447588871	0.937122089	0.937122089	1.628203902

Architecture E

Category	Term	Count	%	PValue	List Total	Pop Hits	Pop Total	Fold Enrichment	Bonferroni	Benjamini	FDR
INTERPRO	IPR001909:Krueppel-associated box	53	4.02124431	3.90E-08	1094	355	16659	2.273417102	5.97E-05	5.97E-05	6.50E-05
UP_SEQ_FEATURE	zinc finger region:C2H2-type 10	51	3.869499241	4.52E-08	1263	334	19113	2.310730084	1.32E-04	1.32E-04	8.12E-05
SMART	SM00349:KRAB	53	4.02124431	9.63E-08	620	355	9079	2.1862199	2.72E-05	2.72E-05	1.27E-04
UP_SEQ_FEATURE	zinc finger region:C2H2-type 12	41	3.1107739	2.07E-07	1263	252	19113	2.462117785	6.06E-04	3.03E-04	3.72E-04
UP_SEQ_FEATURE	zinc finger region:C2H2-type 11	45	3.414264036	2.59E-07	1263	293	19113	2.324183441	7.57E-04	2.52E-04	4.66E-04
UP_SEQ_FEATURE	zinc finger region:C2H2-type 9	53	4.02124431	3.25E-07	1263	376	19113	2.133111386	9.49E-04	2.37E-04	5.84E-04
UP_SEQ_FEATURE	zinc finger region:C2H2-type 1; degenerate	26	1.972685888	3.28E-07	1263	122	19113	3.225069117	9.57E-04	1.91E-04	5.88E-04
UP_SEQ_FEATURE	zinc finger region:C2H2-type 7	60	4.552352049	3.72E-07	1263	451	19113	2.013261636	0.001085332	1.81E-04	6.67E-04
UP_SEQ_FEATURE	zinc finger region:C2H2-type 8	56	4.248861912	7.07E-07	1263	417	19113	2.032251633	0.00206273	2.95E-04	0.001269108
UP_SEQ_FEATURE	zinc finger region:C2H2-type 6	62	4.704097117	1.12E-06	1263	488	19113	1.922637358	0.00326302	4.08E-04	0.002008795
UP_SEQ_FEATURE	zinc finger region:C2H2-type 3	74	5.614567527	1.17E-06	1263	622	19113	1.800391046	0.003404622	3.79E-04	0.002096116
UP_SEQ_FEATURE	domain:KRAB	47	3.566009105	1.46E-06	1263	332	19113	2.142324643	0.00425543	4.26E-04	0.002621043
INTERPRO	IPR013087:Zinc finger, C2H2-type/integrase, DNA-binding	73	5.538694992	1.57E-06	1094	621	16659	1.790040537	0.00239598	0.001198709	0.002611294
INTERPRO	IPR015880:Zinc finger, C2H2-like	88	6.676783005	1.66E-06	1094	797	16659	1.681341594	0.002538679	8.47E-04	0.002767012
UP_SEQ_FEATURE	zinc finger region:C2H2-type 5	66	5.007587253	1.68E-06	1263	538	19113	1.856466724	0.004891914	4.46E-04	0.003014029
SMART	SM00355:ZnF_C2H2	88	6.676783005	4.62E-06	620	797	9079	1.616853523	0.001301819	6.51E-04	0.006114436
UP_SEQ_FEATURE	zinc finger region:C2H2-type 4	67	5.083459788	7.92E-06	1263	575	19113	1.763325416	0.022856927	0.001924995	0.014210486
UP_SEQ_FEATURE	zinc finger region:C2H2-type 13	32	2.427921093	8.60E-06	1263	201	19113	2.409236478	0.024813923	0.001930978	0.015442494
SP_PIR_KEYWORDS	zinc-finger	158	11.98786039	1.02E-05	1264	1718	19235	1.39951979	0.006106777	0.006106777	0.015055375
INTERPRO	IPR007087:Zinc finger, C2H2-type	83	6.297420334	1.67E-05	1094	784	16659	1.612106154	0.025249075	0.006372932	0.027834521
UP_SEQ_FEATURE	splice variant	561	42.56449165	3.38E-05	1263	7458	19113	1.13832426	0.094116075	0.007035427	0.060733672
UP_SEQ_FEATURE	zinc finger region:C2H2-type 2	67	5.083459788	4.67E-05	1263	608	19113	1.667618609	0.127583027	0.009057914	0.083853725
SP_PIR_KEYWORDS	alternative splicing	558	42.33687405	6.28E-05	1264	7488	19235	1.134001111	0.037018119	0.018683598	0.092674656
GOTERM_CC_FAT	GO:0044459~plasma membrane part	180	13.65705615	1.40E-04	811	2203	12782	1.287763072	0.066920884	0.066920884	0.201717455
GOTERM_MF_FAT	GO:0008270~zinc ion binding	192	14.56752656	1.80E-04	850	2311	12983	1.268987706	0.163813059	0.163813059	0.283249985
INTERPRO	IPR001978:Troponin	5	0.379362671	2.48E-04	1094	6	16659	12.68967093	0.316195804	0.073199302	0.412893891
SP_PIR_KEYWORDS	zinc	184	13.96054628	2.87E-04	1264	2189	19235	1.279137344	0.158260449	0.05581033	0.42258109
GOTERM_BP_FAT	GO:0002684~positive regulation of immune system process	32	2.427921093	3.12E-04	913	238	13528	1.992213315	0.622795437	0.622795437	0.563395297
UP_SEQ_FEATURE	zinc finger region:C2H2-type 17	16	1.213960546	3.15E-04	1263	83	19113	2.917208024	0.601351941	0.055859009	0.563662115
UP_SEQ_FEATURE	zinc finger region:C2H2-type 18	14	1.062215478	3.26E-04	1263	66	19113	3.21003383	0.613952763	0.054449492	0.583290208
GOTERM_CC_FAT	GO:0030424~axon	23	1.745068285	4.37E-04	811	159	12782	2.279862581	0.193811322	0.102119898	0.626072231
GOTERM_CC_FAT	GO:0042995~cell projection	67	5.083459788	6.24E-04	811	697	12782	1.515025643	0.264865034	0.097482372	0.892995013
SP_PIR_KEYWORDS	serine proteinase	12	0.91047041	6.54E-04	1264	54	19235	3.381680731	0.325021355	0.09359455	0.961525888
UP_SEQ_FEATURE	zinc finger region:C2H2-type 14	23	1.745068285	6.56E-04	1263	157	19113	2.216938742	0.852993163	0.101038745	1.171491171
SP_PIR_KEYWORDS	metal-binding	237	17.98179059	7.32E-04	1264	2972	19235	1.213513627	0.356156552	0.084294008	1.076423437
GOTERM_MF_FAT	GO:0046914~transition metal ion binding	221	16.76783005	7.73E-04	850	2785	12983	1.212057451	0.537131845	0.319655855	1.213907867
GOTERM_CC_FAT	GO:0005861~troponin complex	5	0.379362671	9.12E-04	811	8	12782	9.850493218	0.362244709	0.106358112	1.302692981

Architecture F

Category	Term	Count	%	PValue	List Total	Pop Hits	Pop Total	Fold Enrichment	Bonferroni	Benjamini	FDR
SP_PIR_KEYWORDS	phosphoprotein	751	43.06192661	5.32E-09	1695	7263	19235	1.173400803	3.05E-06	3.05E-06	7.81E-06
SP_PIR_KEYWORDS	alternative splicing	765	43.8646789	3.67E-08	1695	7488	19235	1.1593594	2.11E-05	1.05E-05	5.39E-05
UP_SEQ_FEATURE	splice variant	764	43.80733945	4.97E-08	1692	7458	19113	1.157176168	1.78E-04	1.78E-04	9.12E-05
GOTERM_CC_FAT	GO:0000139~Golgi membrane	33	1.892201835	7.40E-05	1076	186	12782	2.107596834	0.040221228	0.040221228	0.108021361
GOTERM_CC_FAT	GO:0044431~Golgi apparatus part	45	2.580275229	1.21E-04	1076	294	12782	1.818242167	0.06487524	0.03298151	0.176434879
GOTERM_BP_FAT	GO:0010212~response to ionizing radiation	16	0.917431193	1.28E-04	1167	60	13528	3.091231077	0.35185367	0.35185367	0.233505367
UP_SEQ_FEATURE	mutagenesis site	227	13.01605505	1.81E-04	1692	2045	19113	1.25389464	0.477711677	0.27730482	0.331591226
GOTERM_CC_FAT	GO:0005794~Golgi apparatus	104	5.963302752	2.14E-04	1076	872	12782	1.416783193	0.111978448	0.038813117	0.312171899
GOTERM_CC_FAT	GO:0005829~cytosol	147	8.428899083	3.46E-04	1076	1330	12782	1.312962238	0.174655445	0.046855332	0.504087665
GOTERM_BP_FAT	GO:0033554~cellular response to stress	73	4.185779817	4.77E-04	1167	566	13528	1.495096303	0.801028477	0.553937759	0.866663959
GOTERM_BP_FAT	GO:0006974~response to DNA damage stimulus	52	2.981651376	6.28E-04	1167	373	13528	1.616059142	0.880754188	0.507793085	1.139904607
GOTERM_BP_FAT	GO:0006281~DNA repair	42	2.408256881	7.18E-04	1167	284	13528	1.714327094	0.912133552	0.455552801	1.302521176
GOTERM_CC_FAT	GO:0012505~endomembrane system	92	5.275229358	8.09E-04	1076	782	12782	1.397550842	0.361958162	0.085950256	1.176047385

Architecture G1

Category	Term	Count	%	PValue	List Total	Pop Hits	Pop Total	Fold Enrichment	Bonferroni	Benjamini	FDR
GOTERM_CC_FAT	GO:0005739~mitochondrion	62	8.998548621	8.36E-05	440	1087	12782	1.656945722	0.032245933	0.032245933	0.116216919
GOTERM_MF_FAT	GO:0048037~cofactor binding	21	3.047895501	5.19E-04	458	249	12983	2.39072447	0.314550513	0.314550513	0.783392308
SP_PIR_KEYWORDS	mitochondrion	49	7.111756168	5.22E-04	676	832	19235	1.675784806	0.207453444	0.207453444	0.737326994
SP_PIR_KEYWORDS	phosphoprotein	297	43.10595065	5.61E-04	676	7263	19235	1.163552276	0.221072267	0.117431174	0.792075845

Architecture G2

Category	Term	Count	%	PValue	List Total	Pop Hits	Pop Total	Fold Enrichment	Bonferroni	Benjamini	FDR
SP_PIR_KEYWORDS	coiled coil	82	14.46208113	8.92E-04	548	2019	19235	1.425572029	0.28896311	0.28896311	1.229250889

Architecture H

Category	Term	Count	%	PValue	List Total	Pop Hits	Pop Total	Fold Enrichment	Bonferroni	Benjamini	FDR
GOTERM_CC_FAT	GO:0070013~intracellular organelle lumen	201	13.07742355	1.26E-12	899	1779	12782	1.606420475	6.97E-10	6.97E-10	1.84E-09
GOTERM_CC_FAT	GO:0043233~organelle lumen	201	13.07742355	1.09E-11	899	1820	12782	1.570231882	6.01E-09	3.00E-09	1.59E-08
GOTERM_CC_FAT	GO:0031974~membrane-enclosed lumen	203	13.20754717	2.05E-11	899	1856	12782	1.55509594	1.14E-08	3.79E-09	3.00E-08
GOTERM_CC_FAT	GO:0031981~nuclear lumen	168	10.93038386	2.21E-11	899	1450	12782	1.647329216	1.22E-08	3.06E-09	3.23E-08
GOTERM_BP_FAT	GO:0044265~cellular macromolecule catabolic process	103	6.701366298	1.11E-09	1042	725	13528	1.844442385	3.10E-06	3.10E-06	1.98E-06
SP_PIR_KEYWORDS	nucleus	425	27.65126871	1.47E-09	1482	4283	19235	1.287908005	7.75E-07	7.75E-07	2.14E-06
GOTERM_BP_FAT	GO:0009057~macromolecule catabolic process	108	7.026675342	1.92E-09	1042	781	13528	1.795306475	5.39E-06	2.69E-06	3.43E-06
GOTERM_BP_FAT	GO:0019941~modification-dependent protein catabolic process	86	5.59531555	2.59E-09	1042	574	13528	1.94514703	7.27E-06	2.42E-06	4.63E-06
GOTERM_BP_FAT	GO:0043632~modification-dependent macromolecule catabolic process	86	5.59531555	2.59E-09	1042	574	13528	1.94514703	7.27E-06	2.42E-06	4.63E-06
GOTERM_BP_FAT	GO:0030163~protein catabolic process	90	5.855562785	6.00E-09	1042	622	13528	1.878529417	1.68E-05	4.21E-06	1.07E-05
GOTERM_BP_FAT	GO:0051603~proteolysis involved in cellular protein catabolic process	87	5.660377358	1.02E-08	1042	600	13528	1.882495202	2.85E-05	5.71E-06	1.82E-05
GOTERM_BP_FAT	GO:0044257~cellular protein catabolic process	87	5.660377358	1.29E-08	1042	603	13528	1.873129554	3.62E-05	6.03E-06	2.31E-05
SP_PIR_KEYWORDS	phosphoprotein	660	42.94079375	1.97E-08	1482	7263	19235	1.179429207	1.03E-05	5.17E-06	2.85E-05
SP_PIR_KEYWORDS	cell cycle	71	4.619388419	3.12E-08	1482	461	19235	1.998947603	1.64E-05	5.47E-06	4.53E-05
SP_PIR_KEYWORDS	ubl conjugation pathway	76	4.944697463	3.65E-08	1482	509	19235	1.937937635	1.92E-05	4.80E-06	5.30E-05
GOTERM_BP_FAT	GO:0007049~cell cycle	102	6.636304489	8.06E-08	1042	776	13528	1.706492273	2.26E-04	3.23E-05	1.44E-04
SP_PIR_KEYWORDS	alternative splicing	673	43.78659727	8.76E-08	1482	7488	19235	1.166522757	4.61E-05	9.22E-06	1.27E-04
GOTERM_CC_FAT	GO:0005654~nucleoplasm	104	6.766428107	1.37E-07	899	882	12782	1.676501227	7.59E-05	1.52E-05	2.01E-04
UP_SEQ_FEATURE	splice variant	670	43.59141184	2.05E-07	1479	7458	19113	1.160948914	6.58E-04	6.58E-04	3.73E-04
SP_PIR_KEYWORDS	coiled coil	212	13.79310345	1.50E-06	1482	2019	19235	1.362835786	7.87E-04	1.31E-04	0.002170956
KEGG_PATHWAY	hsa04120:Ubiquitin mediated proteolysis	25	1.626545218	1.57E-05	351	137	5085	2.643645892	0.002506779	0.002506779	0.018940113
GOTERM_CC_FAT	GO:0005730~nucleolus	79	5.139882889	2.51E-05	899	698	12782	1.609202839	0.013805292	0.00231423	0.036705983
GOTERM_MF_FAT	GO:0016881~acid-amino acid ligase activity	33	2.147039688	2.86E-05	960	201	12983	2.220351368	0.028965549	0.028965549	0.045401517
GOTERM_MF_FAT	GO:0019787~small conjugating protein ligase activity	29	1.886792453	3.08E-05	960	166	12983	2.362619227	0.03114132	0.015693808	0.048865542
GOTERM_BP_FAT	GO:0006511~ubiquitin-dependent protein catabolic process	38	2.472348731	4.34E-05	1042	242	13528	2.038609794	0.114648977	0.015106125	0.077555868
GOTERM_BP_FAT	GO:0032446~protein modification by small protein conjugation	25	1.626545218	6.21E-05	1042	132	13528	2.458849532	0.159763197	0.019155437	0.110847496
GOTERM_MF_FAT	GO:0016879~ligase activity, forming carbon-nitrogen bonds	35	2.277163305	8.40E-05	960	231	12983	2.049084596	0.082597649	0.028327415	0.133101902
GOTERM_BP_FAT	GO:0070647~protein modification by small protein conjugation or removal	28	1.821730644	8.52E-05	1042	160	13528	2.271976967	0.212622857	0.023621337	0.152192509
GOTERM_CC_FAT	GO:0044451~nucleoplasm part	64	4.163955758	9.78E-05	899	555	12782	1.639555462	0.052637207	0.007694978	0.142701168
GOTERM_BP_FAT	GO:0006281~DNA repair	41	2.667534157	1.39E-04	1042	284	13528	1.874266714	0.323419449	0.034895144	0.248625768
GOTERM_BP_FAT	GO:0006259~DNA metabolic process	63	4.098893949	1.64E-04	1042	506	13528	1.616426301	0.369235359	0.03767387	0.293180597
SP_PIR_KEYWORDS	protein transport	61	3.968770332	1.77E-04	1482	485	19235	1.632420663	0.088829909	0.01320147	0.25616457
GOTERM_BP_FAT	GO:0031396~regulation of protein ubiquitination	20	1.301236174	1.94E-04	1042	100	13528	2.596545106	0.419900005	0.041023616	0.346359707
GOTERM_CC_FAT	GO:0000793~condensed chromosome	22	1.431359792	2.44E-04	899	129	12782	2.424778608	0.126400983	0.01674986	0.356241968
GOTERM_BP_FAT	GO:0051438~regulation of ubiquitin-protein ligase activity	17	1.106050748	2.45E-04	1042	78	13528	2.829568384	0.49740749	0.047953224	0.437381584
GOTERM_BP_FAT	GO:0043161~proteasomal ubiquitin-dependent protein catabolic process	20	1.301236174	2.54E-04	1042	102	13528	2.545632456	0.50925068	0.046346412	0.45250754
GOTERM_BP_FAT	GO:0010498~proteasomal protein catabolic process	20	1.301236174	2.54E-04	1042	102	13528	2.545632456	0.50925068	0.046346412	0.45250754
GOTERM_BP_FAT	GO:0046907~intracellular transport	76	4.944697463	3.32E-04	1042	657	13528	1.50180691	0.606263227	0.056590221	0.592106509
GOTERM_BP_FAT	GO:0051340~regulation of ligase activity	17	1.106050748	3.85E-04	1042	81	13528	2.724769555	0.6605735	0.061580965	0.686070633
SMART	SM00320:WD40	37	2.407286923	4.33E-04	688	266	9079	1.835564565	0.124078667	0.124078667	0.578602213
GOTERM_MF_FAT	GO:0017076~purine nucleotide binding	179	11.64606376	4.79E-04	960	1918	12983	1.262142097	0.388605122	0.115739045	0.757262568
GOTERM_BP_FAT	GO:0006974~response to DNA damage stimulus	48	3.122966818	5.34E-04	1042	373	13528	1.670699264	0.776523468	0.079876298	0.950187669
GOTERM_MF_FAT	GO:0000166~nucleotide binding	205	13.33767079	5.49E-04	960	2245	12983	1.234927153	0.431009876	0.106651432	0.867412841
GOTERM_BP_FAT	GO:0031398~positive regulation of protein ubiquitination	17	1.106050748	5.89E-04	1042	84	13528	2.627456357	0.808385224	0.083287602	1.047214316
GOTERM_BP_FAT	GO:0045184~establishment of protein localization	85	5.530253741	6.11E-04	1042	769	13528	1.435021677	0.820164238	0.082208982	1.087205366
GOTERM_BP_FAT	GO:0051301~cell division	40	2.602472349	6.14E-04	1042	295	13528	1.760369563	0.82121003	0.078708015	1.090880881
GOTERM_BP_FAT	GO:0006260~DNA replication	29	1.886792453	6.50E-04	1042	190	13528	1.981573896	0.838662215	0.079575852	1.155588068
SP_PIR_KEYWORDS	acetylation	246	16.00520494	6.92E-04	1482	2635	19235	1.211709393	0.305165945	0.044490219	0.998836633
GOTERM_BP_FAT	GO:0015031~protein transport	84	5.465191932	7.17E-04	1042	762	13528	1.431166594	0.866136763	0.083718848	1.273087088
INTERPRO	IPR001680:WD40 repeat	37	2.407286923	7.17E-04	1290	266	16659	1.796301801	0.715966566	0.715966566	1.206912847
GOTERM_BP_FAT	GO:0006508~proteolysis	110	7.156798959	7.46E-04	1042	1054	13528	1.354933404	0.876654076	0.083504789	1.324544972
GOTERM_BP_FAT	GO:0051443~positive regulation of ubiquitin-protein ligase activity	15	0.975927131	7.69E-04	1042	70	13528	2.782012613	0.884318401	0.082659492	1.364869035
GOTERM_BP_FAT	GO:0000278~mitotic cell cycle	47	3.05790501	8.14E-04	1042	370	13528	1.649157027	0.898162062	0.084111351	1.444938027
GOTERM_MF_FAT	GO:0030554~adenyl nucleotide binding	149	9.694209499	9.81E-04	960	1577	12983	1.277786805	0.634840391	0.154564547	1.544385364
